# Boron Nitride Nanomaterials Trigger Immunomodulatory Effects in Human Broncho‐Epithelial Cells by Modulating Eicosanoid Lipid Signaling

**DOI:** 10.1002/advs.202516401

**Published:** 2025-12-01

**Authors:** Govind Gupta, Jonas Bossart, Sènan Mickael D'Almeida, Luis Augusto Visani de Luna, Christoph Schwärzler, Tingting Fu, Vanesa Ayala‐Nunez, Alexander Gogos, Marija Buljan, Vera M Kissling, Emmanuel Flahaut, Miguel Garcia, Cyrill Bussy, Peter Wick, Tina Buerki‐Thurnherr

**Affiliations:** ^1^ Swiss Federal Laboratories for Materials Science and Technology (Empa) Laboratory for Particles‐Biology Interactions St. Gallen 9014 Switzerland; ^2^ SIB Swiss Institute of Bioinformatics Lausanne 1015 Switzerland; ^3^ ETH Zurich Department of Health Sciences and Technology (D‐HEST) Zurich 8093 Switzerland; ^4^ Flow Cytometry Core Facility School of Life Sciences Ecole Polytechnique Fédérale de Lausanne (EPFL) Lausanne 1015 Switzerland; ^5^ Viollier AG Allschwil 4123 Switzerland; ^6^ Centre for Nanotechnology in Medicine School of Biological Sciences Faculty of Biology Medicine and Health The University of Manchester Manchester M13 9PT UK; ^7^ National Graphene Institute The University of Manchester Manchester M13 9PL UK; ^8^ Lydia Becker Institute of Immunology and Inflammation Faculty of Biology, Medicine and Health The University of Manchester Manchester M13 9PT UK; ^9^ Department of Quantum Matter Physics Laboratory of Advanced Technology University of Geneva 1211 Geneva 4 Geneva 1205 Switzerland; ^10^ Nanoparticle Systems Engineering Laboratory Institute of Process Engineering Department of Mechanical and Process Engineering ETH Zurich Sonneggstrasse 3 Zurich 8092 Switzerland; ^11^ CIRIMAT Université Toulouse 3 Paul Sabatier Toulouse INP CNRS Université de Toulouse 118 Route de Narbonne Toulouse cedex 9 31062 France

**Keywords:** cytometry by time of flight, eicosanoids signaling, graphene, inflammatory mediators, lipidomics

## Abstract

For the successful commercial development of emerging 2D materials, it is crucial to understand their potential biological effects on healthy and diseased individuals. The present study demonstrates that a repeated low‐dose (1 µg cm^−2^ for 5 weeks) exposure of primary human broncho‐epithelial (HBE) cell cultures to hexagonal boron nitride nanosheets (*h*‐BN) and boron nitride nanotubes (BNNTs) increases the phospholipid, sphingolipid, and diglyceride content in cell membranes. Global lipidomics profiling further shows the induction of lipid mediator biosynthesis, especially after exposure to BNNTs in asthmatic cell cultures. The significant increase in leukotriene biosynthesis including its extracellular release is also confirmed in vivo in exposed mouse lungs. Mechanistically, extracellular release of lipid mediators prompts the recruitment and activation of immune cells. Mass cytometry‐based single‐cell profiling of human peripheral blood mononuclear cells reveals the activation of distinct lymphocyte populations expressing cytotoxic granzyme B and perforin mainly after exposure to conditioned medium from BNNT‐exposed asthmatic HBE cultures. These findings unveil the sub‐cytotoxic impact of BN nanomaterials on cellular lipid homeostasis and associated immunomodulation from repeated‐dose exposures, which may pose a potential health hazard, particularly for immune‐compromised individuals, and therefore, needs to be considered for the responsible and sustainable production and use of BN‐based products.

## Graphical abstract

This study demonstrates that BNNT exposure disrupts lipid homeostasis in bronchial epithelial cell cultures and activates eicosanoid lipid biosynthesis, producing inflammatory lipid mediators like leukotrienes. These effects are more pronounced in asthmatic cell cultures compared to healthy ones. Furthermore, the lipid mediators are released extracellularly, leading to immunomodulatory responses in human peripheral blood mononuclear cells.

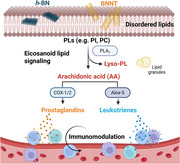

## 1 Introduction

Boron‐based nanomaterials including 2D materials have emerged in recent years as remarkable candidates for biomedical and industrial applications due to their unique properties, such as high neutron absorption, superior thermal conductivity, and exceptional mechanical strength, placing them as potential alternatives for carbon nanotubes and graphene‐based 2D materials.^[^ ^1^, ^2^, ^3^ ^]^ In therapeutic applications, boron neutron capture therapy (BNCT) has been successfully applied for various cancer treatments (including lung cancer) using elemental boron (B^10^)^[^ ^4^, ^5^, ^6^ ^]^ and boron nanomaterials.^[^ ^7^ ^]^ For industrial applications, boron nitride nanomaterials are being used in electronic devices, sensors, water purification, energy conversion, and photocatalysis.^[^ ^8^, ^9^, ^10^, ^11^ ^]^ Given these emerging industrial applications, the occupational exposure of boron nitride nanomaterials is on the rise and may pose inhalation‐related safety concerns in the lung or potential off‐target effects during therapeutic use. Understanding the safety profile of boron nanomaterials is therefore paramount for their commercial exploitation, including their biomedical and clinical application. Moreover, understanding the biological responses and underpinning mechanisms that result from their contact with biological systems will further pave the way for the safe and sustainable design of these nanomaterials. Inhalation is one of the most common routes of nanomaterial exposure in humans. In this respect, there have been few studies on pulmonary exposure of *h‐*BN both in vitro and in vivo, showing no acute toxicity in lung cells.^[^ ^12^, ^13^, ^14^ ^]^ In contrast, a recent study has reported nanomaterial shape as a crucial parameter in the cytotoxicity of *h*‐BNs, where authors could demonstrate that cornered‐edge *h*‐BNs were acute cytotoxic compounds, but not the round‐edge ones.^[^ ^15^ ^]^ On the other hand, BNNTs have been shown to trigger concerning effects in lungs, with similarities to the fibrogenic carbon nanotubes^[^ ^16^, ^17^ ^]^; and one of the concerns behind the pathogenicity of BNNTs is their high aspect ratio.^[^ ^14^ ^]^ Furthermore, the presence of impurities (i.e., toxic metals) in BNNTs carried from manufacturing processes could also play a crucial role in toxicity.^[^ ^18^ ^]^ However, most of the available studies focused on acute toxicities of *h*‐BNs or BNNTs with a limited understanding of their cellular mechanisms, subtoxic effects, and possible long‐term impacts that may progress to respiratory diseases. Boron‐containing compounds such as boronic acids have also been shown to trigger mild neurotoxic and gastrointestinal effects in mice at very high dose exposure (>100 mg kg^−1^ of body weight).^[^ ^19^ ^]^

In addition, there is increasing evidence that diseased individuals could mount a more pronounced response if exposed to nanomaterials. Previous studies using nanomaterials have demonstrated that inhalation exposure to copper oxide nanoparticles (CuO NPs) or multiwalled carbon nanotubes (MWCNTs) in healthy and asthmatic cells at occupationally relevant doses led to higher effects in asthmatic than in healthy lung cells.^[^ ^20^, ^21^ ^]^ Most recently, Areecheevakul et al. investigated the immunomodulatory response of CuO NP aerosol exposure in healthy vs house dust mite (HDM) asthmatic, or allergen immunotherapy (AIT)‐treated asthmatic mice (BALB/c, females).^[^ ^22^ ^]^ Their results demonstrated an increase in the number of type 2 immune cells (TH2, reflecting the severity of asthma) in AIT‐treated asthmatic mice upon CuO NPs exposure, indicating a crucial role of pre‐existing immune conditions on the effects of nanomaterials. Shurin et al. have reported that graphene oxide exposure in asthmatic (ovalbumin (OVA)‐challenged) mice augments airway hyper‐responsiveness and airway remodeling due to goblet cell hyperplasia and smooth muscle hypertrophy.^[^ ^23^ ^]^ However, reduced extracellular TH2 cytokines (interleukins (IL) such as IL‐4, IL‐5, and IL‐13) were detected in the bronchoalveolar lavage (BAL) fluid of these animals. In contrast, Beyeler et al. compared the effects of MWCNTs in broncho‐epithelial cells from COPD patients and healthy donors, and reported no acute toxic effects when analyzing the cell membrane integrity, monolayer cell barrier integrity, or a panel of inflammatory cytokines.^[^ ^24^ ^]^

In a recent study, we showed that *h*‐BN exposure of alveolar lung cell cultures (A549 cell line‐based air‐liquid interface model) did not trigger acute toxicity within 24 h, but elevated cellular lipid accumulation in lipid granules, which subsequently activated autophagy (a protective mechanism of cells to counteract lipid stress).^[^ ^13^ ^]^ Moreover, in silico‐based modeling studies have shown that BNNTs can extract lipids from the cell membrane.^[^ ^25^, ^26^, ^27^ ^]^ In addition, graphene oxide nanosheets have also been shown to affect lipid compositions in the cell membrane of immune cells, including oxidation of cholesterol.^[^ ^28^ ^]^ However, what happens thereafter at the biomolecular level is not known and remains to be studied. For example, do BNNTs or *h*‐BN exposures trigger specific changes in cell membrane lipid composition? How are lipidomic changes in the cell membrane sensed intracellularly and propagated further within the cell? Do lung cells from individuals with pre‐existing conditions (i.e., allergic asthma) respond differently to *h*‐BN and BNNTs exposure, since lipid dyshomeostasis plays a crucial role in lung pathogenesis and disease progression?

In the present study, we hypothesize that the effects of *h*‐BN or BNNTs on the ordering and arrangement of the cell membrane phospholipids (due to lipid extraction) can activate the eicosanoid lipid biosynthesis pathway, leading to an increased production of lipid intermediates and the subsequent activation of immune cells. The eicosanoid pathway allows the enzymatic release of arachidonic acid (a polyunsaturated fatty acid) from membrane‐bound phospholipids in the presence of an enzyme, namely phospholipase A2. Once arachidonic acid is released, it is further processed intracellularly and converted into lipid intermediates, either prostaglandins or leukotrienes, in the presence of cyclooxygenase 1 or 2 (COX1/2) or arachidonate 5‐lipoxygenase (Alox‐5) enzymes, respectively.^[^ ^29^ ^]^ These lipid intermediates are known to activate the peripheral immune response, leading to the exacerbation of asthma or other allergic diseases.^[^ ^30^, ^31^, ^32^ ^]^ Therefore, we further postulate that the effect of *h*‐BN and BNNTs will be higher in asthmatic cells than in healthy ones. In fact, targeting lipid intermediate biosynthesis pathways is one of the most popular therapeutic strategies in the treatment of allergic asthma.^[^ ^33^ ^]^ Allergic asthma is a chronic lung disease characterized by high levels of inflammation and obstruction of airways (due to hypermucus production). The inflammatory response in asthma is primarily driven by TH2 cells producing and releasing IL‐4, IL‐5, and IL‐13 as pro‐inflammatory mediators. However, several studies also suggested the involvement of other peripheral lymphocytes expressing granzymes, perforin, and interferon‐γ (IFN‐y) in the development and progression of asthma.^[^ ^34^, ^35^, ^36^, ^37^ ^]^

**Scheme 1:**
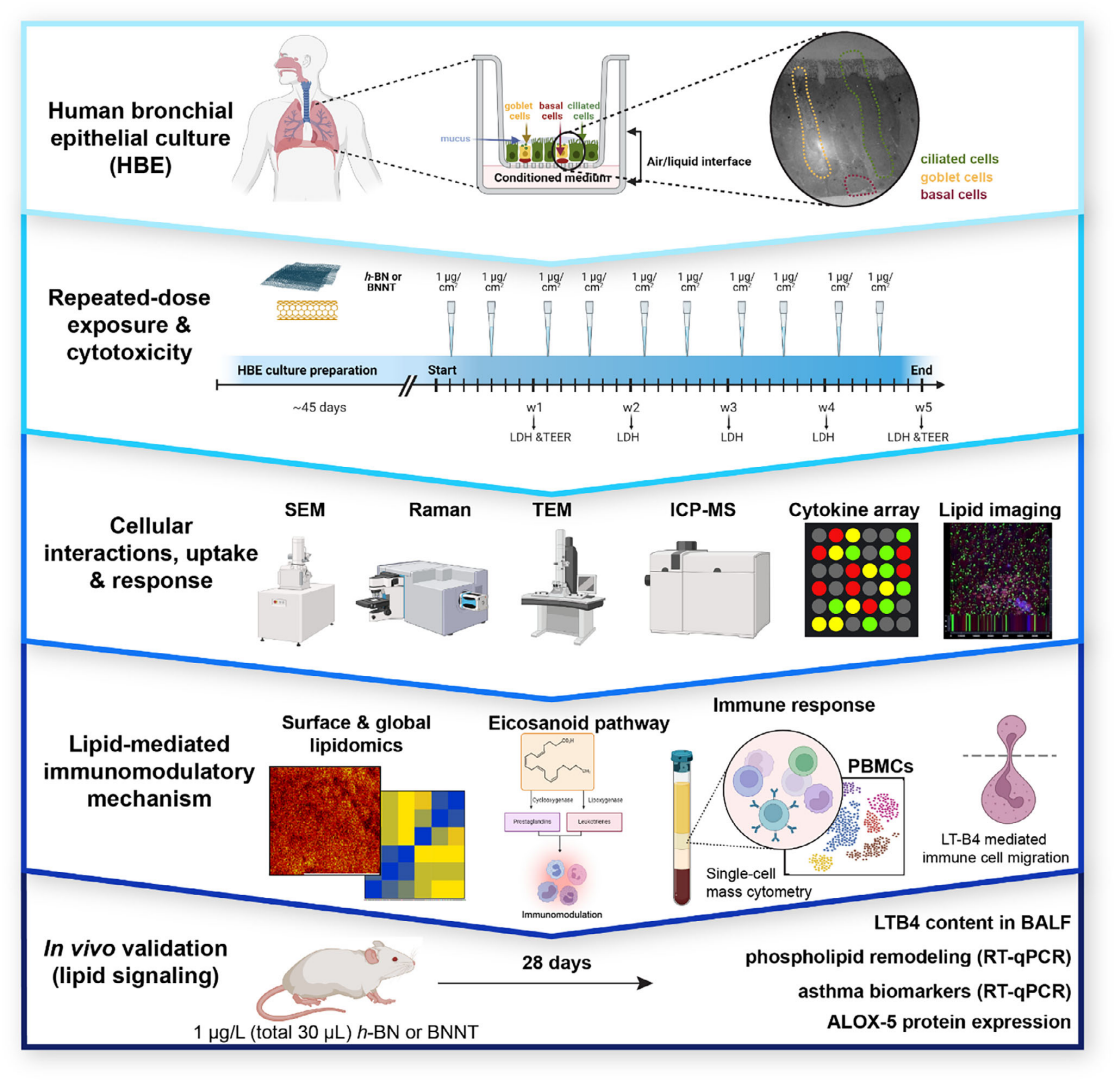
A schematic illustration of the study design. Air‐liquid interface (ALI)‐based advanced human broncho‐epithelial (HBE) cell cultures (MucilAir^TM^) reconstituted on a permeable membrane in a transwell insert using primary broncho‐epithelial cells of healthy or asthmatic donors (*n* = 3). Fully differentiated HBE cell cultures were exposed repeatedly (twice a week) to a low dose (1 µg cm^−2^; or 0.33 µg, applied during each exposure) for 5 weeks. During exposure, the integrity of the cell cultures was checked regularly by measuring LDH release and transepithelial electrical resistance (TEER). Following exposure, the samples were collected and processed to determine cellular interactions, cellular uptake, and toxicity of the two test materials. Lipidomic changes in the cell membrane or globally were studied, followed by investigating the immunomodulatory effects of conditioned medium from boron nanomaterials‐treated HBE cell culture on human PBMCs. In the end, lipid signaling was validated in vivo in mouse lungs at day 28 after a single dose (30 µg) of *h*‐BN and BNNTs applied by pharyngeal aspiration.

To test the above hypotheses, we employed air‐liquid interface‐based fully differentiated human broncho‐epithelial (HBE) cell cultures, reconstituted using primary cells derived from healthy and asthmatic donors. Since bronchial epithelial cells are highly fragile and the most affected cells in asthmatic disease,^[^ ^38^ ^]^ an HBE model was used in this study. These advanced cell cultures maintain in vivo‐like lung conditions and retain major cell types from the broncho‐epithelium, such as the pseudostratified structure of basal and ciliated cells, as well as mucus‐producing goblet cells. In addition, these cell cultures also present a well‐developed lung epithelial barrier, secrete a mucus layer, and contain functional cilia on the surface, as typically found in human bronchi (refer to Video S1, Supporting Information for beating cilia), thereby recapitulating the active mucociliary clearance mechanism in a dish. These cell cultures were then repeatedly (twice a week) exposed either to vehicle medium (negative control) or *h*‐BN and BNNTs (1 µg cm^−2^; 0.33 µg in 20 µL cell medium) for 5 weeks. Following exposure, the cell cultures including conditioned medium (from the basolateral side) were processed to assess cytotoxicity, lipid signaling, and their immunomodulatory effects on human peripheral blood mononuclear cells (PBMCs; **Scheme** **1**). The lipid signaling was further validated in vivo 28 days after a single dose (30 µg; oropharyngeal aspiration) pulmonary exposure of *h*‐BN and BNNTs in mice. Our in vitro results in HBE cell cultures showed that BNNT exposure strongly dysregulated the cell membrane lipid profile, which subsequently activated lipid intermediate (i.e., leukotrienes) biosynthesis, especially in asthmatic cell cultures. The secreted lipid intermediates from these lung cells were then shown to activate lymphocytes (T cells, B cells, and NK cells) as evidenced by their expression of granzyme B, perforin and IFN‐γ, which was determined by single‐cell mass cytometry of human PBMCs incubated with the conditioned medium from *h*‐BN and BNNT‐exposed HBE cell cultures. Finally, we demonstrated that *h*‐BN and BNNT‐exposed HBE‐conditioned medium from healthy and asthmatic cell cultures induced migration of neutrophil‐like cells, which was inhibited in the cells pre‐incubated with leukotriene‐B4 receptor pharmacological inhibitor, establishing a potential role of leukotrienes in BNNT‐mediated immunomodulatory effects in lung cells. These findings from a comprehensive analysis of the effects of boron nanomaterials on primary human bronchial airway epithelium upon repeated low‐dose exposure are revealing a pronounced dysregulation of lipid homeostasis and signaling in healthy and even more so in asthmatic lung cell cultures, and further indicating a possible lipid‐mediated immunomodulatory mechanism, which could lead to either pulmonary disease development or its progression, in the two respective models.

## 2 Results And Discussion

*h*‐BN nanosheets obtained from BeDimensional Spa (Italy) were prepared via liquid‐phase exfoliation of bulk *h*‐BN in water, using sodium cholate as an exfoliating agent. BNNTs were synthesized and obtained from BNNT LLC (USA) and purified to remove non‐nanotube boron species. BN nanomaterials (*h*‐BN and BNNTs) were free of metal impurities, as shown previously in another study using X‐ray photoelectron spectroscopy.^[^ ^14^ ^]^ The main atoms detected were C, N, O, and Na. TEM analysis showed a round‐shaped flake appearance of *h*‐BN nanosheets with their lateral size ranging from 100 to 400 nm (Figure S1a, Supporting Information). A similar size distribution of *h*‐BNs was also reported previously.^[^ ^13^ ^]^ BNNTs appeared like tubular bundle structures with inner and outer diameters of the nanotubes in the range of 1–7 nm and 2–8 nm, respectively (Figure S1b, Supporting Information). For the experiments, a uniform colloidal dispersion of *h‐*BN and BNNTs (0.5 mg mL^−1^) was prepared in 0.1% BSA‐water and stored at 4 °C for further use within a month. The nanomaterial dispersions in endotoxin‐free distilled water and cell culture medium were further characterized regarding ion release (dissolution), hydrodynamic size, and ζ‐potential. The results are presented in Figure S1c–f (Supporting Information). No significant release of boron ions (or dissolution) was detected either from *h‐*BN or BNNTs in both cell culture medium and water for up to 3 days when compared to day 0 of incubation at 37 °C (Figure S1c,d, Supporting Information). For BNNTs, the observed B content in the supernatant (after centrifugation) is likely from residual BNNTs that remain loosely stuck on the microcentrifuge tubes. Since nanomaterials exposure was renewed by adding freshly prepared suspensions twice a week, no dissolution of materials at later time points was investigated. The hydrodynamic diameter of *h*‐BN (16.5 µg mL^−1^) was further measured in water and culture medium to be 666 ± 87 nm and 680 ± 16 nm, respectively (Figure S1e, Supporting Information). The ζ‐potential of *h*‐BN was −13.6 ± 0.2 mV in water and −11.2 ± 1.0 mV in culture medium (Figure S1f, Supporting Information). The hydrodynamic diameter of BNNTs was 255.6 ± 158 nm and 158 ± 85 nm in water and culture medium, respectively (Figure S1e, Supporting Information). The decrease in average hydrodynamic size of BNNT in suspension in cell culture medium compared to water could be due to the adsorption of proteins (from cell culture medium) on the surface of the particles. Such a protein corona formation can be both stabilizing and destabilizing the colloidal stability of NP suspensions, depending on several factors such as protein composition, NP material type and experimental conditions. For an albumin‐rich corona, likely formed during the dispersion of *h*‐BN and BNNT (0.1% bovine serum albumin) has been previously reported to enhance nanoparticle colloidal stability^[^ ^39^, ^40^, ^41^, ^42^ ^]^ commonly through steric stabilization.^[^ ^40^ ^]^ The ζ‐potential of BNNTs was −38.7 ± 1.8 mV in water and −9.9 ± 0.7 mV in the culture medium (Figure S1f, Supporting Information). The reduction in ζ‐potential of BNNTs in cell culture medium is likely due to the formation of biomolecule corona on the particle's surface, as shown by others for nanomaterials.^[^ ^43^, ^44^, ^45^ ^]^ No endotoxin contamination (<0.5 EU mL^−1^) was found in either *h‐*BN (0.137 EU/mL) or BNNT (0.095 EU/mL) dispersions as determined by LAL assay. Raman spectra of *h*‐BN and BNNT suspensions recorded after drying showed the presence of their characteristic peak ≈1365 cm^−1^ (Figure S1g,h, Supporting Information), as also shown by others in previous studies.^[^ ^46^, ^47^ ^]^ The additional characterization data of the same *h*‐BN and BNNT materials used here can be found in another study.^[^ ^14^ ^]^

### 2.1 *h‐*BN and BNNTs Escape the Mucociliary Region and Enter Bronchial Epithelial Cells

**Figure 1:**
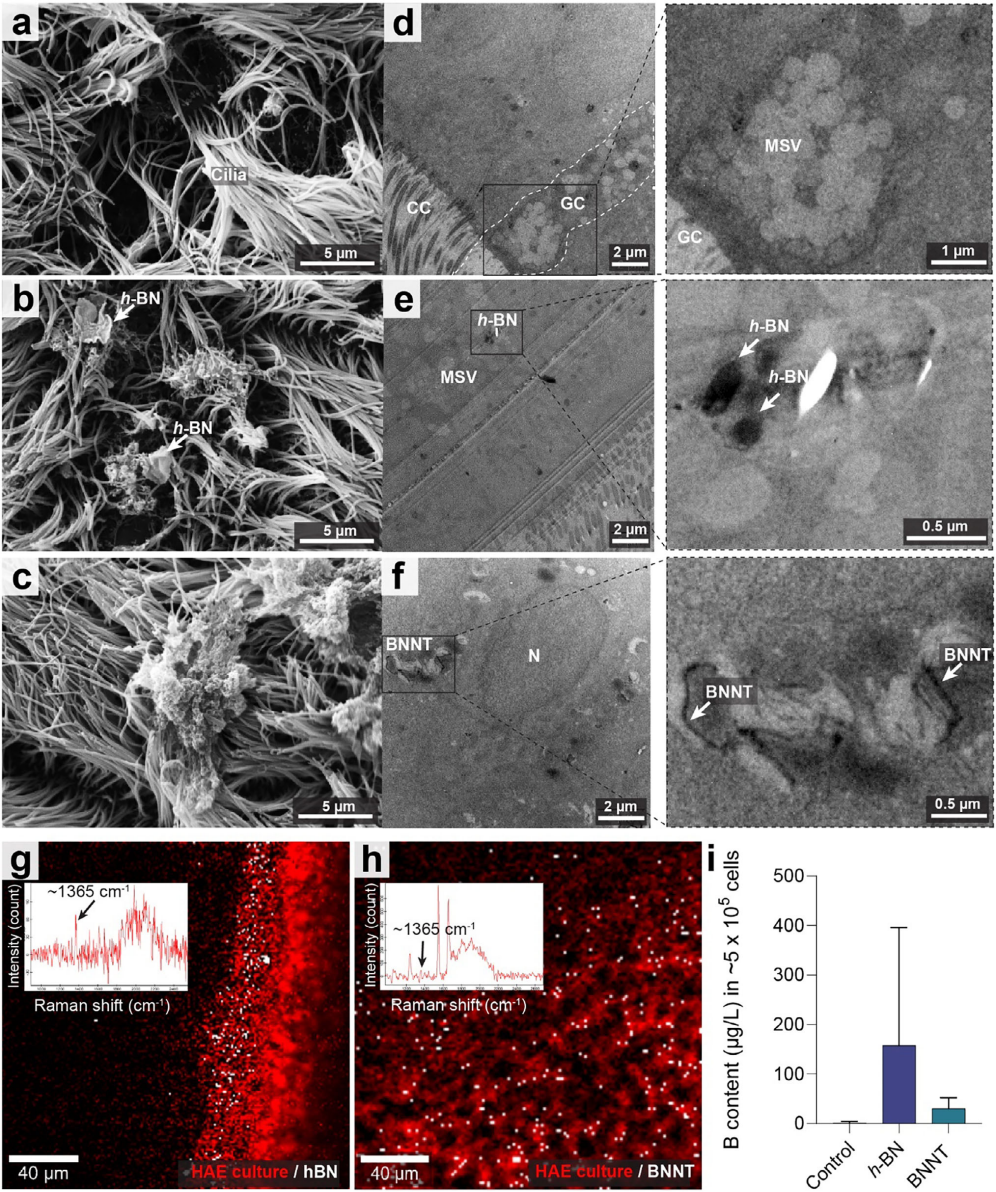
Ciliary interactions and cellular uptake of *h*‐BN and BNNTs in healthy HBE cultures after 5 weeks of repeated exposure. a–c) SEM images of HBE cultures with no treatment (control) (a) or exposed to *h*‐BN (b) or BNNTs (c). d–f) TEM images of HBE cultures with no treatment (control) (d) or exposed to *h*‐BN (e) or BNNTs (f). TEM images on the right side are the magnified views of the indicated region of interest in the corresponding image. More representative micrographs are shown in Figure S2 (Supporting Information). Abbreviations: MSV – Mucus‐secreting vesicles; CC – ciliated cells; GC ‐ Goblet cells, N – Nucleus. g–h) Raman spectroscopy images of HBE cultures after *h*‐BN (g), or BNNTs (h) exposure. (i) Cellular boron (B) content in HBE cell cultures (≈5 × 10^5^ cells/HBE cell culture) determined using ICP‐MS. The data presented here as "mean + SD" (*n* = 3).

Nanomaterial‐cell interaction is the first step toward understanding cellular behavior and biological responses during long‐term exposure assessment. Therefore, we first characterized cell surface interactions and cellular uptake of *h‐*BN and BNNTs in lung cell cultures by scanning and transmission electron microscopy (SEM, TEM) as well as Raman spectroscopy imaging and further quantified the cellular boron content using inductively coupled plasma mass spectrometry (ICP‐MS; **Figure** **1**). SEM images of HBE cell cultures showed the presence of a few *h‐*BN nanosheets in the ciliary region; however, BNNTs were visually mostly undetectable (Figure 1a–c). This was in accordance with TEM revealing a higher occurrence of *h‐*BN nanosheets in the ciliary region than BNNTs (Figure S2, Supporting Information).

The mucus layer and respiratory cilia are the first contact point in the bronchial airways during the inhalation of nanomaterials.^[^ ^48^, ^49^ ^]^ The mucus layer is made from mucin proteins that create a polymeric network with an intramolecular spacing of 400 nm.^[^ ^50^ ^]^ Hence, a fraction of particles <400 nm can pass through it and enter the broncho‐epithelial cells, especially the non‐ciliated cells. TEM images of HBE cell culture sections showed that both *h*‐BN and BNNTs were taken up by the cells (Figure 1d–f; Figure S2, Supporting Information). Moreover, it was evident in TEM images that *h*‐BN or BNNTs preferentially entered goblet cells (mucus‐secreting cells), especially *h*‐BN, possibly due to the absence of dense cilia on the surface of these cells as also shown for graphene‐based 2D materials, where the presence of cilia in differentiated intestinal cells (CaCo‐2 cell line) diminished the graphene oxide uptake in cells.^[^ ^51^ ^]^ Raman spectroscopy images reconstructed by co‐localizing *h*‐BN or BNNTs (≈1365 cm^−1^) with cell‐specific signal (2800–3100 cm^−1^) from the recorded large‐area scan map further revealed the presence of intact *h*‐BN and BNNTs within the cells (Figure 1g,h). The reference spectrum of intact *h*‐BN and BNNTs was also recorded and presented in Figure S1g,h (Supporting Information). Moreover, boron content in the *h*‐BN or BNNT‐exposed HBE cell cultures was found to be higher than in the controls, further confirming their presence in the cells after 5 weeks of repeated exposure. Previous studies on acute and chronic inhalation of nanomaterials (i.e., CuO, MWCNTs) in HBE cell cultures have also demonstrated the uptake of these materials in cells.^[^ ^20^, ^21^ ^]^ Moreover, the airway translocation and deposition of boron nanomaterials in lungs, and uptake in broncho‐alveolar lavage cells have been reported in mice.^[^ ^14^, ^15^, ^16^, ^17^, ^18^, ^19^, ^20^, ^21^, ^22^, ^23^, ^24^, ^25^, ^26^, ^27^, ^28^, ^29^, ^30^, ^31^, ^32^, ^33^, ^34^, ^35^, ^36^, ^37^, ^38^, ^39^, ^40^, ^41^, ^42^, ^43^, ^44^, ^45^, ^46^, ^47^, ^48^, ^49^, ^50^, ^51^, ^52^ ^]^

### 2.2 *h‐*BN and BNNTs Altered the Cell Membrane Lipid Composition of Bronchial Epithelial Cells Leading to the Activation of Eicosanoid Lipid Signaling

**Figure 2:**
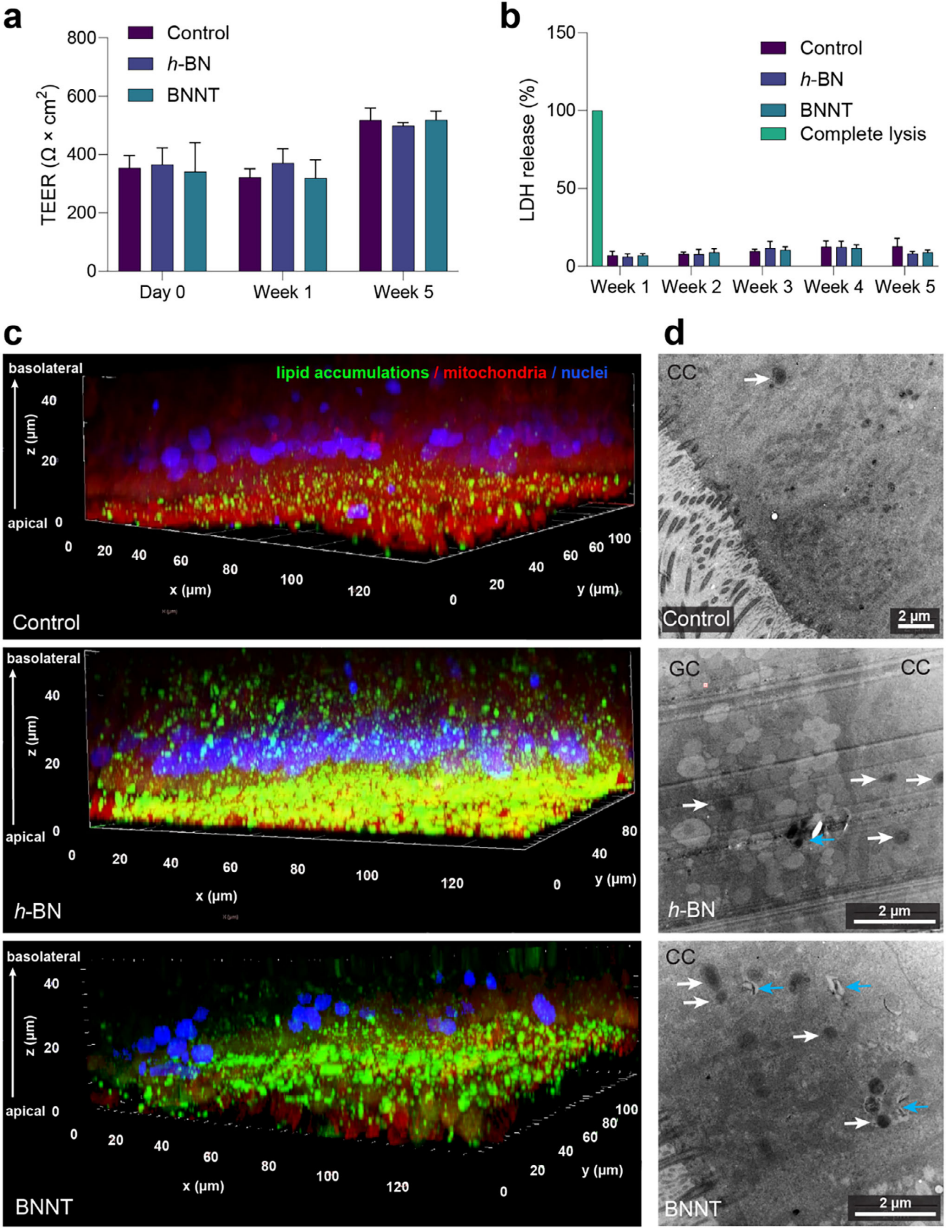
Cytotoxicity and lipid accumulation in healthy HBE cultures after repeated exposure to *h*‐BN and BNNTs for 5 weeks. a) No effects were observed in TEER or b) LDH release. c) Lipid accumulations were increased in *h*‐BN or BNNT‐exposed cultures as detected by fluorescence imaging of BODIPY (493/503) staining. The single‐channel fluorescence images, including zoomed‐in images showing cellular accumulation of lipid granules, can be found in Figure S3 (Supporting Information). d) TEM images demonstrate an accumulation of lipid droplets (white arrows) in goblet (GC) or ciliated cells (CC) with and without visibly internalized *h*‐BN‐ and or BNNT particles (blue arrows) after 5 weeks of exposure compared to the control. More representative micrographs can be found in Figure S4 (Supporting Information), showing lipid droplet accumulation in GC and CC with and without visibly internalized particles as well as an increased presence of mucus‐secreting vesicles in goblet cells after *h*‐BN and BNNT‐exposure. Data in graphs (a and b) are presented as mean + SD (*n* = 3). Statistical significance was calculated by applying One‐Way ANOVA and Dunnett`s post hoc test. *h*‐BN and BNNTs results presented in (a) and (b) were not statistically significant (*p* > 0.05) with respect to the control.

The loss of epithelial cells or damage in epithelial barrier integrity is typically associated with recurring infections, allergic reactions, chronic inflammation, and subsequent pathogenesis.^[^ ^53^, ^54^ ^]^ Therefore, we measured whether exposure to *h*‐BN or BNNTs could cause an effect on the epithelial cell viability or barrier integrity. As shown in **Figure** **2**a,b, no significant (*p* > 0.05) loss was observed either in barrier integrity (TEER values) or cell viability (based on LDH release) as compared to the control (exposed to vehicle) up to 5 weeks of repeated‐dose exposure to *h*‐BN or BNNTs. Also in TEM imaging, *h*‐BN‐ and BNNT‐exposed healthy HBE cell cultures showed an intact epithelial barrier (Figure S2, Supporting Information). The non‐cytotoxic effects of *h*‐BN in lung cell lines were also shown by other studies.^[^ ^12^, ^13^ ^]^ On the other hand, BNNT exposure has been shown to induce inflammatory responses in both macrophage‐like immune cells (differentiated THP1 cell line) and mouse lungs. Such effects were attributed to either the high aspect ratio of the materials or the presence of manufacturing impurities (i.e., toxic metals).^[^ ^14^, ^15^, ^16^, ^17^, ^18^ ^]^

Abnormal lipid deposition is a newly recognized pathology often reported during chronic respiratory illness.^[^ ^55^ ^]^ Clinical studies involving human volunteers with existing acute and chronic respiratory diseases (i.e., asthma, COPD, fibrosis, pneumonia, viral infections) have indeed shown an abnormal lipid deposition in the lungs of these patients and its association with inflammation, metabolic reprogramming, and disease progression.^[^ ^56^ ^]^ Therefore, we next investigated whether *h*‐BN or BNNT exposures could promote lipid deposition in our (multi‐cell type) model. Interestingly, we observed an increase in lipid accumulation after exposure to both *h*‐BN and BNNTs, as shown in the fluorescence images of healthy HBE cell cultures captured after neutral lipid‐staining with BODIPY (493/503) (Figure 2c; Figure S3, Supporting Information). TEM images of *h*‐BN and BNNT‐exposed healthy HBE cell cultures further confirmed a higher accumulation of lipid‐rich vesicles in exposed bronchial epithelial cells compared to untreated control (Figure 2d; Figure S4, Supporting Information).

**Figure 3:**
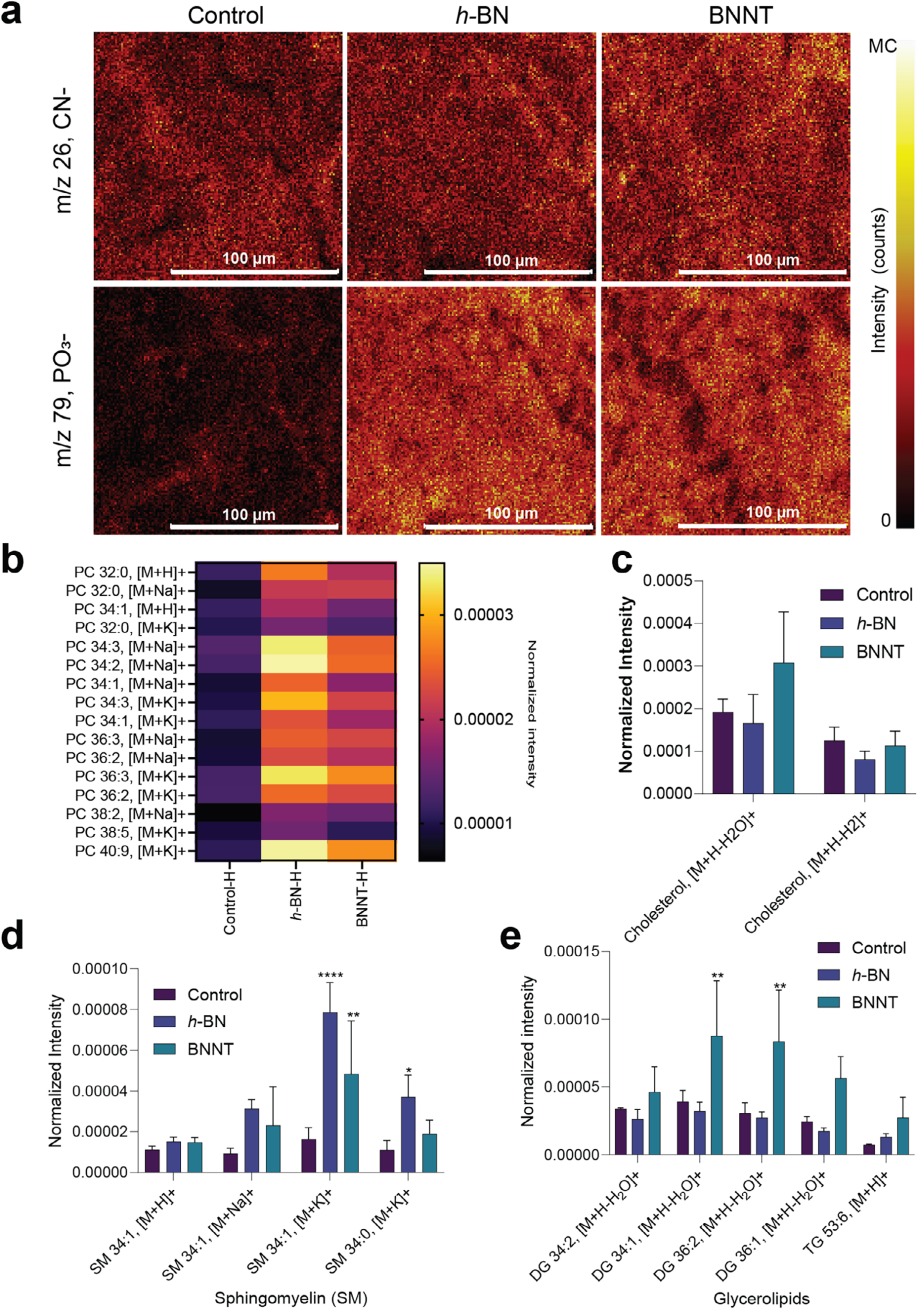
*h*‐BN and BNNT‐induced membrane lipid changes in healthy HBE cultures analyzed using ToF‐SIMS at positive ion mode. a) Reconstructed ToF‐SIMS images of HBE cultures showing the increase in overall protein (─CN) and phospholipid (─PO_3_) contents. Images were compressed to 128 × 128 pixels to increase the contrast. MC (maximum count) is 22 for CN^−^ and 32 for PO_3_^−^. b) Heat map showing normalized intensity values of different phosphatidylcholine (PC) species. c–e) The changes in (c) cholesterol, (d) sphingomyelin, and (e) diglycerides and triglycerides intensities in the cell membrane. The results in panels (c–e) are shown as mean + SD (*n* = 3). Statistical significance was calculated by applying One‐Way ANOVA and Tukey`s post hoc test. *p < 0.05; **p < 0.01; ****p < 0.0001.

Computational modeling experiments have shown that BNNTs could bind to lipid‐bilayer membranes and extract phospholipids and subsequently impact the ordering of lipids in the cell membrane.^[^ ^25^, ^26^, ^27^ ^]^ However, no studies have been performed using intact cells to show the specific effects of boron nanomaterials on cell membrane lipid composition and associated effects on cell signaling. To this end, we next applied time‐of‐flight secondary ion mass spectrometry (ToF‐SIMS)‐based untargeted surface lipidomic approach to uncover changes in plasma membrane lipids after exposure to *h*‐BN and BNNTs. A similar approach has been used previously by others to study lipidomic changes in cell membranes during nanoparticle exposure or diseases.^[^ ^28^, ^29^, ^30^, ^31^, ^32^, ^33^, ^34^, ^35^, ^36^, ^37^, ^38^, ^39^, ^40^, ^41^, ^42^, ^43^, ^44^, ^45^, ^46^, ^47^, ^48^, ^49^, ^50^, ^51^, ^52^, ^53^, ^54^, ^55^, ^56^, ^57^, ^58^ ^]^ Here, mass spectra of healthy HBE cell cultures were collected in both the positive and negative ion modes, allowing molecular recognition of abundant biomolecules in the cell membrane. As shown in **Figure** **3**, pseudo‐optical images of HBE cell cultures were reconstructed using the CN^–^ signal (m/z 26.00) to label protein‐rich structures, while the PO_3_^–^ signal (m/z 78.96) served as an indicator of total phospholipid content. The results indicated a relatively higher lipid content on the cell membrane based on PO_3_^–^ intensity upon exposure to *h*‐BN and BNNTs compared to the control (Figure 3a).

**Figure 4:**
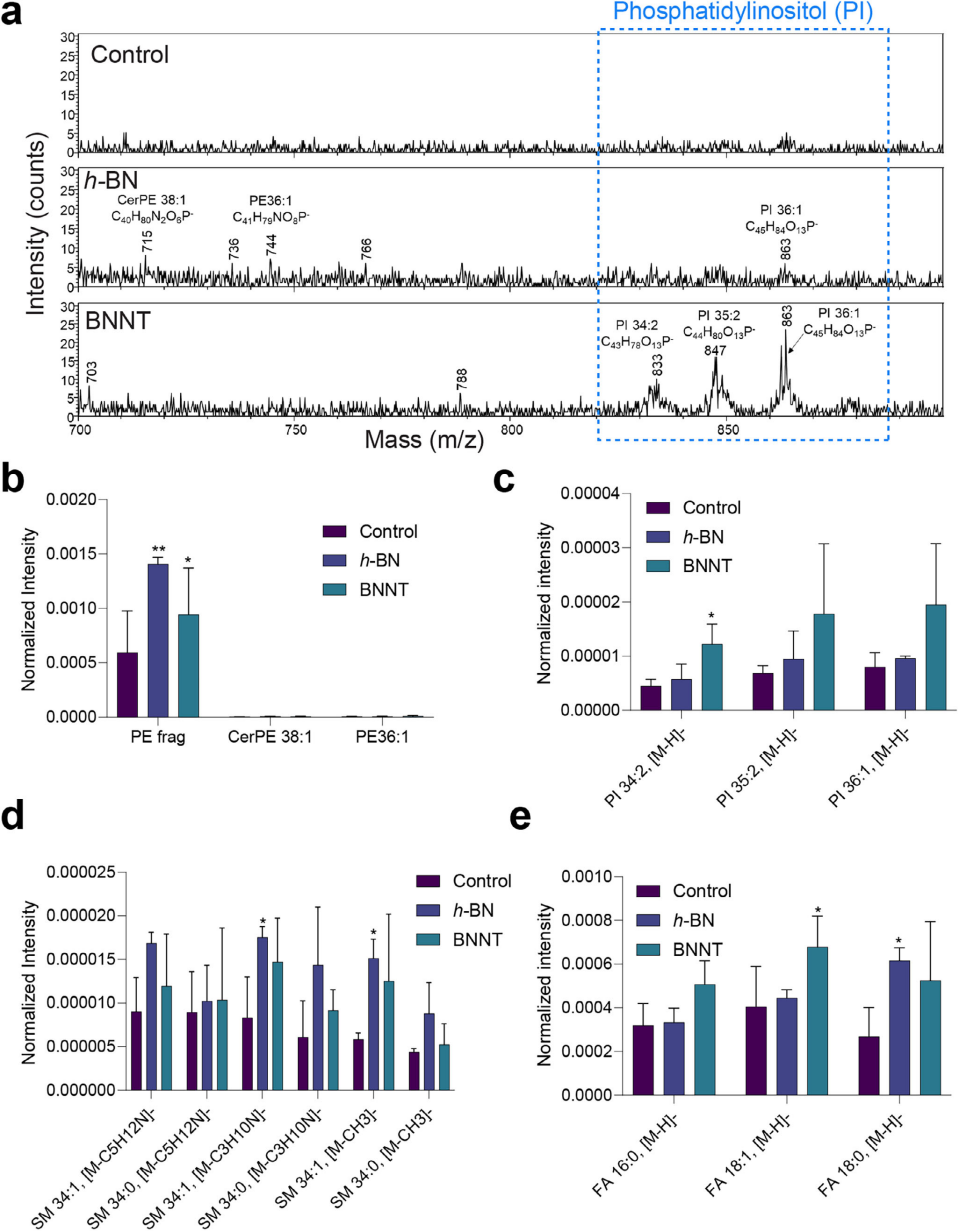
Lipid changes in the cell membrane of healthy HBE cultures detected in negative ion mode using ToF‐SIMS after exposure to *h*‐BN and BNNTs. a) Mass spectra indicating changes in phosphatidylethanolamine (PE) and phosphatidylinositol (PI) lipids. The region highlighted with dotted lines indicates specific changes in PI lipids after *h*‐BN and BNNT exposure. b–e) Changes in the intensities of (b) PE, (c) sphingomyelin (SM) lipids, and (d) fatty acids (FA). The results in panels (b–e) are shown as mean + SD (*n* = 3). Statistical significance was calculated by applying One‐Way ANOVA and Tukey`s post hoc test. *p < 0.05; **p < 0.01.

Going further, an in‐depth analysis of mass spectra of the healthy HBE cell cultures recorded in positive ion mode showed that phosphatidylcholine (PC), sphingomyelin (SM), and glycerides were the most affected lipids after exposure to *h*‐BN and BNNTs compared to the control (Figure S5, Supporting Information). The potential changes in the intensities of these specific lipid classes, including cholesterol, were further quantified and the results are presented in Figure 3. The intensities of PC head groups with polyunsaturated fatty acid tails (PC34:3; PC34:2, PC36:3, and PC40:9), and SM (SM34.1, SM34:0) were significantly (*p* < 0.05) elevated after exposure to *h*‐BN and BNNTs. However, no significant changes were observed for cholesterol (Figure 3b–d). In addition, BNNTs specifically triggered a significant (*p* < 0.05) increase in the intensities of di‐glycerides containing unsaturated acyl chains (DG 32:1, DG36:2) (Figure 3e). Furthermore, ToF‐SIMS spectra recorded in negative ion mode indicated major changes in the intensities of phosphatidyl‐ethanolamine (PE), phosphatidyl‐inositol (PI), and SM after exposure to *h*‐BN and BNNTs (**Figure** **4**a). Semi‐quantitative analysis of intensity revealed a significant (*p* < 0.05) increase in the contents of characteristic PE fragments (*m/z* 180.04, C_5_H_11_NO_4_P^−^) and SMs after both *h*‐BN and BNNTs exposure compared to the control (Figure 4b,c). Moreover, a specific increase in PI lipid was observed after exposure to BNNTs only (Figure 4d). The intensities of certain fatty acids (oleic acid (FA 18:1) and stearic acid (FA 18:0) were also enhanced significantly (*p* < 0.05) after exposure to either *h*‐BN or BNNTs as compared to the control (Figure 4e).

**Figure 5:**
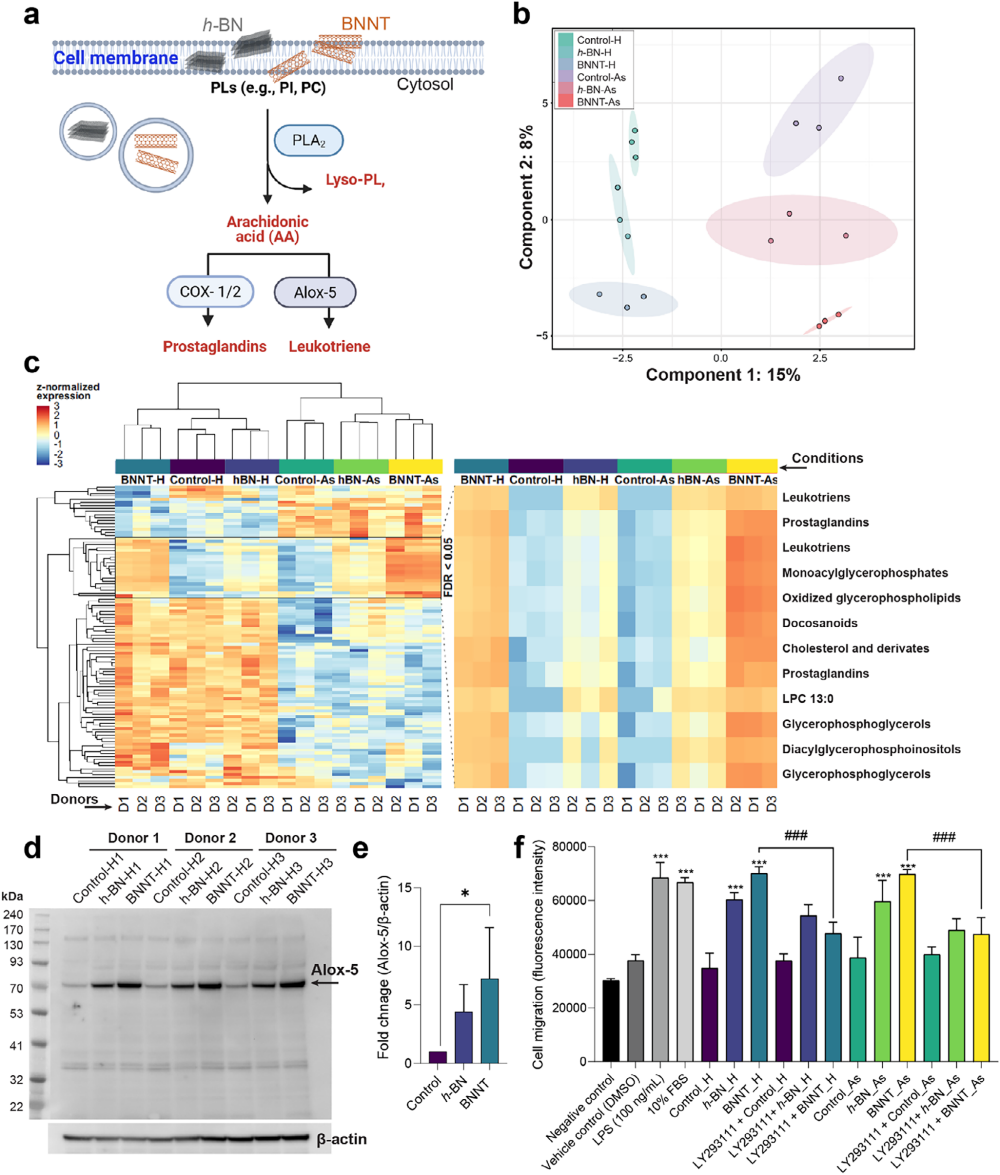
Global lipid profiling of healthy and asthmatic HBE cultures after exposure to *h*‐BN and BNNTs indicates effects on the production of eicosanoid lipid intermediates. a) Summary of cellular eicosanoid signaling involving enzymes to target cell membrane lipids (PLA_2_, phospholipase A2) and to further process resulting arachidonic acids intracellularly to produce prostaglandins (using COX1/2) or leukotrienes (5‐lipooxygenase, also known as Alox‐5). b) Scatter plot of the first two components of the supervised sparse Partial Least Squares ‐ Discriminant Analysis (sPLS‐DA) model of global lipidomic data in healthy and asthmatic samples with a 95% confidence interval. c) Hierarchical clustering showing the top 100 most significantly altered lipid expression values based on One‐way ANOVA, and the additional Heatmap on the right side further shows the annotation of lipids within the highlighted cluster with FDR values < 0.05. d) Immunoblot indicating expression of Alox‐5 (an enzyme that produces leukotriene B4) in healthy HBE cultures. β‐actin was used as a reference for loading uniformity. Refer to Data file S1 (Supporting Information) for full immunoblots. e) Densitometry analysis of the protein blot showing Alox‐5 expression with respect to β‐actin (loading control). f) diff‐HL60 cell migration after 24 h of exposure to conditioned medium from healthy (H) and asthmatic (As) HBE cell cultures in the presence or absence of leukotriene B4 receptor inhibitor (LY293111, 25 nm). Lipopolysaccharide (LPS, 100 ng mL^−1^) and fetal bovine serum (10%) were used as positive controls for chemotaxis‐mediated cell migration. The results in panels (e,f) are presented as mean + SD (*n* = 3). The statistical significance was calculated using One‐Way ANOVA analysis with Tukey`s post hoc test. *p = 0.06 and ***p < 0.001 ‐ statistical significance with respect to negative control. ^###^ *p* < 0.001 ‐ statistical significance between treatment groups (with and without inhibitor LY293111).

Taken together, our findings suggest that *h*‐BN and BNNTs are binding the cell membrane of lung cells and modulate their phospholipid content, in particular PC, PE, and PI. Interestingly, PC, PE, PI, and SM are the major building blocks for cell membranes and among these lipids, PC is the most abundant lipid^[^ ^59^ ^]^ that contributes to the maintenance of mechanical and biophysical properties of the cell membrane.^[^ ^60^ ^]^ Moreover, the disturbance of cell membrane lipid compositions could lead to adverse effects including subtle changes in membrane fluidity or receptor signaling. A recent study conducted a lipidomic analysis of healthy and asthmatic individuals and correlated changes in lipid composition to the severity of asthma.^[^ ^61^ ^]^ Their results showed a positive correlation between changes in PE, SM, and triglyceride (TG) amounts and the exacerbation of asthma. Disturbance of SM metabolism has also been shown to play a crucial role in inflammation‐mediated diseases,^[^ ^62^ ^]^ including the development of allergic lung pathology such as asthma.^[^ ^63^ ^]^ Finally, membrane‐bound di‐glycerides with unsaturated acyl chains, which are here altered upon BNNT treatment, serve as second messengers, as shown in other studies.^[^ ^64^ ^]^ Therefore, the abnormal cell membrane lipid composition especially the PC and PI lipid classes identified here, could promote intracellular inflammatory signaling via the production of lipid intermediates (i.e., leukotrienes, prostaglandins, di‐glycerides, or docosanoids). These lipid intermediates (also referred as lipid mediators) are the products of the eicosanoid lipid pathway, which involves the enzymatic (using phospholipase A2 or PLA_2_) release of unsaturated fatty acids from the cell membrane phospholipids that are further processed intracellularly to eventually produce prostaglandins or leukotrienes in the presence of COX‐1/2 or 5‐lipoxygenase (Alox‐5), respectively (also depicted in **Figure** **5**a).^[^ ^29^ ^]^

Since effects on lipid composition could have consequences in asthma development, we next compared the effects of *h*‐BN and BNNT exposures between healthy and asthmatic cell cultures (derived from healthy and asthmatic donors, respectively). To this end, healthy and asthmatic HBE cell cultures were repeatedly exposed to *h*‐BN and BNNTs for 5 weeks. First, the cytotoxicity of both materials in asthmatic cell cultures was analyzed by measuring LDH release (i.e., cell membrane integrity) and TEER (i.e., cell monolayer barrier integrity). No effects were observed either in LDH release or TEER, indicating the absence of direct cytotoxicity even after 5 weeks of exposure (Figure S6a,b, Supporting Information). However, we observed a lower TEER value in the asthmatic cell cultures (200–300 Ω × cm^2^, Figure S6a, Supporting Information) than in the healthy ones (300–400 Ω × cm^2^, Figure 2a), irrespective of any exposures. The observed lower TEER value in cultures of diseased cells could be due to effects on cell membrane ion channels (responsible for maintaining the electrochemical gradient), since it is a well‐known pathophysiological hallmark in bronchial asthma.^[^ ^65^, ^66^ ^]^ Next, global lipidomic profiling was performed to measure the overall change in cellular lipids, including a comparison between healthy and asthmatic groups. As shown in Figure 5b, the samples were separated according to their health and treatment status based on the first two components of the supervised sparse Partial Least Squares ‐ Discriminant Analysis (sPLS‐DA) model. This model highlighted 30 lipids with different quantitative values in healthy and asthmatic samples, as well as in *h*‐BN and BNNT‐treated and untreated samples. Next, the top 100 most significantly affected lipids in healthy and asthmatic cell cultures following exposure to cell medium (control) or *h*‐BN and BNNTs were plotted in a heatmap with hierarchical cluster analysis (Figure 5c). The most prominent and distinguishable effects in lipid composition between control and *h*‐BN‐ or BNNT‐exposed cells in both healthy and asthmatic groups were observed in one specific cluster (as highlighted in Figure 5c). This cluster included 12 significantly differentially expressed lipids (FDR < 0.05, plotted in the separate heatmap) that were further annotated using LIPID MAPS. Interestingly, the most affected lipid classes in the respective cluster were lipid intermediates, especially glycerolipids and eicosanoids (i.e., leukotrienes, prostaglandins, and docosanoids). In addition, the effects were more pronounced for BNNT exposure in asthmatic cell cultures than for healthy ones or *h*‐BN exposure. Mechanistically, the biosynthesis of eicosanoid lipids at the cellular level initiates from the cell membrane (as discussed above) and therefore, the effects of lipid intermediates upregulation achieved in global lipidomic analysis correlated well with the results obtained in ToF‐SIMS analysis showing altered lipid composition (e.g., upregulation of phospholipids) in the cell membrane. We also performed further statistical analysis by applying Tukey`s post‐hoc test on the lipid cluster showing the most downregulated signals in Figure 5c. Only relevant hits with p.adj < 0.05 from the comparisons between controls (healthy and asthmatic) and *h*‐BN or BNNT treatments (healthy and asthmatic) are presented in Table S1 (Supporting Information). We identified 13 out of 63 lipids showing statistically significant (p.adj < 0.05) differences between controls and treatments, of which 7 were down‐regulated (Table S1, Supporting Information). Again, the effects were more pronounced for BNNTs exposure than *h*‐BN, and the affected lipids were either fatty acids or phospholipids.

The biological functions of lipid intermediates, specifically of eicosanoid lipids, are well established in lung diseases: mediating immune cell infiltration and propagation of innate or adaptive immune responses during the pathogenesis of diseases. To verify the effect of *h*‐BN and BNNTs on the eicosanoid lipid biosynthesis pathway, we next performed immunoblotting for 5‐lipoxygenase (Alox‐5), an enzyme that transforms arachidonic acid (released from the cell membrane) into leukotriene. The results showed a higher expression of Alox‐5 after exposure to *h*‐BN and BNNTs with respect to the control in healthy HBE cell cultures, as evident in the immunoblot (Figure 5d) or densitometry plot of the corresponding blot (Figure 5e). Again, the effects in Alox‐5 expression were more pronounced for BNNT than *h*‐BN exposure, in agreement with the global lipidomic analysis. Next, we asked whether extracellular release of leukotrienes in conditioned medium from the HBE cell cultures would drive the recruitment of immune cells (i.e., neutrophils) by chemotaxis. To this end, we differentiated the HL‐60) cell line into neutrophil‐like cells (diff‐HL60, a widely accepted model for neutrophil research) using DMSO (1.25% for 5 days)^[^ ^67^ ^]^ and used them for a cell migration assay. The diff‐HL60 cells were stimulated using conditioned medium from healthy and asthmatic cultures exposed (or not) to BN nanomaterials for 24 h. The results showed a statistically significant increase in migration of diff‐HL60 cells when exposed to conditioned medium collected from either h‐BN or BNNTs cell cultures, irrespective of them being from healthy or asthmatic donors (Figure 5f). Again, the effects on neutrophil‐like cell migration were higher for BNNTs than h‐BN in both healthy and asthmatic conditions (Figure 5f). In addition, we demonstrated that inhibition of leukotriene B4 receptor (LTB4‐R) in diff‐HL‐60 cells using a pharmacological inhibitor, namely LY29311, before exposure to conditioned medium, significantly reduced the migration of diff‐HL‐60 in the case of conditioned medium from both healthy and asthmatic lung cells that have been exposed to BNNTs (Figure 5f). These results establish the role of LTB‐4 signaling in the potential of BNNTs‐exposed lung cultures to recruit immune cells as seen previously in lung tissues, whereby neutrophilic infiltration was reported upon BNNTs exposure.^[^ ^14^ ^]^

Previous studies have shown that graphene nanosheets and carbon nanotubes could modulate eicosanoid lipid synthesis.^[^ ^68^, ^69^, ^70^ ^]^ Lim et al. demonstrated that MWCNTs (Mitsui‐7) exposure of murine macrophages (J774A.1) promoted the expression of Alox5 mRNA and protein (1.0–1.5 fold increase in protein levels compared to control) along with an increased production and secretion of lipid intermediates (i.e., leukotriene B4 and prostaglandin E2), which drove the migration of neutrophil‐like cells (differentiated from HL‐60).^[^ ^70^ ^]^ More recently, Andrews et al. conducted the first crossover controlled exposure of healthy human volunteers to ultra‐small and small graphene oxide nanosheets (200 µg m^−3^ for 2 h) and their results demonstrated only very mild modifications of eicosanoid lipids in blood plasma with no overt detrimental effects in lung functions.^[^ ^68^ ^]^ In general, the unresolved occurrence of eicosanoid lipids in lung tissue could, in the long term, lead to immune activation and subsequent tissue damage that in turn may cause disease development.

### 2.3 Immunomodulatory Effects of *h*‐BN or BNNTs Mediated from Lipidomic Changes in Lung Tissue

**Figure 6:**
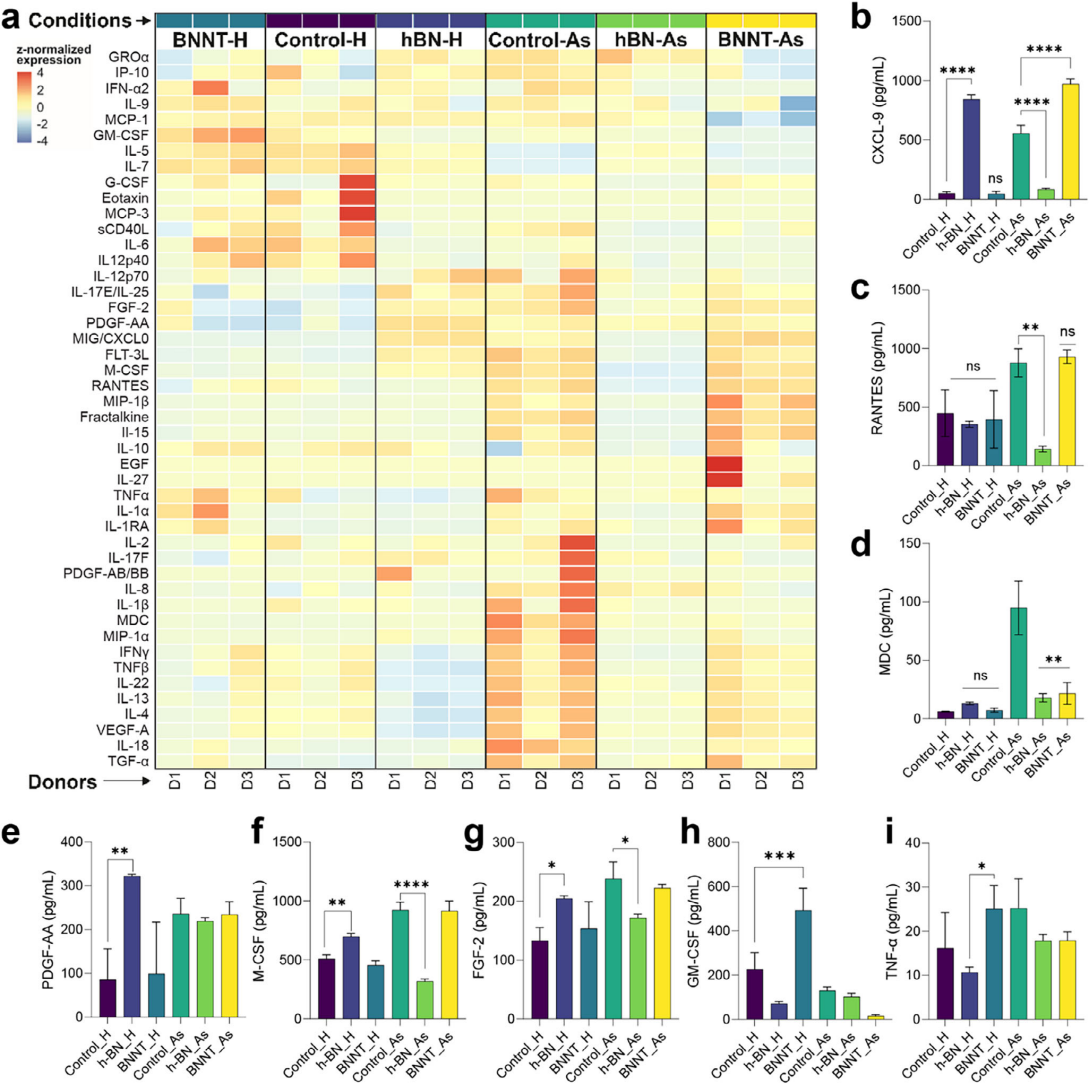
Cytokine‐chemokine release (48‐plex array) from healthy and asthmatic HBE cultures in the basolateral conditioned medium after 5 weeks of repetitive exposure to cell medium (control), *h*‐BN or BNNTs. a) Heatmap showing the release of different cytokines‐chemokines detected across different exposures in healthy (H) and asthmatic (As) cultures reconstituted from three donors (D1, D2, D3). Statistically significant effects were found in the release of b) CXCL9, c) RANTES, d) MDC, e) PDGF.AA, f) M‐CSF, g) FGF‐2, h) GM‐CSF, and i) TNF‐α. The data in (b–g) are presented as mean + SD (*n* = 3) and statistical significance was calculated by One‐Way ANOVA analysis with Tukey`s post hoc test. ^ns^ *p > 0.05*, *p < 0.05, **p < 0.01, ***p < 0.001, ****p < 0.0001.

The excess intracellular production and release of lipid intermediates in the broncho‐epithelial region could sensitize the bronchial airways by promoting inflammatory reactions either through the release of cytokines and chemokines from epithelial cells or via the recruitment of immune cells from peripheral blood. Therefore, we first investigated whether *h*‐BN or BNNTs exposure could directly induce the production of (pro)‐inflammatory or (pro)‐fibrotic cytokines/chemokines in either healthy or asthmatic HBE cell cultures, as previously shown by others for carbon‐based 2D materials,^[^ ^71^ ^]^ fibrogenic nanotubes,^[^ ^72^, ^73^, ^74^, ^75^ ^]^ and metal or metal oxide nanoparticles.^[^ ^76^, ^77^, ^78^ ^]^ To this end, multiplex cytokine array (48‐plex) measurements were performed using the conditioned medium (CM) collected from the basolateral compartment of our ALI cell culture model, following exposure to *h*‐BN or BNNTs, in either healthy or asthmatic lung cell cultures. **Figure** **6**a shows in a heat map the overall cytokines/chemokines release profiles for the different conditions tested, whilst Figure 6b–i are displaying all the statistically significant differences between these various conditions (*p* < 0.05). In healthy cell cultures, there was a significant increase in chemokine ligand 9 (CXCL9, also known as MIG), platelet‐derived growth factor AA (PDGF‐AA), macrophage colony‐stimulating factor (M‐CSF), and fibroblast growth factor 2 (FGF‐2) release for *h*‐BN exposed cells, whilst BNNTs increased the release of granulocyte‐macrophage colony‐stimulating factor (GM‐CSF). There was also a significant difference between *h*‐BN and BNNTs in tumor necrosis factor‐alpha (TNFα) release, albeit the difference was in both cases not statistically significant compared to the release in untreated healthy cells. In asthmatic cell cultures, there was a statistically significant decrease in CXCL9, the factor regulated on activation normal T‐cell expressed and secreted (RANTES; also known as CCL5), macrophage‐derived chemokine (MDC), FGF‐2, and M‐CSF‐2 release in *h*‐BN treated cells, whereas BNNTs increased CXCL9 release and inhibited MDC release. Taken together, these results demonstrate that each BN nanomaterial had their own material‐specific effect, and that this material‐specific effect was changing according to the health status of the cell cultures. For example, in healthy cell culture, *h*‐BN nanosheets increased not only CXCL‐9, but also PDGF‐AA, M‐CSF, and FGF‐2, whereas BNNTs had no effect on these immune modulators. Conversely, in asthmatic cell cultures that showed a higher secretion of CXCL‐9, PDGF‐AA, M‐CSF, and FGF‐2 compared to healthy controls, *h*‐BN reduced their release, whilst BNNTs had no effect or increased their secretion (i.e., CXCL9). For this increase in CXCL9 in asthmatic and not in healthy cell cultures after BNNTs exposure, one explanation could be a priming effect whereby the particles themselves are unable to activate the CXCL‐9 release pathway but can promote the release if the pathway is already activated. It is known that increased secretion of PDGF‐AA and CXCL9 from broncho‐epithelial cells is associated with lung pathogenesis and fibrosis development.^[^ ^79^, ^80^, ^81^ ^]^ Therefore, a chronic exposure to *h*‐BN could potentially be associated with the induction of fibrosis development based on the observed PGDF‐AA and CXCL9 results. In addition, GM‐CSF and TNF‐α are known to exhibit pro‐inflammatory properties, and their increased secretion from broncho‐epithelial cells can lead to airway remodeling and asthma pathogenesis exacerbation.^[^ ^82^, ^83^, ^84^, ^85^ ^]^

**Figure 7:**
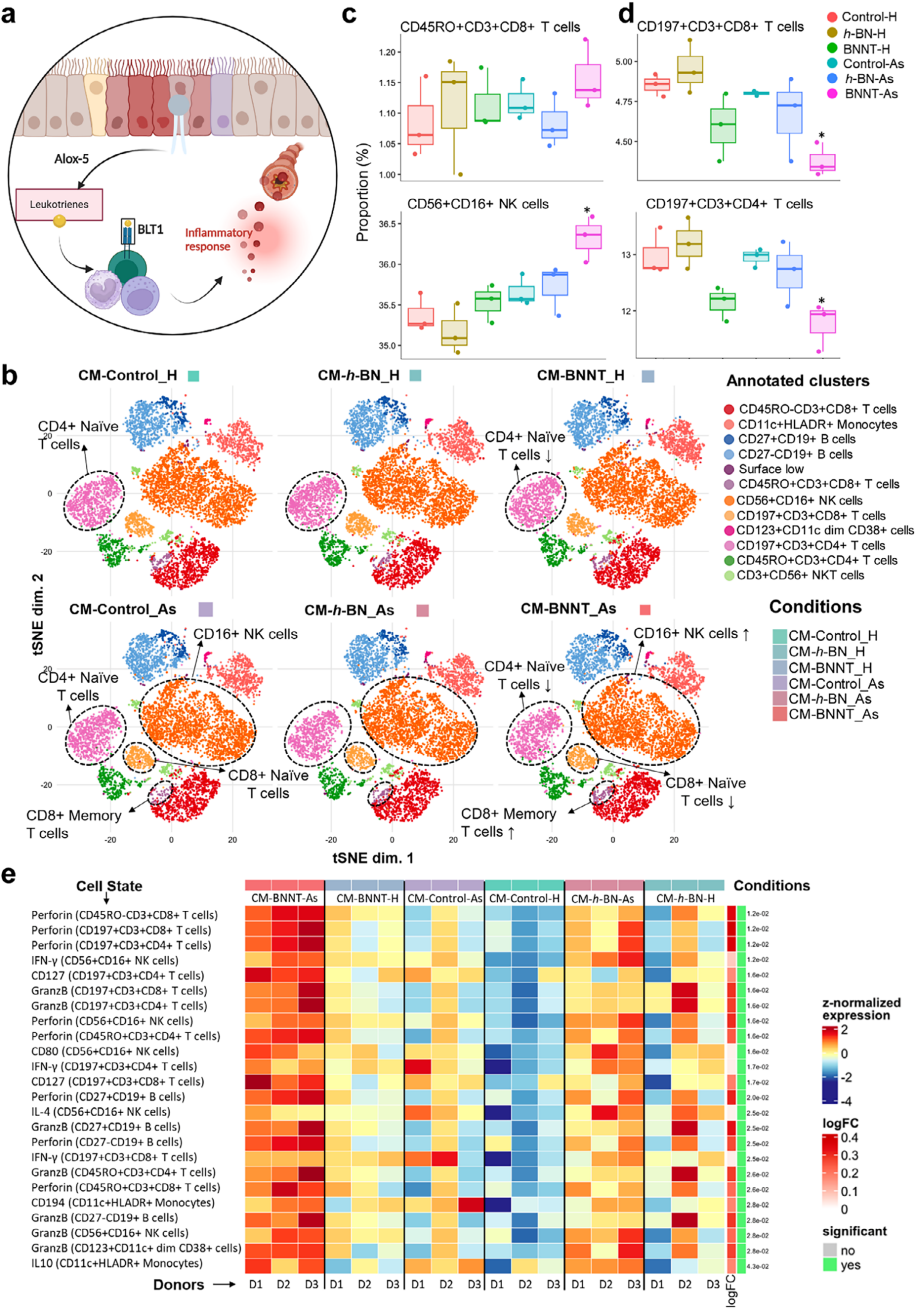
Single‐cell immune profiling of primary human PBMCs using mass cytometry (CyToF) after exposure (24 h) to conditioned media (CM) from *h‐*BN or BNNT‐treated healthy (H) and asthmatic (As) HBE cell cultures. a) Schematic representation of leukotriene–BLT1 lipid chemo‐attractant signaling and immune regulation in lungs: the extracellular release of leukotriene from lung epithelial cells could lead to the recruitment (by chemo‐attraction) and functional stimulation of BLT‐1 expressing immune cells from peripheral blood that subsequently sensitize airway tissues by releasing cytokine‐chemokine molecules and modulate disease progression. b) CyTOF results are represented in a T‐distributed stochastic neighbor embedding (tSNE) map with 12 clusters of cells identified in PBMCs with the most notable changes observed in NK cells, cytotoxic (CD8+) memory T cells, and naïve T cells (CD4+ and CD8+). c,d) Using the generalized linear model diffcyt‐DA‐EdgeR, we extracted the clusters that were significantly different between the conditions. Bar plots show the relative abundance of these clusters and that incubation of PBMCs with CM‐BNNT from asthmatic cultures triggered an increase in the number of NK cells and cytotoxic memory T cells (c), however, decreased the number of helper and cytotoxic naïve T cells (d). e) Heatmap showing median expression profiles of cell state‐specific markers across control and treatment groups for each donor using diffcyt‐DS‐limma (*n* = 3).

It was interesting and surprising to note that a limited number of cytokine/chemokine (out of 48) release was significantly impacted by the exposure to *h*‐BN or BNNTs when compared to the untreated control (Figure 6), although the biosynthesis of lipid intermediates was higher in asthmatic cell cultures after BNNT exposure (Figure 5c). This could be due to the already elevated production and release of various inflammatory cytokines/chemokines (i.e., high baseline levels) in control asthmatic cell cultures from different donors (referred as “asthmatic controls” in Figure 5a). Nevertheless, the increased production of lipid intermediates in asthmatic cell cultures could promote the immune response by recruitment of immune cells from peripheral blood via leukotriene–BLT1 lipid chemo‐attractant signaling (**Figure** **7**a). Therefore, we next investigated whether lipid intermediates (i.e., leukotrienes) are released from the broncho‐epithelial cells, activate immune cells by binding to the specific receptors (i.e., leukotriene B4 receptor, also known as BLT‐1), and subsequently modulate their functions in the airways (as depicted in Figure 7a). To verify this hypothesis, we isolated PBMCs from a healthy donor and incubated them with the conditioned medium (CM, containing secreted lipid intermediates from the cells) of either healthy or asthmatic HBE cell cultures that have been exposed to vehicle medium (untreated control), *h*‐BN or BNNTs (as depicted in Figure S7, Supporting Information). Following 24 h of incubation, the PBMCs were collected and analyzed using single‐cell mass cytometry (CyTOF) that allowed the segregation of different immune cell types into 12 clusters based on the expression of various cluster of differentiation (CD) markers (19 markers analyzed) and on their functional state using state markers (i.e., cytokines or chemokines, 17 markers analyzed) (Figure S7, Supporting Information). CyTOF is a powerful technique to characterize the heterogeneous immune cell population at a single‐cell level with high dimensionality, as previously demonstrated by others for understanding the direct immunomodulatory effects of 2D materials.^[^ ^86^ ^]^

To visualize exposure‐specific effects on cell types in the annotated clusters, we applied a 2D t‐SNE approach, whereby the most evident effects were observed in the adaptive immune cells (Figure 7b). An increase in the number of cytotoxic memory T lymphocytes (CD45RO+CD3+CD8+) and activated NK cells (CD16+CD56+) was recorded after incubation with CM from BNNT‐exposed asthmatic cell cultures (named CM‐BNNT‐As in Figure 7c). On the other hand, the number of naïve T cells (both CD8+ & CD4+) was reduced significantly after exposure to CM‐BNNT from asthmatic cell cultures, however, a trend was also observed in the case of healthy cell cultures (Figure 7d). The decrease in naïve T cells could result from the increased number of cytotoxic memory T cells, since naïve T cells could differentiate into different T cell subsets in the presence of a stimulus (i.e., disease or infections).^[^ ^87^ ^]^ The effects of CM‐*h*‐BN (healthy or asthmatic) on immune cells were either moderate or not significant. The expression of overall cell type‐specific markers from the 12 annotated clusters is also presented in a heatmap showing fold change in expression based on normalized frequency (Figure S8, Supporting Information). Furthermore, the analysis of functional (cell state) markers expression related to cytokine/chemokine levels (reflecting activation of immune cells during infection, pathogenesis or tissue damage) in immune cells revealed a significant increase in the release of granzyme B, perforin and INF‐γ, mainly from adaptive immune lymphoid cells (T cells, B cells, and NK cells) (Figure 7e). Interestingly, more potent immunomodulatory effects in PBMCs were observed after exposure to CM‐BNNT from asthmatic cell cultures than from healthy ones (Figure 7d). Among T cells, the expression of perforin, granzyme B and IFN‐γ was increased in T helper (CD4+) and cytotoxic T cells (CD8+), including memory (CD45RO+) T cells after exposure to CM‐BNNT (Figure 7d). In addition, IL10 and CCR4 (CD194) secretions were upregulated in monocytes (CD11c+HLADR+). Granzymes are a class of inflammatory mediators secreted by lymphoid cells, which have been shown to play a pivotal role in fatal asthma and its exacerbations.^[^ ^34^, ^35^, ^36^, ^37^ ^]^ In this context, Bratke et al. could show that challenging asthmatic patients (allergic origin) with an allergen via segmental allergen provocation (bronchial region) led to an enhanced influx of granzyme B ‐expressing lymphocytes (CD3+, CD8+, and CD16/56+) and higher extracellular granzyme B content in broncho‐alveolar lavage (BAL) fluid.^[^ ^88^ ^]^ Moreover, perforin‐expressing lymphocytes have also been shown to be elevated in the peripheral blood of patients with allergic as well as intrinsic asthma.^[^ ^35^ ^]^ A previous study by Lin et al.^[^ ^89^ ^]^ has demonstrated that *h*‐BN exposure in dendritic cells (DCs) triggered the release of anti‐inflammatory cytokines IL‐10 and IL‐12. In contrast, graphene oxide (GO) exposure induced the release of pro‐inflammatory cytokines such as TNF‐α, IL‐1β, and IL‐6 in DCs. Authors could also show *h*‐BN exposure in DCs and T‐cell co‐cultures model significantly increased IFN‐γ expression in T‐cells, which was also released extracellularly, whereas GO exposure in co‐cultures had no pronounced effects.

It is also interesting to note that B, T, and NK cells are known to strongly express the BLT1 receptor that is involved in leukotriene (LTB4)–BLT1 receptor‐mediated immune signaling, leading to the activation of lymphoid cells.^[^ ^90^, ^91^ ^]^ According to the Schmiedel dataset^[^ ^92^ ^]^ registered in the human protein atlas (https://www.proteinatlas.org/ENSG00000213903‐LTB4R/immune+cell), the BLT1 gene expression profile in peripheral immune cells is aligned in the following order: NK cells> classical monocytes > memory T cells > naïve T cells > B cells. As shown and discussed above in the lipidomics results (Figure 5c), BNNTs upregulated leukotriene levels in HBE cell cultures, which once released extracellularly, could specifically activate immune cells with elevated expression of the BLT‐1 receptor. Therefore, the selective activation of monocytes as well as T, B, and NK cells following exposure to CM‐BNNT in PBMCs (Figure 7e) may be explained by the induction of LTB4‐BLT1 immune signaling.

Since the immunomodulatory effects of CM‐BNNTs in PBMCs were more pronounced than those measured for CM‐*h*‐BN, this suggested a possible role of the aspect ratio of these two materials in inducing lipid‐mediated immunomodulatory effects. A recent study in mice, wherein oropharyngeal aspiration led to stronger and more sustained (up to 28 days) inflammatory response of BNNTs with fibrotic granulomatous lesions, has already proposed that the pulmonary toxicity of BNNTs when compared to *h*‐BN nanosheets may be aspect ratio dependent.^[^ ^14^ ^]^ Moreover, Horvath et al. observed that in vitro exposure of cells to BNNTs induced morphological alterations with multinucleated giant cell formation appearing in immune cells (macrophages).^[^ ^16^ ^]^ Other studies also described an acute inflammatory response of BNNTs in lung cells in vitro and recruitment of immune cells, including induction of pro‐inflammatory and fibrotic cytokine/chemokine factors in vivo in the BAL fluid of mouse lungs.^[^ ^17^, ^18^, ^19^, ^20^, ^21^, ^22^, ^23^, ^24^, ^25^, ^26^, ^27^, ^28^, ^29^, ^30^, ^31^, ^32^, ^33^, ^34^, ^35^, ^36^, ^37^, ^38^, ^39^, ^40^, ^41^, ^42^, ^43^, ^44^, ^45^, ^46^, ^47^, ^48^, ^49^, ^50^, ^51^, ^52^ ^]^ On the other hand, Lin et al. recently showed that *h*‐BN exposure promoted the maturation of human primary dendritic cells (DCs) enabling the release of pro‐inflammatory cytokines.^[^ ^89^ ^]^ Moreover, an increase in the proliferation of CD4+ T cells was reported after exposure to *h*‐BN sheets, irrespective of the presence or absence of DCs in the co‐cultured in vitro model. Overall, the long‐term biopersistence of BNNTs and BNNT‐induced immunomodulatory effects in the lungs could eventually lead to the onset (in healthy individuals) or progression (in individuals with pre‐condition, i.e., asthma) of respiratory diseases.^[^ ^93^ ^]^

To understand whether direct exposure of h‐BN and BNNTs in PBMCs could also trigger a similar immunomodulatory response, we next exposed PBMCs (isolated from one healthy donor) to 1 and 10 µg mL^−1^ of h‐BN and BNNTs for 24 h. The results showed, in contrast to the indirect exposure (conditioned medium from lung cultures), that a direct exposure of h‐BN and BNNTs at 10 µg mL^−1^ in PBMCs neither triggered an increase in CD56+CD16+ NK cells, nor a decrease of CD197+CD3+CD4+ T cells or CD197+CD3+CD8+ T cells (Figure S9, Supporting Information). However, an increase in CD45RO‐CD3+CD8+ T cells and CD45RO+CD3+CD4+ T cells was evident after exposure to h‐BN or BNNTs at 1 and 10 µg mL^−1^, indicating a differential response of PBMCs after direct exposure to nanomaterials, compared to indirect exposure via conditioned medium. In addition, no pronounced effects on functional markers after direct exposure of h‐BN and BNNTs with respect to the negative control were evident, except for an induction in MIP‐b expression in CD11c+HLDRA+ monocytes (Figure S10, Supporting Information).

### 2.4 BNNT Exposure in Mice Lungs Activates the Leukotriene Biosynthesis Pathway

The aspect ratio‐dependent effects of boron nanomaterials (*h*‐BN vs BNNTs) on lipid signaling were further validated in vivo in mouse lungs. Since leukotrienes were one of the most affected lipid intermediates in vitro in HBE cell cultures (see Figure 5), we focused on verifying the leukotriene biosynthesis pathway in mouse lungs. To this end, oropharyngeal aspiration (total 30 µL) of 0.5% BSA‐water (control mice), or *h‐*BN and BNNTs (1 µg µL^−1^, a total administered dose of 30 µg) were performed, and mice were sacrificed 28 days after exposure. The single high‐dose (30 µg) exposure selection was based on replicating a worst‐case scenario of occupational accidental exposure without personal protective equipment, as used by others.^[^ ^18^ ^]^ Previously published results involving some of the co‐authors from the present study demonstrated a significant accumulation of *h*‐BN and BNNTs in lung cells, 24 h after exposure.^[^ ^14^ ^]^ However, after 28 days of exposure, the remaining amount of BNNTs suggesting persistence, was higher than for *h*‐BN nanosheets, as evidenced by Raman analysis of tissue sections.

**Figure 8:**
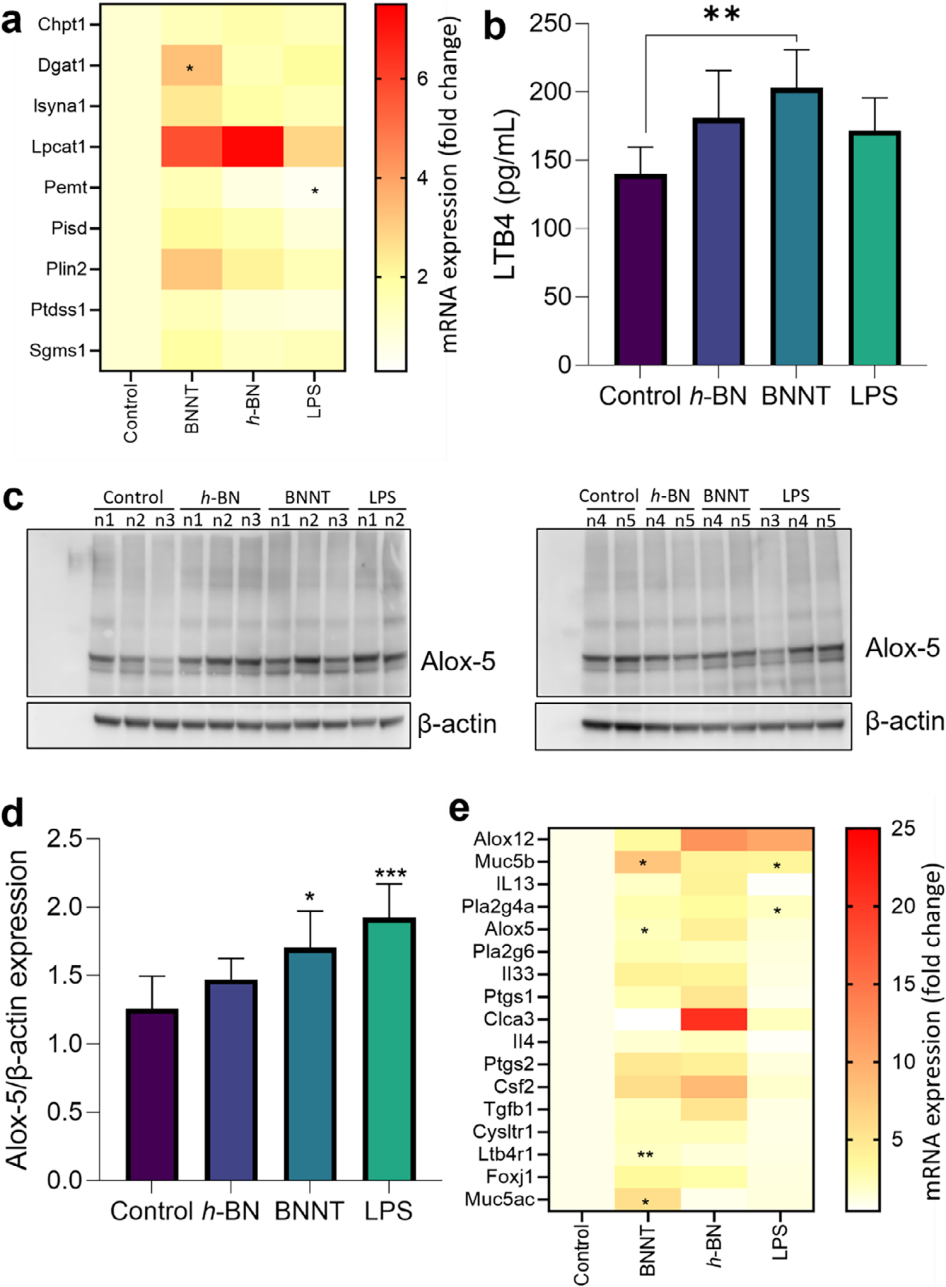
Leukotriene B4 biosynthesis pathway in mice lungs 28 days after pharyngeal aspiration to *h*‐BN and BNNT (1 µg µL^−1^; total administered volume 30 µL). a) Evaluation of mRNA transcripts in lung tissue for the genes involved in lipid biosynthesis and phospholipid remodeling. b) Leukotriene B4 content in mouse BAL fluid. c,d) Alox‐5 protein expression in lung tissues with respect to β‐actin (loading control), (b) protein blots, and (c) corresponding densitometry plot, showing Alox‐5 expression in five individual mice from control and *h*‐BN or BNNT exposures. The full protein blot for Alox‐5 and β‐actin can be found in Figure S11 (Supporting Information). e) Evaluation of mRNA transcript levels for the asthma marker genes in lung tissue using RT‐qPCR. The data in (b) and (d) are presented as mean + SD (*n* = 5) and statistical significance was calculated by One‐Way ANOVA analysis with Tukey`s post hoc test. **p < 0.01. The statistical analysis in (a) and (e) was performed by applying an unpaired *t*‐test to compare the fold change between the negative control and individual treatments, *n* = 5; (*) *p* < 0.05, (**) *p* < 0.01.

Taking this accumulation into consideration, we questioned whether exposure to *h*‐BN or BNNTs modulates membrane lipid biosynthesis. To this end, we performed gene expression analysis using RT‐qPCR for the enzymes involved in lipid metabolism and phospholipid biosynthesis. As shown in **Figure** **8**a, a significant increase in mRNA transcripts of Diacylglycerol O‐acyltransferase 1 (DGAT‐1) was observed in BNNT‐exposed mouse tissues, indicating a potential increase in the biosynthesis of triglycerides. Consequently, an increase in perilipin‐2 (PLIN‐2, a protein in lipid droplets) mRNA transcript was also observed in the BNNT‐treated group, suggesting potential storage of synthesized triglycerides (neutral lipids) in lipid droplets. For phospholipid biosynthesis, a notable increase in mRNA transcripts of the enzyme lysophosphatidylcholine acyltransferase 1 (LPCAT‐1) was observed in both h‐BN and BNNTs treated groups. LPCAT‐1 is a key enzyme in the phospholipid remodeling pathway that drives the synthesis of phosphatidylcholine, one of the most abundant phospholipids in the cell membrane. Overall, these results suggest a potential increase in lipid biosynthesis in mouse lung tissue 28 days after exposure to BNNTs.

Next, we investigated whether exposure to *h*‐BN or BNNTs could promote the release of leukotriene B4 (LTB4) in mouse bronchoalveolar lavage (BAL) fluid, since extracellular LTB4 is involved in immune cell recruitment as discussed above. As shown in Figure 8b, BNNT exposure led to a significant (*p* < 0.05) increase in the extracellular level of LTB4 in mouse BAL fluids. However, a slight increase in LTB4 was also seen after *h*‐BN exposure as compared to vehicle control mice. Next, we investigated the protein expression of Alox‐5, a key enzyme in the cellular biosynthesis of LTB4. A significantly higher expression of Alox‐5 was evident in BNNT‐exposed lung tissues (Figure 8c,d).

To further explore whether *h*‐BN or BNNTs exposure could induce asthma‐like characteristics in mice lung tissue, we evaluated mRNA transcript levels for the selected asthma biomarker genes (as in Table S3, Supporting Information) in mice lung tissue. As shown in Figure 8e, certain biomarker genes such as Alox‐5, Ltb4R1, MUC5B, and MUC5AC were significantly upregulated after exposure to BNNTs, potentially indicating tissue inflammation (driven by leukotrienes) and higher mucus production. In contrast, upregulation of these genes was not significant in h‐BN‐exposed lung tissue. Indeed, a previous study has already reported bronchial and pleural thickening of mouse lungs 28 days after exposure to BNNTs, but not *h*‐BN, using histopathological analysis.^[^ ^14^ ^]^ The thickening of bronchial airways, hypermucus secretion, and increased leukotriene production are well‐known biomarkers used in the pathological characterization of bronchial asthma.^[^ ^94^, ^95^, ^96^, ^97^ ^]^

Additional lung responses of these mice to *h*‐BN and BNNT exposure (i.e., immune influx, particle clearance, and histopathology) have been previously reported by some of us in a separate publication^[^ ^14^ ^]^ and a summary of the key findings is reproduced in Table S3 (Supporting Information). The results showed an induction of inflammatory cytokines (i.e., IFN‐γ, IL‐1a) and the influx of innate and adaptive immune cells in BAL fluids following exposure to BNNTs when compared to vehicle control. In addition, an increase in collagen deposition (indicative of fibrosis), together with bronchial airway thickening and an increase in granuloma size was described after BNNT exposure (Table S3, Supporting Information). Importantly, none of the above effects were found upon exposure of mice to *h*‐BN nanosheets (Table S3, Supporting Information). Similar immune responses (i.e., the influx of neutrophils, eosinophils and lymphocytes, as well as the induction of inflammatory cytokines in BAL fluids) were reported by Xin et al.^[^ ^52^ ^]^ in mouse lungs following acute and chronic exposures to unpurified BNNTs (40 µg) via pharyngeal aspiration. In addition, after 2 months of chronic exposure to unpurified BNNTs (40 µg), a higher accumulation of adaptive immune cells, mainly cytotoxic (CD8+) T cells and B cells, as well as an increase in extracellular IP‐10, KC, and IL‐6 cytokines, were found in the BAL fluid of these unpurified BNNT‐exposed mice.^[^ ^52^ ^]^

## 3 Conclusion

Collectively, our in vitro results using an advanced human broncho‐epithelial cell model (healthy and asthmatic) with repeated exposure to *h‐*BN or BNNTs (1 µg cm^−2^ twice per week) for 5 weeks showed effects on cellular lipid homeostasis with a profound increase in the biosynthesis of lipid intermediates (i.e., leukotrienes and prostaglandins) in asthmatic cell cultures upon BNNT exposure. In addition, *h‐*BN exposure also triggered the release of (pro)‐fibrotic cytokines (PGDF‐AA & CXCL9) in healthy cell cultures. While effects from *h*‐BN exposure were less pronounced and more moderate, it remains possible that the observed mild immunomodulatory effects could become relevant in case of co‐exposure to other immunomodulatory substances, while prolonging the exposure could lead to fibrotic tissue remodeling. The effects of h‐BN and BNNTs on cellular lipid homeostasis may induce metabolic stress and lipotoxicity in cells if they persist over the long term. On the other hand, lipophagy may play a protective role against boron nanomaterial‐induced lipotoxicity, as shown previously.^[^ ^13^ ^]^ Nevertheless, we further demonstrated that extracellular release of lipid intermediates from BNNT‐exposed asthmatic lung cell cultures led to the activation of lymphoid immune cell responses, as evidenced by single‐cell immune profiling of PBMCs following their exposure to conditioned medium from either healthy or asthmatic cell cultures exposed to *h*‐BN and BNNTs. Interestingly, an enrichment of lymphocytes (subsets of T cells, B cells, and NK cells) expressing granzyme B, perforin, and IFN‐γ was recorded when PBMCs were exposed to asthmatic CM‐BNNTs, which would likely contribute to the exacerbation of the disease (i.e., asthma) following exposure to BNNTs in a patient. We further demonstrate that conditioned medium from *h*‐BN and BNNTs‐exposed lung cultures (both healthy and asthmatic) induced migration of neutrophil‐like cells, which was driven by leukotriene B4 mediated chemotaxis, as pharmacological inhibition of the leukotriene B4 receptor in these cells inhibited their migration. In vitro findings were further corroborated in mice, 28 days after exposure to either *h*‐BN or BNNTs by pharyngeal aspiration. BNNTs promoted the LTB4 lipid biosynthesis in the lungs, which could provide a leading cause for the observed influx of immune cells in the lungs and the associated histopathological lesions reported previously.^[^ ^14^ ^]^ We also demonstrated that exposure to BNNTs in mice for 28 days triggered upregulation of some asthmatic disease biomarkers in lung tissue, suggesting the potential onset of disease. However, direct evidence showing LTB4‐mediated immune‐cell recruitment remains to be established in vivo. In addition, it also remains to be studied in the future whether mice with pre‐existing respiratory diseases, such as asthma, would respond differently to *h*‐BN or BNNT exposures. Further studies are also warranted to understand the neurotoxic effects of boron nanomaterials, since inhalation may allow particles to reach the brain. Overall, the results obtained through the combination of advanced 3D in vitro model of human origin and in vivo investigations are uncovering novel mechanisms involved in lung pathogenesis following exposure to boron nanomaterials under occupationally and therapeutically relevant conditions, and highlighting how people with a pre‐existing condition such as asthma might be prone to develop a more severe reaction upon exposure to high aspect ratio BNNTs materials. However, since the effects of *h*‐BN exposure in lung cells and mice were limited, the potential for its use in therapeutic applications remains promising. Nonetheless, further studies are needed to demonstrate its therapeutic efficacy and establish long‐term safety in respective biological models.

## 4 Experimental Section

### Nanomaterials Synthesis, Dispersion, and Characterization

The exfoliated *h*‐BN nanosheets were obtained (as powder) from BeDimensional SpA (Italy) within the Graphene Flagship consortium. Briefly, *h*‐BN nanosheets were synthesized by the liquid‐phase exfoliation method and then purified as described previously.^[^ ^14^, ^15^, ^16^, ^17^, ^18^, ^19^, ^20^, ^21^, ^22^, ^23^, ^24^, ^25^, ^26^, ^27^, ^28^, ^29^, ^30^, ^31^, ^32^, ^33^, ^34^, ^35^, ^36^, ^37^, ^38^, ^39^, ^40^, ^41^, ^42^, ^43^, ^44^, ^45^, ^46^, ^47^, ^48^, ^49^, ^50^, ^51^, ^52^, ^53^, ^54^, ^55^, ^56^, ^57^, ^58^, ^59^, ^60^, ^61^, ^62^, ^63^, ^64^, ^65^, ^66^, ^67^, ^68^, ^69^, ^70^, ^71^, ^72^, ^73^, ^74^, ^75^, ^76^, ^77^, ^78^, ^79^, ^80^, ^81^, ^82^, ^83^, ^84^, ^85^, ^86^, ^87^, ^88^, ^89^, ^90^, ^91^, ^92^, ^93^, ^94^, ^95^, ^96^, ^97^, ^98^ ^]^

BNNTs (refined puffball SP10RX) were synthesized by BNNT LLC (Newport News, VA) via the high‐temperature‐pressure method (HTP) as described elsewhere^[^ ^14^, ^15^, ^16^, ^17^, ^18^, ^19^, ^20^, ^21^, ^22^, ^23^, ^24^, ^25^, ^26^, ^27^, ^28^, ^29^, ^30^, ^31^, ^32^, ^33^, ^34^, ^35^, ^36^, ^37^, ^38^, ^39^, ^40^, ^41^, ^42^, ^43^, ^44^, ^45^, ^46^, ^47^, ^48^, ^49^, ^50^, ^51^, ^52^, ^53^, ^54^, ^55^, ^56^, ^57^, ^58^, ^59^, ^60^, ^61^, ^62^, ^63^, ^64^, ^65^, ^66^, ^67^, ^68^, ^69^, ^70^, ^71^, ^72^, ^73^, ^74^, ^75^, ^76^, ^77^, ^78^, ^79^, ^80^, ^81^, ^82^, ^83^, ^84^, ^85^, ^86^, ^87^, ^88^, ^89^, ^90^, ^91^, ^92^, ^93^, ^94^, ^95^, ^96^, ^97^, ^98^, ^99^ ^]^ and further purified to enrich the nanotubes content by removing non‐nanotube boron species as described in U.S. Patent US11629054B2, entitled “Boron Nitride Nanotube Purification”, published on 2023‐08‐18. Briefly, the as‐received BNNT material was first dispersed in absolute ethanol using tip and bath sonication along this process (a detailed procedure is provided in the method section in the supporting information). BNNTs were further washed using sterile Milli‐Q water and a stock suspension of 1 mg mL^−1^ was obtained to use in the experiments.

For the cell experiments, *h*‐BN nanosheets were weighed (≈2 mg) in a sterile glass vial and dispersed in BSA‐water (0.1%) via bath ultrasonication for 45 min in two intermittent cycles to achieve a 0.5 mg mL^−1^ stock suspension. Similarly, the BNNT stock suspension was diluted (1:1) in BSA‐water (0.2%), followed by bath ultrasonication for 45 min to achieve 0.5 mg mL^−1^ BNNT suspension in BSA‐water (0.1%). The albumin solution had been extensively used for the preparation of stable nanomaterial (especially nanotubes and 2D materials) suspensions for cell culture experiments.^[^ ^12^, ^100^ ^]^ The dispersed nanomaterials were further diluted (1 µg cm^−2^–16.5 µg mL^−1^) in a MucilAir^TM^ culture medium (obtained from Epithelix Sárl, Switzerland) for exposure to HBE cell cultures or in water for characterization. The following techniques were used to characterize the nanomaterials:

### Nanomaterials Synthesis, Dispersion, and Characterization—Transmission Electron Microscopy (TEM)

*h*‐BN and BNNTs were analyzed in TEM to determine the primary size and shape of the materials. The *h*‐BNs samples for TEM analysis were prepared by drop‐casting 5 µL of *h*‐BN and BNNT suspensions (50 µg mL^−1^) onto a 200‐mesh copper grid with holey carbon film (Electron Microscopy Resolutions, HC200Cu). The grids were air‐dried overnight at room temperature and imaged using a Zeiss EM 900 microscope at 80 kV (Carl Zeiss Microscopy GmbH, Germany).

For TEM analysis of BNNTs, samples were dispersed in ethanol by ultrasonic bath sonication for a few minutes. A few drops of the suspension were then deposited on a carbon‐coated TEM copper grid (Lacey Carbon 400 Mesh Cu). Samples were air‐dried and observed using a JEOL 1400 Orius TEM with an acceleration voltage of 120 kV. High‐resolution (HR) TEM images (used to produce the diameter and wall‐number distributions) were obtained using a JEOL JEM‐2100F TEM operated at 120 kV.

### Nanomaterials Synthesis, Dispersion, and Characterization—Raman Spectroscopy

For Raman spectroscopic analysis of *h*‐BN and BNNT suspensions, samples were prepared by drop casting the materials (16.5 µg mL^−1^) on a glass slide, followed by drying them overnight at room temperature. The next day, Raman analysis was performed using a WITec Alpha 300 RAS system (Oxford Instruments, Germany), and single‐point spectra were recorded using a 532 nm laser. Finally, the spectrum was processed using WITec Project Plus 6.1 software (Oxford Instruments, Germany), and the spectrum was presented after background and cosmic‐ray removal.

### Nanomaterials Synthesis, Dispersion, and Characterization—Lumulus Amebocyte Lysate (LAL) Test

The LAL test was performed to determine endotoxin contamination in *h*‐BN and BNNT suspensions (100 µg mL^−1^) using the Pierce LAL Chromogenic Endotoxin Quantitation kit (sensitivity 0.1 EU mL^−1^; Thermo Fisher Scientific, USA). Both nanomaterials were found endotoxin‐free since the levels were <0.5 EU/mL.

### Nanomaterials Synthesis, Dispersion, and Characterization—Zeta‐Sizer

The average hydrodynamic size and surface zeta (ζ)‐potential of *h*‐BN and BNNT suspensions (16.5 µg mL^−1^) in water and MucilAir^TM^ culture medium were determined using a Zetasizer Nano ZS instrument (Malvern Instruments, UK).

### Nanomaterials Synthesis, Dispersion, and Characterization—Inductively Coupled Plasma Mass Spectrometry (ICP‐MS)

The potential dissolution of *h‐*BN and BNNTs in MucilAir^TM^ culture medium compared to MilliQ water was determined using ICP‐MS. For detecting elemental boron (B) release in MucilAir^TM^ medium and water, *h*‐BN and BNNTs were freshly dispersed at 16.5 µg mL^−1^ (a stock suspension used for achieving 1 µg cm^−2^ exposure in HBE cultures) and incubated at 37 °C. Following incubation, 1 mL of the samples was withdrawn either immediately (0 h) or after 24, 48, and 72 h. After each time point, the samples were centrifuged at 20 000 g, for 45 min (4 °C). Following centrifugation, 0.5 mL of supernatant was carefully collected from the top and transferred into a sterile 2 mL microtube. The non‐centrifuged samples were also collected in parallel to determine the actual content of B (reference samples). Next, the samples were digested by adding 1 mL of nitric acid (HNO_3_, 65–67%, puriss. p.a. grade, Sigma Aldrich, Germany) mixed with 0.2 mL of hydrogen peroxide solution (H_2_O_2_, 30%, EMSURE, Supelco, Switzerland) and left at room temperature for at least 72 h to ensure complete mineralization. For reference (i.e., to prove complete mineralization within 72 h), samples with known *h*‐BN/BNNT mass were digested in a pressurized microwave (turboWAVE 1500 MWS GmbH, Germany) in Teflon tubes at 1000 W, 200 °C, and 150 bar pressure for 40 min. After digestion, all samples were diluted in ultrapure water to reach ≈2% of HNO_3_. B (^11^B isotope) content was measured using an Agilent 7900 ICP MS (Agilent, USA) instrument. The instrument was calibrated using a certified B reference standard (1000 mg L^−1^ B, MSB‐100PPM, Inorganic Ventures, Suisse Technology Partners, Switzerland) prepared at a range of concentrations (0.01, 0.5, 0.1, 1, 5, and 10 µg L^−1^ B) in 2% HNO_3_.

### MucilAir 3D Human Bronchial Epithelial Model

MucilAir^TM^, fully differentiated 3D human bronchial epithelial (HBE) cell cultures were obtained from Epithelix Sárl (Geneva, Switzerland). The primary cells were isolated from human biological samples under ethical approval and donor consent. Each batch of tissues was delivered with a certificate of analysis with donor information and quality control results. All the HBE cell cultures were tested negative against mycoplasma and HIV 1 & 2, Hepatitis B & C, SARS‐CoV‐2 viruses. HBE cell cultures were reconstituted using primary human upper airway cells from healthy or asthmatic donors and maintained in a humidified incubator (37 °C with 5% CO_2_) under an air‐liquid interface in a 24‐well transwell insert supplemented with MucilAir^TM^ cell culture medium (obtained from Epithelix Sárl, Geneva, Switzerland) at the basolateral compartment. The cell culture medium was renewed every 2–3 days, and apical washing was performed using buffered saline solution (1x PBS, Gibco) once a week to remove excess mucus deposition.

### *h‐*BN and BNNT Repeated‐Dose Exposure Setup Under a Semi‐Air‐Liquid Interface

Prior to starting the experiment, apical washing of HBE cell cultures was performed to remove the mucus layer. Next, the *h‐*BN and BNNTs were dispersed in the same way as described above and then diluted in MucilAir^TM^ culture medium to achieve 1 µg cm^−2^ (equal to 0.33 µg working concentration in 20 µL volume, which was then applied to the apical surface of the transwell cultures. Applying 20 µL of material suspension covers the whole apical surface of HBE cell cultures, which was absorbed by cells within 3 days. The exposure was repeated twice a week (every first and fourth day of the week) for five consecutive weeks (total applied dose was 10 µg cm^−2^ or 3.3 µg/insert) to achieve a dose more closely aligned with occupational relevance, as also applied by others for nanomaterials.^[^ ^21^, ^22^, ^23^, ^24^, ^25^, ^26^, ^27^, ^28^, ^29^, ^30^, ^31^, ^32^, ^33^, ^34^, ^35^, ^36^, ^37^, ^38^, ^39^, ^40^, ^41^, ^42^, ^43^, ^44^, ^45^, ^46^, ^47^, ^48^, ^49^, ^50^, ^51^, ^52^, ^53^, ^54^, ^55^, ^56^, ^57^, ^58^, ^59^, ^60^, ^61^, ^62^, ^63^, ^64^, ^65^, ^66^, ^67^, ^68^, ^69^, ^70^, ^71^, ^72^, ^73^, ^74^, ^75^, ^76^, ^77^, ^78^, ^79^, ^80^, ^81^, ^82^, ^83^, ^84^, ^85^, ^86^, ^87^, ^88^, ^89^, ^90^, ^91^, ^92^, ^93^, ^94^, ^95^, ^96^, ^97^, ^98^, ^99^, ^100^, ^101^ ^]^ During the exposure period, apical washing of mucus was performed every alternate week and before collecting the samples for analysis. The basolateral media were renewed whenever exposure was performed.

### Ciliary Interaction and Cellular Uptake of *h*‐BN and BNNTs—Scanning Electron Microscopy (SEM)

The interaction of *h*‐BN and BNNTs with cilia and the surface of HBE cell cultures was investigated using SEM. The samples for SEM analysis were processed by following the protocols suggested elsewhere.^[^ ^102^ ^]^ Briefly, HBE cell cultures were washed after 5 weeks of exposure using 1x PBS (Gibco), followed by fixing the cells using modified Karnovsky solution (4 g paraformaldehyde (Sigma–Aldrich, Germany), 50 mL Milli‐Q water, 5 mL glutaraldehyde 50% (Sigma–Aldrich, Germany) and 45 mL 1X PBS without glucose (Gibco), pH 7.4) for 1 h at RT. The HBE cell cultures in transwells were washed again and then processed for dehydration using a gradient series of ethanol (30 min 50%, 30 min 70%, 30 min 80%, 60 min 90% and 60 min 100% Ethanol at RT), followed by incubation with hexamethyldisilazane (HMDSO, 205389, Sigma–Aldrich, Germany) for 30 min at RT, similarly as described elsewhere.^[^ ^103^ ^]^ Samples were left overnight at RT for air‐drying and the next day, membranes holding HBE cell cultures were removed from the transwell insert holder using a scalpel and mounted onto SEM stubs. The samples were sputter‐coated with carbon (thickness 10 nm) using a high‐vacuum coater (Leica EM ACE 600, Switzerland). Images were acquired using an Axia ChemiSEM (Thermo Fisher Scientific) microscope at an accelerating voltage of 10 kV.

### Ciliary Interaction and Cellular Uptake of *h*‐BN and BNNTs—TEM

The cellular interactions and uptake of *h*‐BN and BNNTs, including potential ultrastructural changes in HBE cell cultures were investigated using TEM. HBE cell cultures were washed with sterile 1x PBS (Gibco) after 5 weeks of exposure and processed further by following previously published procedures.^[^ ^13^, ^102^ ^]^ Briefly, the HBE cell cultures in transwell membranes were prefixed using 3% glutaraldehyde (Sigma–Aldrich, Germany) solution prepared in 0.1 M Na‐cacodylate buffer (Electron Microscopy Sciences, U.S.A.) for 25 min at RT, followed by repetition with fresh fixative for 35 min at 4 °C. The samples were then washed twice for 20 min at 4 °C with 0.2 M Na‐cacodylate buffer (Electron Microscopy Sciences, U.S.A.) and kept in fresh 0.2 M Na‐cacodylate buffer until post‐fixation and staining with 2% OsO_4_ (Electron Microscopy Sciences) in 0.1 M Na‐cacodylate buffer for 30 min at 4 °C. The transwell membranes in inserts were then washed twice for 10 min at 4 °C with Milli‐Q water and serially dehydrated using an ethanol gradient (10 min 50%, 10 min 75%, 2x 15 min 100% from HoneyWell, Riedel‐de‐Haen, 3x 30 min 100% water‐free from Sigma–Aldrich) at 4 °C. Next, the transwell membranes holding HBE cell cultures were taken out from the inserts with a scalpel and shortly incubated in 100% acetone (Sigma–Aldrich, Germany) at RT inside a glass vial, followed by an Epon gradient in acetone (33% at 4 °C overnight, 66% at 4 °C for 6 h, 100% at RT for 2 h with the glass vial lid open for acetone evaporation) using Epon 812 substitute resin (Epoxy embedding kit 45 359, Sigma–Aldrich, Germany). Finally, the transwell membranes holding HBE cell cultures were cut into smaller pieces using scissors, embedded in molds using 100% Epon as above, and cured at 60 °C for at least 2 days. For TEM imaging, ultrathin sections ranging from 80 to 100 nm were prepared using an ultramicrotome (Leica EM UC6, Germany), placed onto Formvar‐coated copper grids (200‐mesh, EM Resolutions), post‐stained 10 min with uranyl acetate and 1 min with lead citrate at RT, and then imaged at different magnifications with a Zeiss EM 900 microscope (Carl Zeiss Microscopy GmbH, Germany) at 80 kV.

### Ciliary Interaction and Cellular Uptake of *h*‐BN and BNNTs—Confocal Raman Microscopy (CRM)

HBE cell cultures were exposed to *h*‐BN and BNNTs for 5 weeks as described above. After exposure, the mucus washing was performed, followed by fixation using 4% formaldehyde (Histofix^TM^, Sigma–Aldrich) for 20 min. The cultures were washed at least three times using 1x PBS (Gibco) and mounted on glass slides using Mowiol 4–88 (Sigma–Aldrich) and cured overnight at 37 °C. Next, Raman spectroscopy equipped with 532 and 488 nm lasers and coupled with confocal microscopy (WITec Alpha 300 RAS system, Oxford Instruments, Germany) was used to image surface interaction or potential cellular uptake of *h‐*BN and BNNTs. All the measurements were performed using a 532 nm laser (laser power applied: 10 mW) at 50x ZEISS LD EC Epiplan‐Neofuar Dic 50x/NA 0.55 objective. The Raman spectrum of *h*‐BN and BNNT materials alone was measured as a reference. In large‐area Raman mapping (100 × 100 cm), each image pixel was reconstructed from a specific Raman signature of materials (*h*‐BN or BNNT) and cellular components recorded in the spectrum. WITec Project Plus 6.1 software (Oxford Instruments, Germany) was used in data processing. The cosmic ray removal and baseline correction filters were applied in the data analysis.

### Ciliary Interaction and Cellular Uptake of *h*‐BN and BNNTs—ICP‐MS

The cellular boron (B) content in HBE cell cultures was measured after 5 weeks of exposure to *h‐*BN and BNNTs. Following exposure, the transwell membrane holding the HBE cell culture was collected and placed in a 2.0 mL microcentrifuge tube for further processing. 1 mL of nitric acid (HNO_3_, 65–67%, puriss. p.a. grade, Sigma Aldrich, Germany) mixed with 0.2 mL of hydrogen peroxide solution (H_2_O_2_, 30%, EMSURE, Supelco, Switzerland) was added in each tube and left for digestion at room temperature for at least 72 h. The instrument was calibrated using a certified B reference standard (1000 L^−1^ B, MSB‐100PPM, Inorganic Ventures, Suisse Technology Partners, Switzerland) prepared at a range of concentrations (0.01, 0.5, 0.1, 1, 5, and 10 µg L^−1^ B) in 2% HNO_3_. The isotope ^9^Be (100 µg L^−1^) was added and mixed online at a ratio of 1:10 with the standards and samples as an internal standard for correcting non‐spectral interferences. To ensure quality control in measurements, a known concentration of B (158 µg L^−1^) was measured in parallel with the samples (IV Stock 1643 Trace elements in water, Inorganic Ventures) and the recovery of the B was 98%. The results were presented as pg/mL B in ≈0.5 million cells that one transwell culture may contain (according to the information provided by the supplier).

### Cell Viability

To investigate loss of cell viability of healthy and asthmatic HBE cell cultures, lactate dehydrogenase (LDH) release in the basolateral medium was determined every week for 5 weeks during repetitive exposure to *h*‐BN and BNNTs. Triton‐X‐100 (0.2%, applied for 1 h) was used as a positive control for the LDH assay. The cell culture treated with vehicle medium (20 µL, culture medium supplemented with 0.1% bovine serum albumin) was used as a negative control. CytoTox96 non‐radioactive cytotoxicity kit (Promega, Germany) was used for the measurement of LDH release from cells. Briefly, 50 µL of basolateral supernatant was collected after exposure and added to a 96‐well plate and further mixed with 50 µL of LDH substrate provided with the assay kit. The plate was incubated for 30 min at RT before adding the stop solution and the absorbance was measured at 490 nm using a microplate reader (Mithras2 Plate reader, Berthold Technologies, Germany) as per manufacturer instructions. For data analysis, the obtained absorbance values were corrected with blank (culture medium only) and normalized to untreated controls. Results were presented as a percentage of LDH release compared to the highest LDH release due to cell lysis in the positive control.

### Epithelial Barrier Integrity

The effect on epithelial barrier integrity of healthy and asthmatic HBE cell cultures after exposure to *h*‐BN and BNNTs was determined by measuring the transepithelial electrical resistance (TEER) of the cell cultures before and after 1 or 5 weeks of exposure. At least three measurements were taken from different positions of the transwell inserts for each HBE cell culture. For data analysis, the recorded TEER values of each cell culture were first subtracted from the value obtained for the blank (cell‐free transwell insert) and then multiplied by the surface area of a transwell (0.33 cm^2^) to achieve actual TEER values (Ω × cm^2^) of the culture.

### Multiplex Cytokine Array

The boron nanomaterials induced cytokines/chemokines response from healthy and asthmatic HBE cell cultures was determined by conducting Luminex‐based 48‐Plex Discovery Assay (HD48A, Eve Technologies Corp, Calgary, Canada). The assay was composed of a range of immunomodulatory factors as listed here: sCD40L, EGF, Eotaxin, FGF‐2, Flt‐3 ligand, Fractalkine, G‐CSF, GM‐CSF, GROα, IFNα2, IFNγ, IL‐1α, IL‐1β, IL‐1ra, IL‐2, IL‐3, IL‐4, IL‐5, IL‐6, IL‐7, IL‐8, IL‐9, IL‐10, IL‐12p40, IL‐12p70, IL‐13, IL‐15, IL‐17A, IL‐17E/IL‐25, IL‐17F, IL‐18, IL‐22, IL‐27, IP‐10, MCP‐1, MCP‐3, M‐CSF, MDC (CCL22), MIG, MIP‐1α, MIP‐1β, PDGF‐AA, PDGF‐AB/BB, RANTES, TGFα, TNFα, TNFβ, VEGF‐A. For the measurements, the CM collected from the basolateral compartment of healthy and asthmatic HBE cell culture (number of donors = 3) after 5 weeks of exposure to *h*‐BN and BNNTs was first centrifuged (500 × *g*, 4 °C, 20 min), then passed via a micro‐column filter (pore size: 0.22 µm), and the flow‐through CM was frozen at −80 °C until analysis. The measurement of each sample was done in two technical replicates without dilutions. The aggregate mean value from two technical replicates was calculated and plotted for each donor and respective condition. The values falling below the standard curve were assigned a value of zero (i.e., IL‐3 and IL‐17A), and these were excluded from subsequent data analysis. The data was transformed into Z‐scores and plotted in a heatmap using the pheatmap library (version 1.0.12, Kolde, R. pheatmap: Pretty Heatmaps, 2022).

### Lipid Accumulation

The accumulation of neutral lipids in the cultures was determined by using BODIPY^493/503^ (Thermo Fisher Scientific) green‐fluorescent stain. In addition, the cell cultures were co‐stained with mitochondrial (MitoTracker red, Thermo Fisher Scientific) and nuclear stains (4′,6‐diamidino‐2‐phenylindole, DAPI, Sigma–Aldrich). Briefly, the healthy HBE cell cultures were first exposed to *h‐*BN and BNNTs for 5 weeks. Following exposure, the cell cultures were washed with 1x PBS at the apical side and then incubated with BODIPY^493/503^ (2.5 µm in cell medium) for 15 min at 37 °C. After incubation, the cell cultures were washed again and stained with MitoTracker red (250 nm in cell medium) and DAPI for 30 min and 10 min, respectively at 37 °C. In the end, the cell cultures were fixed using Histofix (Sigma–Aldrich) for 20 min, then washed thrice with PBS, and mounted on glass slides using Mowiol 4‐88 (Sigma–Aldrich). The samples were imaged using confocal laser scanning microscopy (CLSM; LSM780, Zeiss, Germany). The images were processed using Zeiss Zen software (Blue Edition version 3.9).

### Time‐of‐Flight Secondary Ion Mass Spectrometry (ToF‐SIMS)

Healthy and asthmatic HBE cell cultures were exposed to *h‐*BN and BNNTs for 5 weeks. Following exposure, the cell cultures were gently washed with 10 mm ammonium acetate and then immediately flash‐frozen in liquid nitrogen. The frozen cells were freeze‐dried in a lyophilizer and stored at −80 °C until use. Prior to ToF‐SIMS analysis, the transwell inserts with the cells were brought to room temperature and dried for 30 min in a desiccator under a low vacuum. The transwell membrane holding the cell cultures was cut off and fixed onto a metal disk using double‐sided conductive carbon tape. Next, ToF‐SIMS measurements were performed on a PHI nanoTOF II TOF‐SIMS instrument (Physical Electronics, Chanhassen, MN, USA) equipped with a 30 kV bismuth cluster liquid metal‐ion gun (LMIG). The Bi_3_^2+^ primary ion beam was operated in bunched mode with an unfiltered DC current of 11.3 nA. The secondary ions were mass separated in a triple focusing time of flight (TRIFT) analyzer before being detected by a dual microchannel plate detector. Charge compensation was achieved using a low‐energy electron gun (20 eV). Mass spectra and ion images were recorded in both positive and negative polarities, on areas of 150 × 150 µm with 256 × 256 pixels and an ion dose of ≈6.7 × 10^11^ ions cm^−2^. Three regions of interest were examined for each condition (on the same membrane).

Data processing was performed with the PHI TOF‐DR software (Physical Electronics, Chanhassen, MN, USA). Positive ion mass spectra were internally calibrated using commonly observed fragment peaks such as *m/z* 27.0235 C_2_H_3_^+^, *m/z* 29.0391 C_2_H_5_^+^, *m/z* 41.0391 C_3_H_5_^+^, and *m/z* 55.0547 C_4_H_7_^+^. Mass calibration was then refined by adding higher mass range peaks including *m/z* 184.0739 C_5_H_15_NPO_4_^+^ (phosphatidylcholine headgroup), *m/z* 369.3521 C_27_H_45_^+^ and *m/z* 385.3470 C_27_H_45_O^+^ (cholesterol fragments) to the calibration peak list. Negative ion mass spectra were calibrated using fragment peaks such as *m/z* 13.0078 CH^−^, *m/z* 25.0078 C_2_H^−^, *m/z* 48.0000 C_4_^−^, and *m/z* 49.0078 C_4_H^−^. Mass calibration was refined by adding common fatty acid peaks (*m/z* 255.2322 C_16_H_31_O_2_^−^, and *m/z* 281.2479 C_18_H_33_O_2_^−^) to the calibration list. Lipid ion peak intensities were normalized relative to the total ion intensity.

### Global Lipidomic Profiling and Data Analysis

Healthy and asthmatic HBE cell cultures were repeatedly exposed to *h*‐BN and BNNTs for 5 weeks as described above. For the lipid extraction, 0.5 mL methanol: butanol (1:1, HPLC grade, Sigma–Aldrich) was added to the basolateral side and 0.1 mL to the apical side of the transwells. The cell cultures were then incubated at room temperature for 15 min. Following incubation, the extracted lipids on the apical side were collected and transferred into a sterile microcentrifuge tube (PCR clean, Eppendorf). The extraction in the apical side of the cell culture was repeated by adding 0.1 mL of methanol:butanol (1:1) solution and incubating it for another 15 min. In the end, extracted lipids from both the apical and basolateral sides of the transwells were collected in the same tube and frozen immediately at −80 °C until use.

For liquid chromatography–mass spectrometry (LC‐MS) measurements, 500 µL from each sample was dried under a N_2_ environment and then reconstituted in 100 µL 50% methanol. 1 µL sample was injected in C18 column (Waters HSS T3) and run (flow rate 3.5–2.5 µL min^−1^) for 20 min at positive ion mode using Orbitrap LC‐MS (QExactive, Thermo Fisher Scientific). 5 mm NH4‐Acetate in water was used as mobile phase A, however mobile phase B was a mix of 5 mm NH4‐Acetate in 90% Isopropanol and 10% Acetonitrile (v/v). QCpool was made by pooling 20 µL of reconstituted samples. Initial data processing was performed using the MetaboAnalyst tool (https://www.metaboanalyst.ca/MetaboAnalyst/ModuleView.xhtml).

Further lipidomics data analysis was conducted in Python (version 3.8.8; Van Rossum, G. & Drake, F. L. Python 3 Reference Manual. CreateSpace, Scotts Valley, CA, 2009) and R (version 4.3.2; R Core Team. R: A Language and Environment for Statistical Computing. R Foundation for Statistical Computing, 2022). Initially, normalized abundance values were Log2 transformed. To tune the supervised sparse Partial Least Squares ‐ Discriminant Analysis (sPLS‐DA) model, implemented in the mixOmics library (version 6.26.0),^[^ ^104^ ^]^ and to determine the optimal number of variables to select for each component, a grid search was conducted using three‐fold cross‐validation and repeated 50 times. The sPLS‐DA model was then run using 15 variables for each of the two components. A scatter plot of the sample projections onto the first two components was generated, including 95% confidence ellipses for each group. One‐way ANOVA statistical testing was implemented to study the independent effect of health status and treatment. Following, the *p*‐values were corrected for multiple testing according to the Benjamini‐Hochberg (BH) method.^[^ ^105^ ^]^ Lipids with the 100 lowest FDR values were used for subsequent hierarchical clustering, based on the Euclidean distance of z‐scored row entries using the seaborn library (version 0.11.1; Waskom, M. L. seaborn: statistical data visualization. J. Open Source Softw. 6, 2021). A specific cluster was manually selected, and entries with an FDR < 0.05 were visualized as a heatmap. The annotation of specific lipid classes was performed using the LIPID MAPS® structure database (https://lipidmaps.org/resources/databases/index.php?tab=lmsd) as used and suggested by others.^[^ ^106^ ^]^ Briefly, the individual mass‐to‐charge ratios (m/z) were searched in the database, and the identified lipid classes were used for annotation.

### Chemotaxis Assay Using Neutrophil‐Like Cells

To investigate the impact of leukotrienes released in conditioned medium from *h*‐BN‐ and BNNT‐exposed healthy and asthmatic HBE cell cultures, a chemotaxis/cell migration assay was performed using neutrophil‐like cells (differentiated HL‐60). For the assay, the human (female) acute promyelocytic leukemia cell line HL‐60 (ATCC CCL‐240; Lot# 70051700; Date of purchase: 04 Feb 2025) was maintained in phenol red‐free RPMI‐1640 medium supplemented with 2 mm l‐glutamine and 10% heat‐inactivated FBS (Sigma). The cell line tested negative against mycoplasma before being used for experiments. The differentiation of HL‐60 cells into neutrophil‐like cells was performed by following the procedure described elsewhere.^[^ ^67^ ^]^ Briefly, the cells were seeded at 0.5 × 10^6^ cells mL^−1^ in the RPMI‐1640 complete medium supplemented with 1.25% DMSO and allowed to differentiate for 5 days in the incubator (5% CO_2_, 37 °C). The medium was changed after 3 days of differentiation and cells were kept again for another 2 days in freshly added differentiation medium.

The chemotaxis assay was performed using CytoSelect^TM^ 96‐well assay kit (pore size 3 µm; Cell Biolabs, USA) following the steps suggested by the manufacturer. Briefly, differentiated HL‐60 cells were thoroughly washed, re‐suspended in RPMI 1640 medium (phenol red‐free) without FBS (2.0 × 10^6^ cells mL^−1^) and left overnight for starvation in an incubator (5% CO_2_, 37 °C). The next day, the cell suspension was mixed briefly and 100 µL was added in the upper chamber of the transwell inserts (supplied with the kit). The lower chambers were filled either with RPMI‐1640 medium (without FBS, negative control or with FBS (10%, positive control) or LPS (100 ng mL^−1^, positive control). For the treatment, conditioned medium collected from healthy and asthmatic HBE cell cultures (after *h*‐BN or BNNTs exposure) was diluted 1:1 in serum‐free RPMI‐1640 medium and added to the lower chamber. For the inhibition assay, some cells were treated with the specific leukotriene B4 receptor inhibitor LY293111 (25 nM, Cayman Chemical) for 3 h to block LTB4‐mediated chemotaxis before the experiment. The plate was then incubated (5% CO_2_, 37 °C) for 24 h. Following incubation, the cells from the upper chamber were removed by aspirating, while the cells that migrated to the other side of the membrane were detached using 150 µL cell detachment solution. Next, 75 µL of detached cells in the respective solution and 75 µL of migrated cells in the lower chamber were combined in a new 96‐well plate and lysed. The cell lysates were incubated with CyQuant GR dye solution for 20 min at room temperature, and fluorescence was measured using a microplate reader (SYNERGY H1, BioTek). The data was presented as mean fluorescence intensity from three independent experiments (*n* = 3).

### Immune Cell Profiling Using Single‐Cell Mass Cytometry

Peripheral blood was obtained from a healthy adult donor under informed consent according to approval from the local cantonal ethics committee, St. Gallen, Switzerland (BASEC No. PB_2016‐00816). To isolate PBMCs from the peripheral blood, a density gradient centrifugation with Ficoll (Sigma–Aldrich) was done in SepMate™‐50 PBMC Isolation Tubes (StemCell Technologies). The isolated PBMCs were maintained in RPMI‐1640 medium supplemented with 10% heat‐inactivated FBS (Sigma), penicillin (100 U mL^−1^), and streptomycin (100 µg mL^−1^). Next, ≈1.25 × 10^6^ isolated PBMCs were distributed in each well of a 24‐well plate in 0.25 mL complete RPMI medium. For PBMC exposure, 0.25 mL of conditioned medium (CM; basolateral supernatant) was collected from healthy and asthmatic HBE cell cultures (three donors) after 5 weeks of exposure to *h*‐BN and BNNTs or vehicle medium only (vehicle control) and CM were stored at −80 °C freezer before use. For the exposure to PBMCs, the CM was centrifuged (20 000 ×g for 20 min at 4 °C) to remove any cell debris or NMs that may have translocated and then added in the respective wells of PBMCs and incubated for 24 h at 37 °C and 5% CO_2_. PBMCs supplemented with a conditioned medium of vehicle controls were used as a control for CyTOF experiments. In addition, direct exposure of PBMCs (*n* = 1) to *h*‐BN and BNNTs at 1 and 10 µg mL^−1^ was also performed for 24 h. Four hours before the end of the treatment, cells were incubated with 10 µg mL^−1^ of Brefeldin A (Invitrogen). Following the exposure, the cells were pelleted in a microcentrifuge tube by centrifugation at 500 × g for 5 min and stained with 198CisPt for 5 min at 37 °C, subsequently washed and resuspended in freezing medium (10% DMSO + 90% FBS) and stored in liquid N_2_ until further processing.

For single‐cell mass cytometry analysis, the frozen PBMCs were thawed, and cells were centrifuged at 450 × g for 5 min. The pelleted cells from each sample were subjected to mass‐tag cell barcoding (MCB) by using the Cell‐ID 20‐Plex Pd Barcoding Kit (Standard BioTools Inc., USA) by following the manufacturer's instructions. The barcoding was extended with combinations of CD45 mAbs tagged with 89Y, 115In and 209Bi to accommodate the second negative control. After barcoding, antibody labeling and staining were performed using the antibody panel shown in Table S4 (Supporting Information). The samples were washed (800 × g, 5 min) twice with 800 µL of cell staining buffer (CSB) and resuspended in 500 µL of CSB after final wash. Three‐quarters of each sample was combined into a single pool and centrifuged at 800 × g for 5 min. The complete pool was then stained with 450 µL of the surface marker cocktail in CSB for 30 min at RT. After staining, 12 mL of CSB was added in the cells and washed again (800 × g, 5 min). The cell pellet was resuspended, and intracellular staining was performed using a Foxp3 staining kit (Standard BioTools Inc., USA) and following the manufacturer's instructions. Briefly, the cells were supplemented with 600 µL CSB and 250 µL of the 4x fix reagent and incubated for 15 min at RT. After incubation, 14 mL of CSB was added and centrifuged at 800 × g for 5 min followed by washing twice in Foxp3 PERM buffer (800 × g, 5 min). After the final wash, the cells were resuspended in 100 µL of Foxp3 PERM buffer and 10 µL of cell suspension was aliquoted separately and used as the MMM (metal minus multiple) control. Next, 450 µL of intracellular mAb cocktail (5 µL each) was added to the remaining sample and gently resuspended followed by incubation for 30 min at RT. Both aliquots were then washed twice using CSB and incubated with Cell‐ID Intercalator‐Ir solution in 1.6% PFA for 20 min. Following incubation, the samples were washed twice using cell acquisition solution (CAS) and resuspended finally in CAS (1 million cells mL^−1^ with 1/10 EQ4 beads) and acquired on the CyTOF2 instrument (Standard BioTools Inc., USA). The resulting FCS files were normalized using instrument software. For debarcoding, an R! Script was used which allowed to separate all 20 constituents in one run. After measurement, dead cells from the analyzed data were excluded using FlowJo software. Next, the FCS files were generated and used to perform the R pipeline (R version 4.4.0). Moreover, CyTOF workflow pipeline and diffcyt methodologies were used in data analysis as described elsewhere.^[^ ^107^, ^108^ ^]^ CyTOF data were loaded using the R package FlowCore^[^ ^109^ ^]^ and Arcsinh transformation of the marker expression with a factor of 5 was applied. Unsupervised clustering was performed with FlowSOM and ConsensClusterPlus, which generated 20 meta‐clusters based on median marker expression values (CD3, CD4, CD8a, CD11c, CD14, CD27, CD16, CD56, CD161, HLA‐DR, TCRgd, CD45RA, CD45RO, CD19, CD38, sIgD, CD123, CD196, CD197).^[^ ^110^, ^111^ ^]^ After a merging step, 12 clusters (or cell populations) were separated and annotated based on the expression profiles of major cell types. The CATALYST package was used for dimension reduction (t‐stochastic neighbor embedding, t‐SNE) and visualization purposes. Differential cell proportion (differential abundance) and differential states were calculated using linear mixed models: diffcyt‐DA‐edgeR and diffcyt‐DS‐limma.^[^ ^107^, ^108^, ^109^, ^110^, ^111^, ^112^ ^]^ Statistical significance was defined as *p < 0.05 or adjusted *p* < 0.05 and an additional FDR threshold < 0.05 was applied.

### Animal Exposure and Sample Collection

A detailed methodology relating to animal exposure to *h*‐BN and BNNTs and tissue harvesting procedures is described in a previous report^[^ ^14^ ^]^ and samples used here come from the same study. All procedures were conducted after ethical approval from the UK Home Office, under Project License no. P089E2E0A. Briefly, C57BL/6J female mice (6 weeks old, purchased from Envigo, UK) were randomized for treatment and kept in ventilated cages in four groups. The animals were acclimatized for a week in a controlled environment (humidity, temperature, and light) and provided with ad libitum access to food and water. Next, the mice were administered via single pharyngeal aspiration to a suspension of 1 µg µL^−1^ of *h*‐BN and BNNTs (total 30 µg of materials prepared in 30 µL of BSA (0.5 %) in water for injection (v/v)) or controls (negative: vehicle 30 µL BSA‐water (0.5%), positive: Lipopolysaccharides (LPS) from Pseudomonas aeruginosa; Merck‐Sigma) 0.5 mg kg^−1^). The mice (*n* = 5) were then kept for 28 days after the single dose exposure. After 28 days, the mice were euthanized by IP injection of pentobarbitone and BAL fluids (*n* = 5) were collected by washing the right lung using ice‐cold PBS after clamping the left lung. Next, the right lungs (*n* = 5) were harvested and immediately frozen in liquid nitrogen and stored at −80 °C until further use.

Leukotriene content in BAL fluid was determined by using LTB4 ELISA (R&D Systems, #KGE006B) and performed by following the manufacturer's instructions. The BAL fluid that was collected from mouse lungs (*n* = 5) was centrifuged at 250 × *g* for 5 min (4 °C) and a cell‐free supernatant was used in the assay.

### mRNA Expression Analysis Using Real‐Time Quantitative Polymerase Chain Reaction (RT‐qPCR)

To understand whether exposure to *h*‐BN or BNNTs could modulate phospholipid biosynthesis or induce asthmatic responses in mice, mRNA expression analysis of biomarker genes (Table S3, Supporting Information, gene name and unique assay ID) was performed using a pre‐designed 96‐well SYBR Green primePCR assay plate (Biorad). RNA isolation from mouse lung tissue and cDNA preparation are described elsewhere.^[^ ^14^ ^]^ For qPCR reaction (Biorad), 50 ng of cDNA was added in predesigned 96‐well PrimePCR plates (Bio‐Rad) followed by adding SsoAdvanced Universal SYBR Green Supermix (Bio‐Rad) for a 20 µL reaction. For data analysis, the ΔΔCt values were calculated, normalized to GAPDH (housekeeping gene), and then expressed in fold change compared to the negative control (i.e., vehicle‐treated animals). The mean value (*n* = 5) was presented in a heat map.

### Western Blotting

The HBE cell cultures were lysed using RIPA buffer (Thermo Fisher Scientific), supplemented with protease and phosphatase inhibitors (Halt™ Protease Inhibitor Cocktail, Thermo Fisher Scientific; 1 mm PMSF, Sigma–Aldrich) and 1 mm DTT (Sigma–Aldrich). Next, culture lysates were centrifuged at 13 000 × g for 20 min, and supernatants were collected. For mouse lung tissues, the right lung samples were digested in 1 mL of RIPA buffer (Merck–Sigma Aldrich) supplemented with EDTA‐free protease inhibitor (Complete Mini, Roche) and homogenized (10 min at 50 Hz using stainless steel beads in a TissueLyser LT system (Qiagen). Lung tissue lysates were centrifuged for 5 min at 2600 g (Hettich, GmbH) at RT, and supernatants were stored at −80 °C until analysis. The protein concentration was measured using the Bradford assay kit (Thermo Fisher Scientific) by following the manufacturer's instructions. For PAGE, 15–20 µg of protein was loaded into each well of a NuPAGE 4–12% Bis‐Tris gradient gel (Thermo Fisher Scientific) and subjected to electrophoretic separation. The proteins were then transferred to a PVDF membrane (iBlot Gel transfer stacks, Thermo Fisher Scientific) using iBlot Gel transfer device (Thermo Fisher Scientific). The membrane was blocked for 1 h in 5% BSA (Sigma) and stained overnight at 4 °C with primary antibodies against ALOX‐5 (1:1000, Thermo Fisher Scientific, ARC1926). The membrane was reprobed with HRP‐conjugated β‐actin (1:5000, Thermo Fisher Scientific, MA5‐15739‐HRP) for 1 h at room temperature. The goat anti‐rabbit IgG (H+L) HRP‐conjugated antibody (Thermo Fisher Scientific) was used as a secondary antibody (1:10,000). The protein blot was imaged using ChemiDoc MP (BioRad) and densitometry analysis was performed using BioRad Image lab software version 6.1 (BioRad).

### Statistical Analysis

All the results were presented as “mean values ± standard deviation (SD)”. The error analysis of healthy and asthmatic HBE cell culture experiments was performed using measurements of cell cultures obtained from three independent human donors (*n* = 3). The error analysis of in vivo results was based on measurements from five independent mice (*n* = 5). Sample size *n*values are also provided for each experiment in the Figure legends. GraphPad Prism 9.0 software was used for data analysis. Statistical significance analysis of all experiments was performed by applying One‐(or Two, only in case of grouped data)‐way ANOVA followed by Dunnett's or Tukey's post hoc tests. *p* < 0.05 was considered statistically significant in all the experiments. The explicit *p*‐values assigned as statistically significant (*p < 0.05, **p < 0.01, ***p < 0.001, and ****p < 0.0001) compared to the negative control are provided in figure legends. BioRender.com was used for preparing Scheme 1 (only parts), Figures 5a and 7a and abstract art. R version 4.4.0 software was used in data analysis and plotting. The LIPID MAPS structure database was used for the annotation of lipid classes.

### Ethical Approval Statement

For HBE cell culture (MucilAir, Epithelix), experimental procedures were explained to the donors, and all subjects provided informed consent. The study was conducted according to the Declaration of Helsinki on biomedical research (Hong Kong amendment, 2013), and the research protocol was approved by the local ethics committee. All mouse in vivo experiments were conducted after ethical approval from the UK Home Office, under Project License no. P089E2E0A. Peripheral blood mononuclear cells were obtained from a healthy adult donor after informed consent, and the procedure was approved by the local cantonal ethics committee, St. Gallen, Switzerland (BASEC No. PB_2016‐00816).

## Conflict of Interest

The authors declare no conflict of interest.

## Author Contributions

J.B. and S.M.D. contributed equally to this work. G.G. designed and conceptualized the study, performed the cell‐based experiments, analyzed data, and wrote the paper; J.B. supervised by M.B. performed omics data analysis and curation; V.M.K. performed protocol development and sample preparation for TEM and SEM, TEM imaging, and TEM and SEM data analysis and data compilation of cells and nanomaterials; A.G. supported in ICP‐MS analysis and performed SEM imaging; V.A.N. helped in primary immune cell‐based experiments; S.M.D., C.S., and M.G. performed CyTOF measurements, data analysis and curation; T.F. performed TOF‐SIMS measurements and data analysis; E.F. developed and prepared BNNTs dispersions, and performed initial characterization; L.A.V.L. performed the animal exposure and collected samples with the help of C.B.; P.W. reviewed & edited the manuscript, and acquired funding; T.B. acquired funding, conceptualized and coordinated the study, and analyzed data, and contributed to writing the paper. All co‐authors contributed to the writing of the manuscript and approved the final version of the paper.

## Supporting information



Supporting Information

Supporting Information

Supporting Information

## Data Availability

The data that support the findings of this study are available from the corresponding author upon reasonable request.

## References

[1] Q. Cai , D. Scullion , W. Gan , A. Falin , S. Zhang , K. Watanabe , T. Taniguchi , Y. Chen , E. J. G. Santos , L. H. Li , Sci. Adv. 2019, 5, aav0129. 10.1126/sciadv.aav0129 PMC6555632 31187056

[2] J. G. Wang , F. C. Ma , W. J. Liang , M. T. Sun , Mater. Today Phys. 2017, 2, 6.

[3] D. Bae , K. H. Lee , M. J. Kim , Nanoscale 2024, 16, 3817. 38327235 10.1039/d3nr06070e

[4] R. F. Barth , M. G. Vicente , O. K. Harling , W. S. Kiger , K. J. Riley , P. J. Binns , F. M. Wagner , M. Suzuki , T. Aihara , I. Kato , S. Kawabata , Radiat. Oncol. 2012, 7, 146. 22929110 10.1186/1748-717X-7-146 PMC3583064

[5] Z. Yong , Z. Song , Y. Zhou , T. Liu , Z. Zhang , Y. Zhao , Y. Chen , C. Jin , X. Chen , J. Lu , R. Han , P. Li , X. Sun , G. Wang , G. Shi , S. Zhu , Chin. J. Cancer Res. 2016, 28, 634. 28174492 10.21147/j.issn.1000-9604.2016.06.10 PMC5242447

[6] V. A. Trivillin , A. Serrano , M. A. Garabalino , L. L. Colombo , E. C. Pozzi , A. M. Hughes , P. M. Curotto , S. I. Thorp , R. O. Farias , S. J. Gonzalez , S. Bortolussi , S. Altieri , M. E. Itoiz , R. F. Aromando , D. W. Nigg , A. E. Schwint , Int. J. Radiat. Biol. 2019, 95, 646. 30601686 10.1080/09553002.2019.1564080

[7] F. Heide , M. McDougall , C. Harder‐Viddal , R. Roshko , D. Davidson , J. Wu , C. Aprosoff , A. Moya‐Torres , F. Lin , J. Stetefeld , Sci. Rep. 2021, 11, 15520. 34330984 10.1038/s41598-021-95044-0 PMC8324832

[8] A. Hayat , M. Sohail , M. S. Hamdy , T. A. Taha , H. S. AlSalem , A. M. Alenad , M. A. Amin , R. Shah , A. Palamanit , J. Khan , W. I. Nawawi , S. K. Baburao mane , Surf. Interfaces 2022, 29, 101725.

[9] S. Roy , X. Zhang , A. B. Puthirath , A. Meiyazhagan , S. Bhattacharyya , M. M. Rahman , G. Babu , S. Susarla , S. K. Saju , M. K. Tran , L. M. Sassi , M. A. S. R. Saadi , J. Lai , O. Sahin , S. M. Sajadi , B. Dharmarajan , D. Salpekar , N. Chakingal , A. Baburaj , X. Shuai , A. Adumbumkulath , K. A. Miller , J. M. Gayle , A. Ajnsztajn , T. Prasankumar , V. V. J. Harikrishnan , V. Ojha , H. Kannan , A. Z. Khater , Z. Zhu , et al., Adv. Mater. 2021, 33, 2101589. 10.1002/adma.202101589 34561916

[10] V. Vatanpour , S. A. N. Mehrabani , B. Keskin , N. Arabi , B. Zeytuncu , I. Koyuncu , Ind. Eng. Chem. Res. 2021, 60, 13391.

[11] Z. X. Wu , J. L. Qi , W. B. Wang , Z. Y. Zeng , Q. Y. He , J. Mater. Chem. A 2021, 9, 18793.

[12] M. Carlin , J. Kaur , D. Z. Ciobanu , Z. Song , M. Olsson , T. Totu , G. Gupta , G. Peng , V. J. Gonzalez , I. Janica , V. Fuster Pozo , S. Chortarea , M. Buljan , T. Buerki‐Thurnherr , A. Esau Del Rio Castillo , S. B. Thorat , F. Bonaccorso , A. Tubaro , E. Vazquez , M. Prato , A. Armirotti , P. Wick , A. Bianco , B. Fadeel , M. Pelin , J. Hazard. Mater. 2024, 473, 134686. 38788582 10.1016/j.jhazmat.2024.134686

[13] G. Gupta , Z. Wang , V. M. Kissling , A. Gogos , P. Wick , T. Buerki‐Thurnherr , Small 2024, 20, 2308148. 10.1002/smll.202308148 38290809

[14] L. A. Visani de Luna , T. Loret , Y. He , M. Legnani , H. Lin , A. M. Galibert , A. Fordham , S. Holme , A. E. Del Rio Castillo , F. Bonaccorso , A. Bianco , E. Flahaut , K. Kostarelos , C. Bussy , ACS Nano 2023, 17, 24919. 38051272 10.1021/acsnano.3c06599 PMC10753895

[15] M. A. Lucherelli , X. Qian , P. Weston , M. Eredia , W. Zhu , P. Samori , H. Gao , A. Bianco , Adv. Mater. 2021, 33, 2103137. 10.1002/adma.202103137 34553436

[16] L. Horvath , A. Magrez , D. Golberg , C. Zhi , Y. Bando , R. Smajda , E. Horvath , L. Forro , B. Schwaller , ACS Nano 2011, 5, 3800. 21495683 10.1021/nn200139h

[17] V. K. Kodali , J. R. Roberts , M. Shoeb , M. G. Wolfarth , L. Bishop , T. Eye , M. Barger , K. A. Roach , S. Friend , D. Schwegler‐Berry , B. T. Chen , A. Stefaniak , K. C. Jordan , R. R. Whitney , D. W. Porter , A. D. Erdely , Nanotoxicology 2017, 11, 1040. 29094619 10.1080/17435390.2017.1390177

[18] V. Kodali , K. S. Kim , J. R. Roberts , L. Bowers , M. G. Wolfarth , J. Hubczak , X. Xin , T. Eye , S. Friend , A. B. Stefaniak , S. S. Leonard , M. Jakubinek , A. Erdely , Small 2022, 18, 2203259. 10.1002/smll.202203259 PMC9975644 36373669

[19] M. A. Soriano‐Ursúa , E. D. Farfán‐García , Y. López‐Cabrera , E. Querejeta , J. G. Trujillo‐Ferrara , Neurotoxicology 2014, 40, 8. 24189445 10.1016/j.neuro.2013.10.005

[20] S. Chortarea , H. Barosova , M. J. D. Clift , P. Wick , A. Petri‐Fink , B. Rothen‐Rutishauser , ACS Nano 2017, 11, 7615. 28505409 10.1021/acsnano.7b01992

[21] I. Kooter , M. Ilves , M. Grollers‐Mulderij , E. Duistermaat , P. C. Tromp , F. Kuper , P. Kinaret , K. Savolainen , D. Greco , P. Karisola , J. Ndika , H. Alenius , ACS Nano 2019, 13, 6932. 31188557 10.1021/acsnano.9b01823 PMC6750904

[22] S. Areecheewakul , A. Adamcakova‐Dodd , Z. R. Zacharias , X. Jing , D. K. Meyerholz , K. L. Legge , J. C. D. Houtman , P. T. O'Shaughnessy , P. S. Thorne , A. K. Salem , ACS Nano 2023, 17, 14586. 37463491 10.1021/acsnano.3c01668 PMC10416562

[23] M. R. Shurin , N. Yanamala , E. R. Kisin , A. V. Tkach , G. V. Shurin , A. R. Murray , H. D. Leonard , J. S. Reynolds , D. W. Gutkin , A. Star , B. Fadeel , K. Savolainen , V. E. Kagan , A. A. Shvedova , ACS Nano 2014, 8, 5585. 24847914 10.1021/nn406454u PMC4072415

[24] S. Beyeler , S. Chortarea , B. Rothen‐Rutishauser , A. Petri‐Fink , P. Wick , S. A. Tschanz , C. Garnier , F. Blank , Nanotoxicology 2018, 12, 699. 29804489 10.1080/17435390.2018.1472310

[25] Z. Li , Y. Zhang , C. Chan , C. Zhi , X. Cheng , J. Fan , ACS Nano 2018, 12, 2764. 29518314 10.1021/acsnano.7b09095

[26] Y. Zhang , C. Chan , Z. Li , J. Ma , Q. Meng , X. Cheng , J. Fan , Nanoscale 2018, 10, 14073. 29999094 10.1039/c8nr02018c

[27] Y. Zhang , Z. Li , C. Chan , J. Ma , C. Zhi , X. Cheng , J. Fan , Phys. Chem. Chem. Phys. 2018, 20, 3903. 29200219 10.1039/c7cp07136a

[28] S. P. Mukherjee , B. Lazzaretto , K. Hultenby , L. Newman , A. F. Rodrigues , N. Lozano , K. Kostarelos , P. Malmberg , B. Fadeel , Chem‐Us 2018, 4, 334.

[29] B. Samuelsson , S. E. Dahlen , J. A. Lindgren , C. A. Rouzer , C. N. Serhan , Science 1987, 237, 1171. 2820055 10.1126/science.2820055

[30] P. Montuschi , P. J. Barnes , J. Allergy Clin. Immunol. 2002, 109, 615. 11941309 10.1067/mai.2002.122461

[31] R. S. Peebles, Jr. , Pharmacol. Ther. 2019, 193, 1. 30081047 10.1016/j.pharmthera.2018.08.001 PMC6309751

[32] S. E. Wenzel , Prostaglandins Leukot Essent Fatty Acids 2003, 69, 145.12895597

[33] E. Schauberger , M. Peinhaupt , T. Cazares , A. W. Lindsley , Curr. Allergy Asthma Rep. 2016, 16, 48. 27333777 10.1007/s11882-016-0628-3 PMC5515624

[34] R. Annoni , L. F. Silva , Y. Nussbaumer‐Ochsner , A. van Schadewijk , T. Mauad , P. S. Hiemstra , K. F. Rabe , Eur. Respir. J. 2015, 45, 1485. 25745046 10.1183/09031936.00213814

[35] V. Arnold , S. Balkow , R. Staats , H. Matthys , W. Luttmann , J. C. Virchow, Jr. , Pneumologie 2000, 54, 468. 11203358 10.1055/s-2000-7690

[36] A. Hendel , P. R. Hiebert , W. A. Boivin , S. J. Williams , D. J. Granville , Cell Death Differ. 2010, 17, 596. 20139894 10.1038/cdd.2010.5

[37] C. M. Tschopp , N. Spiegl , S. Didichenko , W. Lutmann , P. Julius , J. C. Virchow , C. E. Hack , C. A. Dahinden , Blood 2006, 108, 2290. 16794249 10.1182/blood-2006-03-010348

[38] G. Varricchi , S. Ferri , J. Pepys , R. Poto , G. Spadaro , E. Nappi , G. Paoletti , J. C. Virchow , E. Heffler , W. G. Canonica , Allergy 2022, 77, 3538. 35950646 10.1111/all.15473 PMC10087445

[39] Z. Gu , H. L. Zuo , L. Li , A. H. Wu , Z. P. Xu , J. Mater. Chem. B 2015, 3, 3331. 32262327 10.1039/c5tb00248f

[40] S. Kittler , C. Greulich , J. S. Gebauer , J. Diendorf , L. Treuel , L. Ruiz , J. M. Gonzalez‐Calbet , M. Vallet‐Regi , R. Zellner , M. Köller , M. Epple , J. Mater. Chem. 2010, 20, 512.

[41] N. N. Mahmoud , R. Abu‐Dahab , M. Abdallah , S. Al‐Dabash , D. Abuarqoub , A. Albasha , E. A. Khalil , J. Drug Deliv. Sci. Technol. 2020, 60, 101965.

[42] T. L. Moore , L. Rodriguez‐Lorenzo , V. Hirsch , S. Balog , D. Urban , C. Jud , B. Rothen‐Rutishauser , M. Lattuada , A. Petri‐Fink , Chem. Soc. Rev. 2015, 44, 6287. 26056687 10.1039/c4cs00487f

[43] K. Daramy , P. Punnabhum , M. Hussain , C. Minelli , Y. W. Pei , N. J. W. Rattray , Y. Perrie , Z. Rattray , J. Pharm. Sci.‐Us 2024, 113, 2826. 10.1016/j.xphs.2023.12.021 38163549

[44] C. Gräfe , A. Weidner , M. von der Lühe , C. Bergemann , F. H. Schacher , J. H. Clement , S. Dutz , Int. J. Biochem. Cell B 2016, 75, 196. 10.1016/j.biocel.2015.11.005 26556312

[45] A. Lesniak , F. Fenaroli , M. P. Monopoli , C. Aberg , K. A. Dawson , A. Salvati , ACS Nano 2012, 6, 5845. 22721453 10.1021/nn300223w

[46] D. P. Gulo , N. T. Hung , W. L. Chen , S. H. Wang , M. Liu , E. I. Kauppinen , S. Maruyama , Y. M. Chang , R. Saito , H. L. Liu , J. Phys. Chem. Lett. 2023, 14, 10263. 37939010 10.1021/acs.jpclett.3c02528

[47] J. W. Seo , A. Pophali , S. W. An , C. S. L. Liang , S. H. Li , H. Y. Liu , J. Kim , K. J. An , J. Kim , T. Kim , J. Mol. Struct. 2025, 1319, 139545.

[48] J. Bourquin , A. Milosevic , D. Hauser , R. Lehner , F. Blank , A. Petri‐Fink , Adv. Mater. 2018, 30, 1704307. 10.1002/adma.201704307 29389049

[49] M. Geiser , W. G. Kreyling , Part. Fibre Toxicol. 2010, 7, 2. 20205860 10.1186/1743-8977-7-2 PMC2826283

[50] J. V. Fahy , B. F. Dickey , N. Engl. J. Med. 2010, 363, 2233. 21121836 10.1056/NEJMra0910061 PMC4048736

[51] M. Kucki , L. Diener , N. Bohmer , C. Hirsch , H. F. Krug , V. Palermo , P. Wick , J. Nanobiotechnology 2017, 15, 46. 28637475 10.1186/s12951-017-0280-7 PMC5480125

[52] X. Xin , M. Barger , K. A. Roach , L. Bowers , A. B. Stefaniak , V. Kodali , E. Glassford , K. L. Dunn , K. H. Dunn , M. Wolfarth , S. Friend , S. S. Leonard , M. Kashan , D. W. Porter , A. Erdely , J. R. Roberts , Nanoimpact 2020, 19, 100235.

[53] C. A. Akdis , Nat. Rev. Immunol. 2021, 21, 739. 33846604 10.1038/s41577-021-00538-7

[54] P. Losol , M. Sokolowska , Y. K. Hwang , I. Ogulur , Y. Mitamura , D. Yazici , Y. Pat , U. Radzikowska , S. Ardicli , J. E. Yoon , J.‐P. Choi , S.‐H. Kim , W. van de Veen , M. Akdis , Y.‐S. Chang , C. A. Akdis , Allergy Asthma Immunol. Res. 2023, 15, 705. 37957791 10.4168/aair.2023.15.6.705 PMC10643858

[55] D. M. Potashnikova , A. V. Tvorogova , A. A. Saidova , T. N. Sotnikova , E. A. Arifulin , T. V. Lipina , O. M. Shirokova , E. S. Melnikov , T. A. Rodina , A. A. Valyaeva , A. A. Zharikova , G. O. Zayratyants , O. V. Zayratyants , E. V. Sheval , L. B. Margolis , E. J. Vasilieva , bioRxiv 2023, 522299.

[56] A. Ravi , A. W. M. Goorsenberg , A. Dijkhuis , B. S. Dierdorp , T. Dekker , M. van Weeghel , Y. S. Sabogal Pineros , P. L. Shah , N. H. T. Ten Hacken , J. T. Annema , P. J. Sterk , F. M. Vaz , P. I. Bonta , R. Lutter , J. Allergy Clin. Immunol. 2021, 148, 1236. 33556463 10.1016/j.jaci.2020.12.653

[57] J. S. Fletcher , S. Samfors , J. Vallin , A. Svanstrom , J. Grantham , Anal. Bioanal. Chem. 2021, 413, 445. 33130974 10.1007/s00216-020-03013-9 PMC7806562

[58] G. Gupta , J. Kaur , K. Bhattacharya , B. J. Chambers , A. Gazzi , G. Furesi , M. Rauner , C. Fuoco , M. Orecchioni , L. G. Delogu , L. Haag , J. E. Stehr , A. Thomen , R. Bordes , P. Malmberg , G. A. Seisenbaeva , V. G. Kessler , M. Persson , B. Fadeel , ACS Nano 2023, 17, 17451. 37643371 10.1021/acsnano.3c05600 PMC10510732

[59] S. Furse , A. I. de Kroon , Mol. Membr. Biol. 2015, 32, 117. 26306852 10.3109/09687688.2015.1066894

[60] J. A. Szule , N. L. Fuller , R. P. Rand , Biophys. J. 2002, 83, 977. 12124279 10.1016/s0006-3495(02)75223-5 PMC1302201

[61] T. Jiang , L. Dai , P. Li , J. Zhao , X. Wang , L. An , M. Liu , S. Wu , Y. Wang , Y. Peng , D. Sun , C. Zheng , T. Wang , X. Wen , X. Cheng , Biochim. Biophys. Acta Mol. Cell Biol. Lipids 2021, 1866, 158853. 33160078 10.1016/j.bbalip.2020.158853

[62] M. Maceyka , S. Spiegel , Nature 2014, 510, 58. 24899305 10.1038/nature13475 PMC4320971

[63] K. Kowal , E. Zebrowska , A. Chabowski , Allergy Asthma Immunol. Res. 2019, 11, 330. 30912323 10.4168/aair.2019.11.3.330 PMC6439195

[64] M. Alwarawrah , F. Hussain , J. Huang , Biochim. Biophys. Acta 2016, 1858, 253. 26607007 10.1016/j.bbamem.2015.11.014

[65] I. A. D. Figueiredo , S. R. D. Ferreira , J. M. Fernandes , B. A. D. Silva , L. H. C. Vasconcelos , F. A. Cavalcante , Front. Pharmacol. 2023, 14, 1236550. 37841931 10.3389/fphar.2023.1236550 PMC10568497

[66] L. Yan , L. Zhang , K. Ogunniyi , L. Hong , J. Respir. Biol. Transl. Med. 2024, 1, 10019. 39664985 10.70322/jrbtm.2024.10019 PMC11633837

[67] S. Keshavan , G. Gupta , S. Martin , B. Fadeel , Nanotoxicology 2021, 15, 1125. 34657549 10.1080/17435390.2021.1988171

[68] J. P. M. Andrews , S. S. Joshi , E. Tzolos , M. B. Syed , H. Cuthbert , L. E. Crica , N. Lozano , E. Okwelogu , J. B. Raftis , L. Bruce , C. A. Poland , R. Duffin , P. H. B. Fokkens , A. J. F. Boere , D. L. A. C. Leseman , I. L. Megson , P. D. Whitfield , K. Ziegler , S. Tammireddy , M. Hadjidemetriou , C. Bussy , F. R. Cassee , D. E. Newby , K. Kostarelos , M. R. Miller , Nat. Nanotechnol. 2024, 19, 705. 38366225 10.1038/s41565-023-01572-3 PMC11106005

[69] S. Carvalho , M. Ferrini , L. Herritt , A. Holian , Z. Jaffar , K. Roberts , Front. Pharmacol. 2018, 9, 585. 29922162 10.3389/fphar.2018.00585 PMC5996183

[70] C. S. Lim , B. Veltri , M. Kashon , D. W. Porter , Q. Ma , Nanotoxicology 2023, 17, 249. 37115655 10.1080/17435390.2023.2204161 PMC10263170

[71] M. Pelin , C. Passerino , A. Rodriguez‐Garraus , M. Carlin , S. Sosa , S. Suhonen , G. Vales , B. Alonso , A. Zurutuza , J. Catalan , A. Tubaro , Nanomaterials 2023, 13, 2189. 37570507 10.3390/nano13152189 PMC10420834

[72] S. Hussain , S. Sangtian , S. M. Anderson , R. J. Snyder , J. D. Marshburn , A. B. Rice , J. C. Bonner , S. Garantziotis , Part. Fibre Toxicol. 2014, 11, 28. 24915862 10.1186/1743-8977-11-28 PMC4067690

[73] M. Polimeni , G. R. Gulino , E. Gazzano , J. Kopecka , A. Marucco , I. Fenoglio , F. Cesano , L. Campagnolo , A. Magrini , A. Pietroiusti , D. Ghigo , E. Aldieri , Part. Fibre Toxicol. 2016, 13, 27. 27251132 10.1186/s12989-016-0138-4 PMC4890337

[74] A. A. Shvedova , E. R. Kisin , R. Mercer , A. R. Murray , V. J. Johnson , A. I. Potapovich , Y. Y. Tyurina , O. Gorelik , S. Arepalli , D. Schwegler‐Berry , A. F. Hubbs , J. Antonini , D. E. Evans , B.‐K. Ku , D. Ramsey , A. Maynard , V. E. Kagan , V. Castranova , P. Baron , Am. J. Physiol. Lung Cell Mol. Physiol. 2005, 289, L698. 15951334 10.1152/ajplung.00084.2005

[75] G. Vietti , D. Lison , S. van den Brule , Part. Fibre Toxicol. 2016, 13, 11. 26926090 10.1186/s12989-016-0123-y PMC4772332

[76] A. R. Gliga , J. De Loma , S. Di Bucchianico , S. Skoglund , S. Keshavan , I. Odnevall Wallinder , H. L. Karlsson , B. Fadeel , Nanoscale Adv. 2020, 2, 648. 36133225 10.1039/c9na00721k PMC9417054

[77] J. McCarthy , I. Inkielewicz‐Stepniak , J. J. Corbalan , M. W. Radomski , Chem. Res. Toxicol. 2012, 25, 2227. 22931364 10.1021/tx3002884

[78] J. M. Veranth , E. G. Kaser , M. M. Veranth , M. Koch , G. S. Yost , Part. Fibre Toxicol. 2007, 4, 2. 17326846 10.1186/1743-8977-4-2 PMC1821039

[79] G. Kardas , A. Daszynska‐Kardas , M. Marynowski , O. Brzakalska , P. Kuna , M. Panek , Front. Pharmacol. 2020, 11, 47. 32116722 10.3389/fphar.2020.00047 PMC7033439

[80] N. Noskovicova , M. Petrek , O. Eickelberg , K. Heinzelmann , Am. J. Respir. Cell Mol. Biol. 2015, 52, 263. 25303647 10.1165/rcmb.2014-0294TR

[81] S. L. O'Beirne , S. M. Walsh , A. Fabre , C. Reviriego , J. C. Worrell , I. P. Counihan , R. V. Lumsden , J. Cramton‐Barnes , J. A. Belperio , S. C. Donnelly , D. Boylan , J. Marchal‐Sommé , R. Kane , M. P. Keane , J. Immunol. 2015, 195, 2788. 26268659 10.4049/jimmunol.1402008 PMC4777321

[82] M. A. Hardyman , E. Wilkinson , E. Martin , N. P. Jayasekera , C. Blume , E. J. Swindle , N. Gozzard , S. T. Holgate , P. H. Howarth , D. E. Davies , J. E. Collins , J. Allergy Clin. Immunol. 2013, 132, 665. 23632299 10.1016/j.jaci.2013.03.005

[83] S. Mattoli , M. Marini , A. Fasoli , Chest 1992, 101, 27S. 1311668 10.1378/chest.101.3_supplement.27s

[84] S. Saha , C. Doe , V. Mistry , S. Siddiqui , D. Parker , M. Sleeman , E. S. Cohen , C. E. Brightling , Thorax 2009, 64, 671. 19213775 10.1136/thx.2008.108290 PMC2712140

[85] Y. Yang , M. Jia , Y. Ou , I. M. Adcock , X. Yao , Clin. Respir. J. 2021, 15, 1027. 34097803 10.1111/crj.13407

[86] M. Orecchioni , D. Bedognetti , L. Newman , C. Fuoco , F. Spada , W. Hendrickx , F. M. Marincola , F. Sgarrella , A. F. Rodrigues , C. Menard‐Moyon , G. Cesareni , K. Kostarelos , A. Bianco , L. G. Delogu , Nat. Commun. 2017, 8, 1109. 29061960 10.1038/s41467-017-01015-3 PMC5653675

[87] L. Sun , Y. Su , A. Jiao , X. Wang , B. Zhang , Signal Transduct. Target Ther. 2023, 8, 235. 37332039 10.1038/s41392-023-01471-y PMC10277291

[88] K. Bratke , B. Bottcher , K. Leeder , S. Schmidt , M. Kupper , J. C. Virchow , W Luttmann , Clin. Exp. Immunol. 2004, 136, 542. 15147358 10.1111/j.1365-2249.2004.02468.x PMC1809040

[89] H. Lin , S. Peng , S. Guo , B. Ma , M. A. Lucherelli , C. Royer , S. Ippolito , P. Samori , A. Bianco , Small 2022, 18, 2107652. 10.1002/smll.202107652 35451183

[90] S. A. Islam , S. Y. Thomas , C. Hess , B. D. Medoff , T. K. Means , C. Brander , C. M. Lilly , A. M. Tager , A. D. Luster , Blood 2006, 107, 444. 16179368 10.1182/blood-2005-06-2362 PMC1490027

[91] J. Wei , K. Gronert , Trends Biochem. Sci. 2019, 44, 214. 30477730 10.1016/j.tibs.2018.10.007 PMC6379137

[92] B. J. Schmiedel , D. Singh , A. Madrigal , A. G. Valdovino‐Gonzalez , B. M. White , J. Zapardiel‐Gonzalo , B. Ha , G. Altay , J. A. Greenbaum , G. McVicker , G. Seumois , A. Rao , M. Kronenberg , B. Peters , P. Vijayanand , Cell 2018, 175, 1701. 30449622 10.1016/j.cell.2018.10.022 PMC6289654

[93] C. W. Agudelo , G. Samaha , I. Garcia‐Arcos , Lipids Health Dis. 2020, 19, 122. 32493486 10.1186/s12944-020-01278-8 PMC7268969

[94] A. Berger , BMJ 1999, 319, 90. 10398630 10.1136/bmj.319.7202.90 PMC1116241

[95] P. Bradding , C. Porsbjerg , A. Cote , S. E. Dahlen , T. S. Hallstrand , C. E. Brightling , J. Allergy Clin. Immunol. 2024, 153, 1181. 38395082 10.1016/j.jaci.2024.02.011

[96] C. M. Evans , K. Kim , M. J. Tuvim , B. F. Dickey , Curr. Opin. Pulm. Med. 2009, 15, 4. 19077699 10.1097/MCP.0b013e32831da8d3 PMC2709596

[97] C. Godson , N. Engl. J. Med. 2020, 382, 1472. 32268033 10.1056/NEJMcibr2000118

[98] A. E. Del Rio Castillo , V. Pellegrini , A. Ansaldo , F. Ricciardella , H. Sun , L. Marasco , J. Buha , Z. Dang , L. Gagliani , E. Lago , N. Curreli , S. Gentiluomo , F. Palazon , M. Prato , R. Oropesa‐Nuñez , P. S. Toth , E. Mantero , M. Crugliano , A. Gamucci , A. Tomadin , M. Polini , F. Bonaccorso , Mater. Horiz. 2018, 5, 890.

[99] M. W. Smith , K. C. Jordan , C. Park , J. W. Kim , P. T. Lillehei , R. Crooks , J. S. Harrison , Nanotechnology 2009, 20, 505604. 19907071 10.1088/0957-4484/20/50/505604

[100] M. J. Burgum , V. Alcolea‐Rodriguez , H. Saarelainen , R. Portela , J. J. Reinosa , J. F. Fernandez , V. I. Dumit , J. Catalan , F. C. Simeone , L. Faccani , M. J. D. Clift , S. J. Evans , M. A. Banares , S. H. Doak , NanoImpact 2025, 37, 100539. 39716585 10.1016/j.impact.2024.100539

[101] V. Kodali , J. R. Roberts , E. Glassford , R. Gill , S. Friend , K. L. Dunn , A. Erdely , J. Mater. Res. 2022, 37, 4620. 37193295 10.1557/s43578-022-00796-8 PMC10174278

[102] Z. Wang , J. Vernaz , N. Tagaras , B. Boda , T. Buerki‐Thurnherr , G. Reina , V. M. Kissling , S. Constant , G. Gupta , P. Wick , ACS Nano 2025, 19, 21426. 40474814 10.1021/acsnano.5c01981 PMC12177949

[103] A. D. Schoenenberger , J. Foolen , P. Moor , U. Silvan , J. G. Snedeker , Acta Biomater. 2018, 71, 306. 29530822 10.1016/j.actbio.2018.03.004

[104] F. Rohart , B. Gautier , A. Singh , K. A. Le Cao , PLoS Comput. Biol. 2017, 13, 1005752. 10.1371/journal.pcbi.1005752 PMC5687754 29099853

[105] Y. Benjamini , Y. Hochberg , J R Stat Soc B 1995, 57, 289.

[106] Z. Ni , M. Wolk , G. Jukes , K. Mendivelso Espinosa , R. Ahrends , L. Aimo , J. Alvarez‐Jarreta , S. Andrews , R. Andrews , A. Bridge , G. C. Clair , M. J. Conroy , E. Fahy , C. Gaud , L. Goracci , J. Hartler , N. Hoffmann , D. Kopczyinki , A. Korf , A. F. Lopez‐Clavijo , A. Malik , J. M. Ackerman , M. R. Molenaar , C. O'Donovan , T. Pluskal , A. Shevchenko , D. Slenter , G. Siuzdak , M. Kutmon , H. Tsugawa , et al., Nat. Methods 2023, 20, 193. 36543939 10.1038/s41592-022-01710-0 PMC10263382

[107] L. M. Weber , M. Nowicka , C. Soneson , M. D. d. Robinson , Commun. Biol. 2019, 2, 183. 31098416 10.1038/s42003-019-0415-5 PMC6517415

[108] M. Nowicka , C. Krieg , H. L. Crowell , L. M. Weber , F. J. Hartmann , S. Guglietta , B. Becher , M. P. Levesque , M. D. Robinson , F1000Res 2017, 6, 748. 28663787 10.12688/f1000research.11622.1 PMC5473464

[109] F. Hahne , N. LeMeur , R. R. Brinkman , B. Ellis , P. Haaland , D. Sarkar , J. Spidlen , E. Strain , R. Gentleman , BMC Bioinformatics 2009, 10, 106. 19358741 10.1186/1471-2105-10-106 PMC2684747

[110] S. Van Gassen , B. Callebaut , M. J. Van Helden , B. N. Lambrecht , P. Demeester , T. Dhaene , Y. Saeys , Cytometry A 2015, 87, 636. 25573116 10.1002/cyto.a.22625

[111] M. D. Wilkerson , D. N. Hayes , Bioinformatics 2010, 26, 1572. 20427518 10.1093/bioinformatics/btq170 PMC2881355

[112] M. D. Robinson , D. J. McCarthy , G. K. Smyth , Bioinformatics 2010, 26, 139. 19910308 10.1093/bioinformatics/btp616 PMC2796818

