# Supplementary material for: Boron Nitride Nanomaterials Trigger Immunomodulatory Effects in Human Broncho‐Epithelial Cells by Modulating Eicosanoid Lipid Signaling
Source: Adv Sci (Weinh). 2025 Dec 1;13(9):e16401. doi: 10.1002/advs.202516401 (PMC12904077; doi:10.1002/advs.202516401)
Supplement: Supplementary file 1 — Supporting Information [file ADVS-13-e16401-s001.docx]

Supplementary information

Boron Nitride Nanomaterials Trigger Immunomodulatory Effects in Human Broncho-Epithelial Cells by Modulating Eicosanoid Lipid Signaling

Govind Gupta^1*^, Jonas Bossart^1,2,3¥^, Sènan Mickael D'Almeida^4,5¥^, Luis Augusto Visani de Luna^6,7,8^, Christoph Schwärzler^4^, Tingting Fu^9^, Vanesa Ayala-Nunez ^1^, Alexander Gogos^1,10^, Marija Buljan^1,2^, Vera M Kissling^1^, Emmanuel Flahaut^11^, Miguel Garcia^4^, Cyrill Bussy^6, 7, 8^, Peter Wick^1^, Tina Buerki-Thurnherr^1*^

^1^Swiss Federal Laboratories for Materials Science and Technology (Empa), Laboratory for Particles-Biology Interactions, 9014 St. Gallen, Switzerland;  ^2^SIB, Swiss Institute of Bioinformatics, 1015 Lausanne, Switzerland; ^3^ETH Zurich, Department of Health Sciences and Technology (D-HEST), 8093 Zurich, Switzerland; ^4^Flow Cytometry Core Facility, School of Life Sciences, Ecole Polytechnique Fédérale de Lausanne (EPFL), Lausanne, 1015 Switzerland; ^5^Viollier AG, 4123 Allschwil, Switzerland; ^6^Centre for Nanotechnology in Medicine, School of Biological Sciences, Faculty of Biology Medicine and Health, The University of Manchester, Manchester M13 9PT, U.K.; ^7^National Graphene Institute, The University of Manchester, Manchester, M13 9PL, U.K.; ^8^Lydia Becker Institute of Immunology and Inflammation, Faculty of Biology, Medicine and Health, The University of Manchester, Manchester M13 9PT, U.K.; ^9^Department of Quantum Matter Physics, Laboratory of Advanced Technology, University of Geneva, 1211 Geneva 4, Switzerland; ^10^Nanoparticle Systems Engineering Laboratory, Institute of Process Engineering, Department of Mechanical and Process Engineering, ETH Zurich, Sonneggstrasse 3, 8092 Zurich, Switzerland; ^11^CIRIMAT, Université Toulouse 3 Paul Sabatier, Toulouse INP, CNRS, Université de Toulouse, 118 Route de Narbonne, 31062 Toulouse cedex 9, France

^¥^These authors contributed equally.

*Corresponding authors

Tina Buerki-Thurnherr

E-mail: tina.buerki@empa.ch

Orcid ID: 0000-0003-3723-6562

Govind Gupta

E-mail: govind.gupta@empa.ch

[Orcid ID:](https://orcid.org/0000-0003-3723-6562) 0000-0003-4703-418X

**Method**

**BNNT preparation:** All flasks/vials used, tweezers, spatulas and glass pipettes were washed with ethanol before each use to ensure disinfection. For each batch, 15 mg of dry BNNTs were weighed and placed in a glass vial. The commercial reference of the sample used is SP10 RX. Fifteen mL of absolute ethanol was added to the vial containing the 15 mg of BNNTs. The vial was closed and vigorously shaken manually. It was then processed using a small-diameter (3mm) ultrasound probe for 3 min, 1 sec On: 1 sec Off, amplitude 30% (total duration: 6 min). The suspension was washed with milli-Q water on a filtration system with a polypropylene membrane (0.45 µm). The washing was performed with milli-Q water with a total volume of slightly less than 1L. The wet-washed BNNTs were stripped from the membrane and the paper-like material was separated into three 5 mg batches, in 3 separate vials. Milli-Q water was added (to reach a final concentration of 1 mg/mL). The vials were closed and placed in an ultrasonic bath for 2 x 30 minutes.


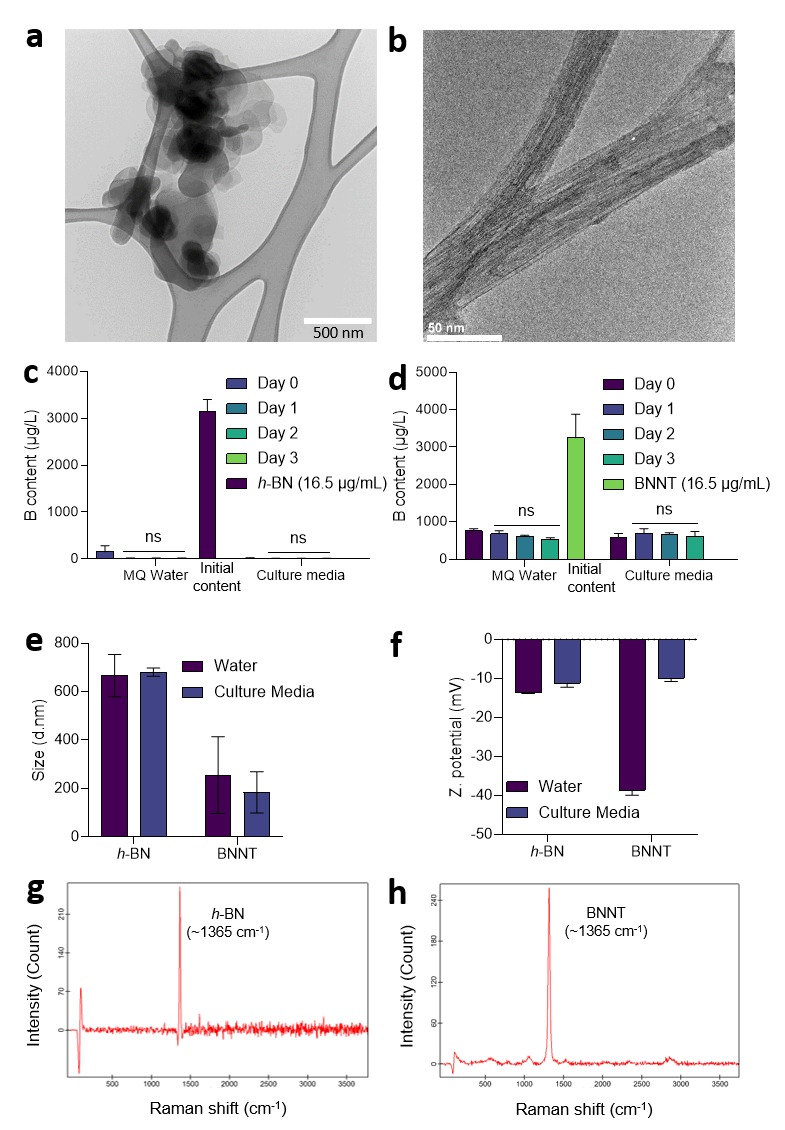


**Figure S1:** **Characterisation of *h*-BN and BNNTs.** (a-b) Representative TEM image indicates morphology and primary size of *h*-BN (a), and BNNTs (b). (c-d) Dissolution of *h*-BN (c) and BNNTs (d) in Milli-Q (MQ) water and HBE cell culture medium. The boron content (µg L^-1^) was measured in the supernatant (collected after centrifugation) after 0, 1, 2 and 3 days of incubation in respective media at 37 °C. The actual boron content in initial stock suspensions (16.5 µg mL^-1^, non-centrifuged) of *h*-BN and BNNTs was also measured and presented here. No significant (^ns^*p*>0.05) release of B ions either from *h*-BN or BNNT suspensions in MQ water or HBE cell culture medium was found with time as compared to boron content detected at day 0. (e) Hydrodynamic size of the particles in water and HBE culture medium. (f) Zeta potential of the nanomaterials in water and HBE culture medium. Data presented in (e-f) is a mean + SD of three experiments. Statistical significance was calculated by applying One-Way ANOVA and Dunnett`s post hoc test. (g-h) Raman spectrum of the *h*-BN and BNNTs indicating the presence of their characteristic peaks at ~1365 cm^−1^.


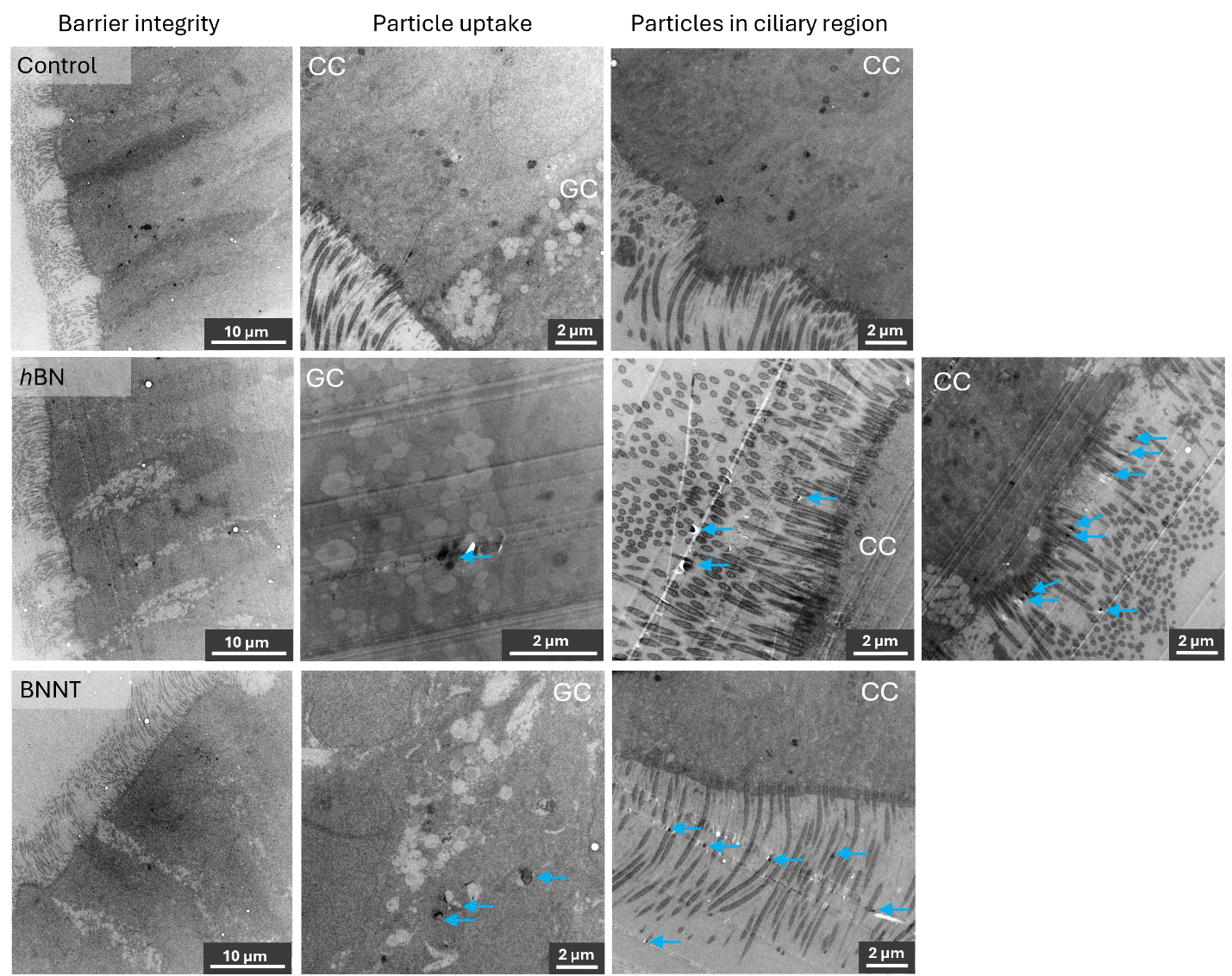


**Figure S2:** **TEM images of barrier integrity, particle uptake and particles in the ciliary region of healthy HBE cell cultures.** The representative micrographs of different regions in the TEM sections demonstrate an intact epithelial barrier in control, *h*-BN- and BNNT-exposed cells after 5 weeks. *h*-BN and BNNT particles (blue arrows) were found to be internalized preferentially by goblet cells (GC), especially *h*-BN. Furthermore, a higher occurrence of *h*-BN nanosheets in the ciliary region of different ciliated cells (CC) was detected compared to BNNTs.


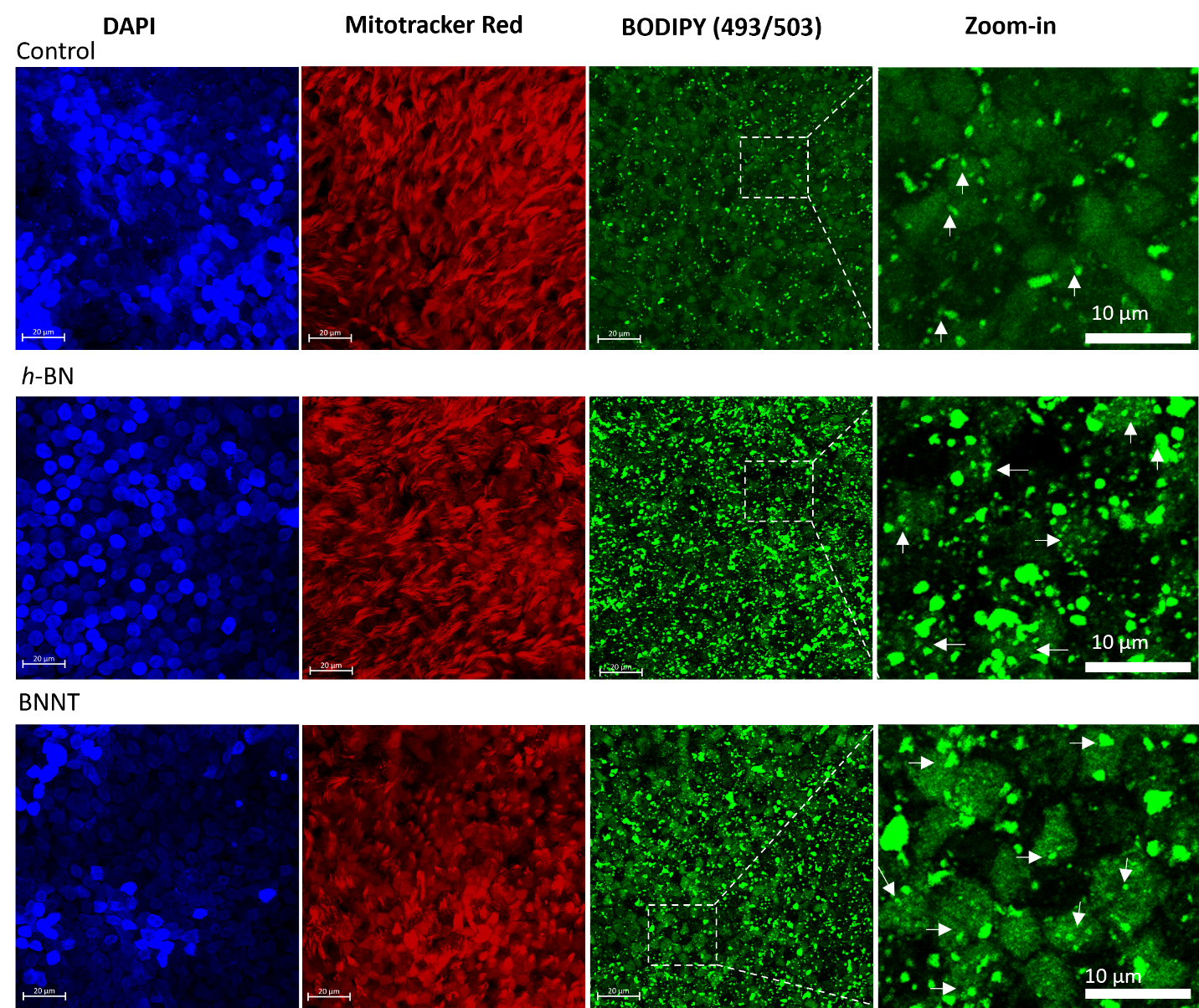


**Figure S3: Single-channel fluorescence images of healthy HBE cell cultures.** The cell cultures were stained for mitochondria (Mitotracker Red) and lipid granules (BODIPY (493/503)) after 5 weeks of exposure to *h*-BN and BNNTs. The zoomed-in image from the green channel shows the cellular accumulation of lipid granules (white arrows).


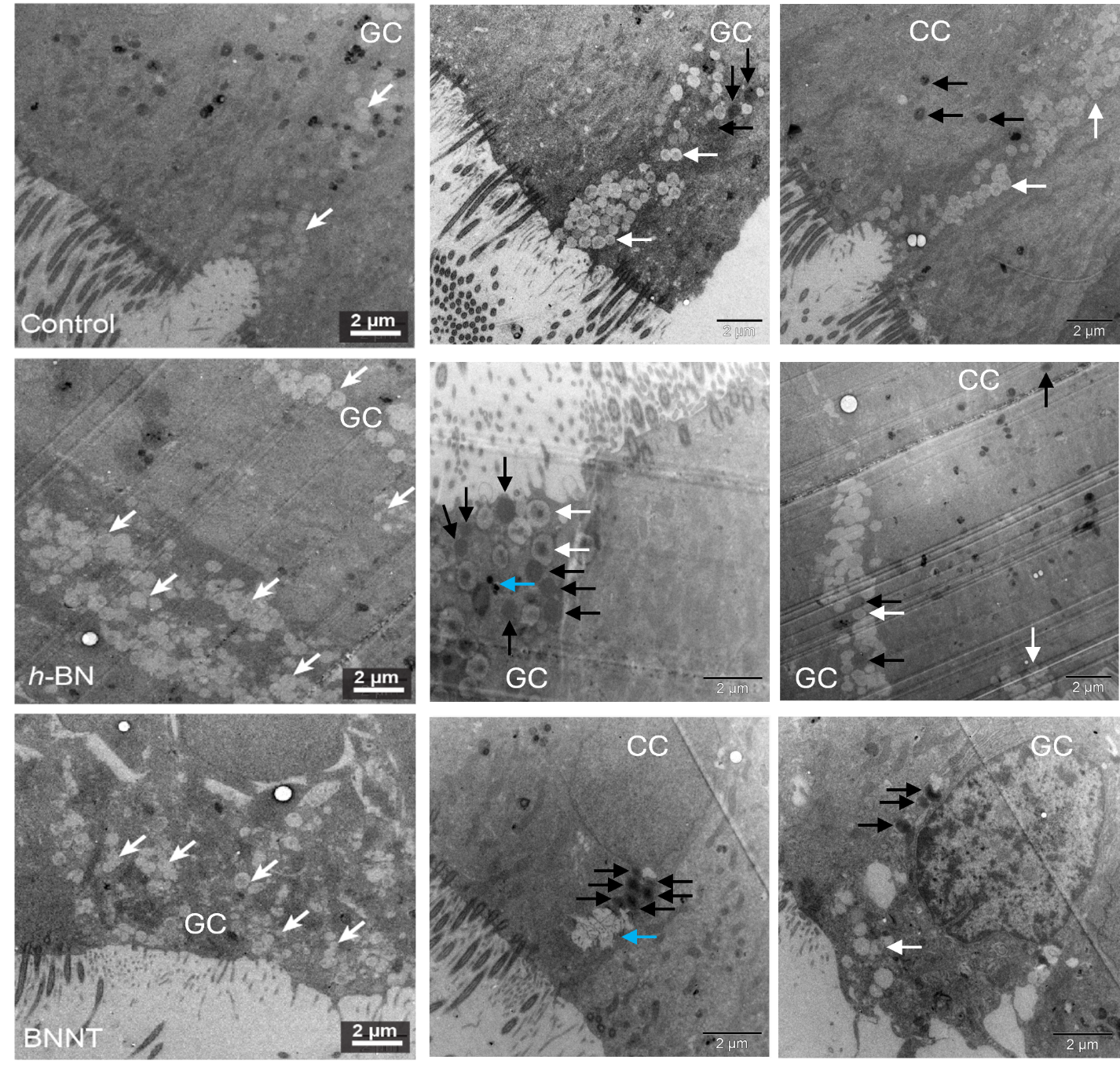


**Figure S4:** **TEM images of increased presence of mucus-secreting vesicles and lipid droplet accumulation in healthy HBE cell cultures.** The representative micrographs of different regions in the TEM sections in the first column show an increased presence of mucus-secreting vesicles (MSV, white arrows) in goblet cells (GC), which is also evident in the barrier overview micrographs in the first column in **Figure S2**. The second and third columns of TEM images demonstrate the accumulation of lipid droplets (black arrows) in goblet (GC) and ciliated cells (CC) with and without visibly internalized *h*-BN or BNNT particles (blue arrows) after 5 weeks of exposure compared to the control.


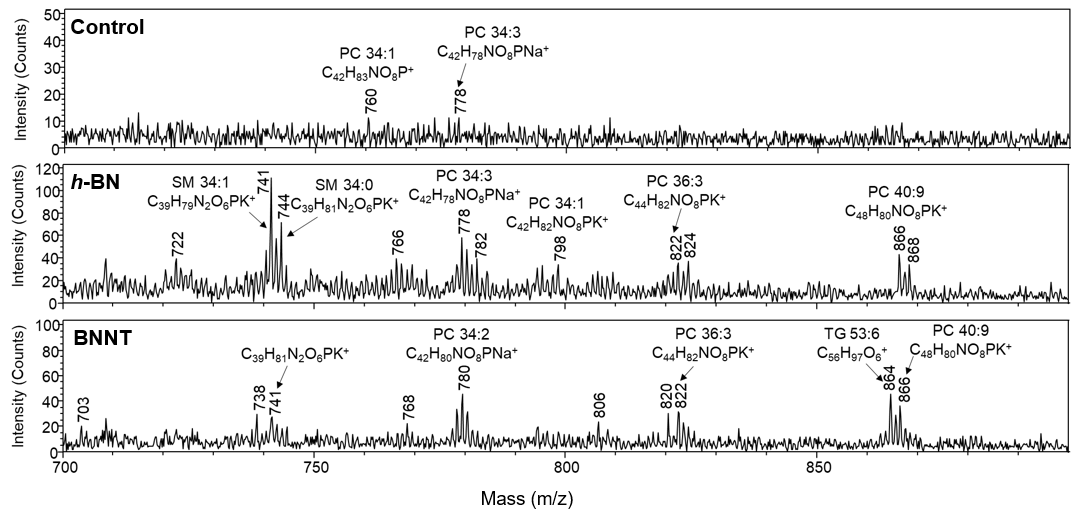


**Figure S5:** ToF-SIMS spectra of control, *h*-BN and BNNT-exposed healthy HBE cell cultures recorded in positive ion mode.


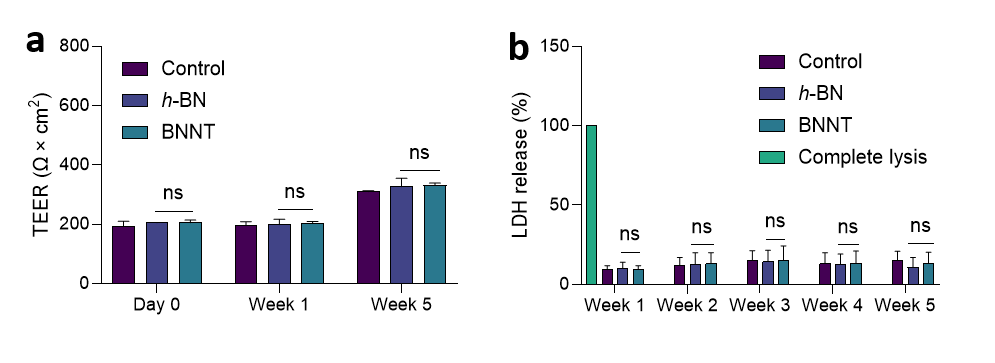


**Figure S6:** **Cytotoxicity of *h*-BN and BNNTs in asthmatic HBE cell cultures after repetitive exposure for 5 weeks.** (a) TEER, (b) LDH release. Data presented as mean + SD (n = 3). Statistical significance was calculated by applying One-Way ANOVA and Dunnett`s post hoc test. ^ns^*p*>0.05.


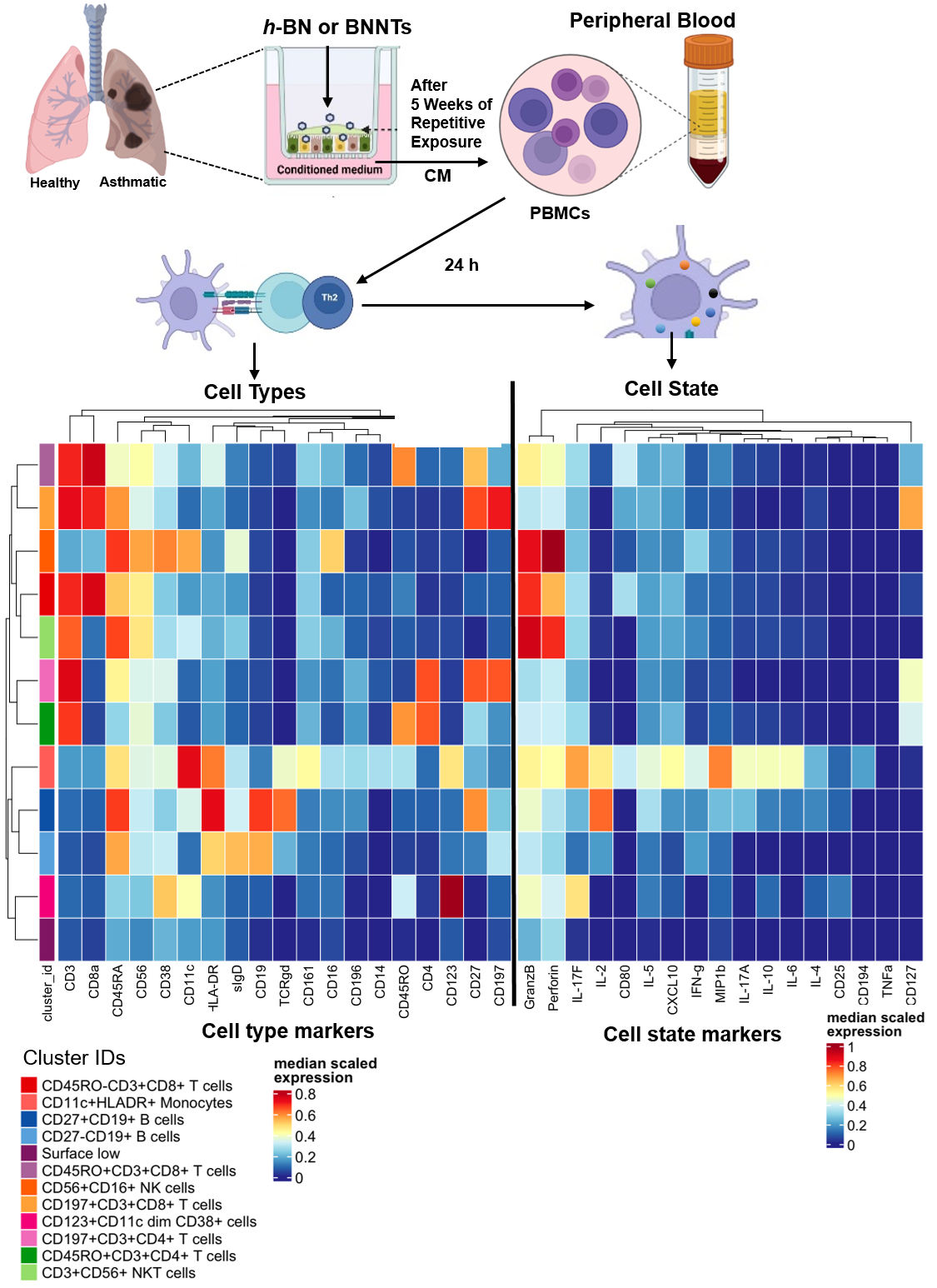


**Figure S7:** **Single-cell immune profiling of primary human PBMCs using mass cytometry (CyTOF).** Heatmap showing phenotypes (median arcsinh-transformed expression profiles for cell type markers) for the top 12 most highly significant detected cluster–marker combinations (left panel), and expression by sample for the cell state marker (signaling marker) in each detected cluster–marker combination (right panel).


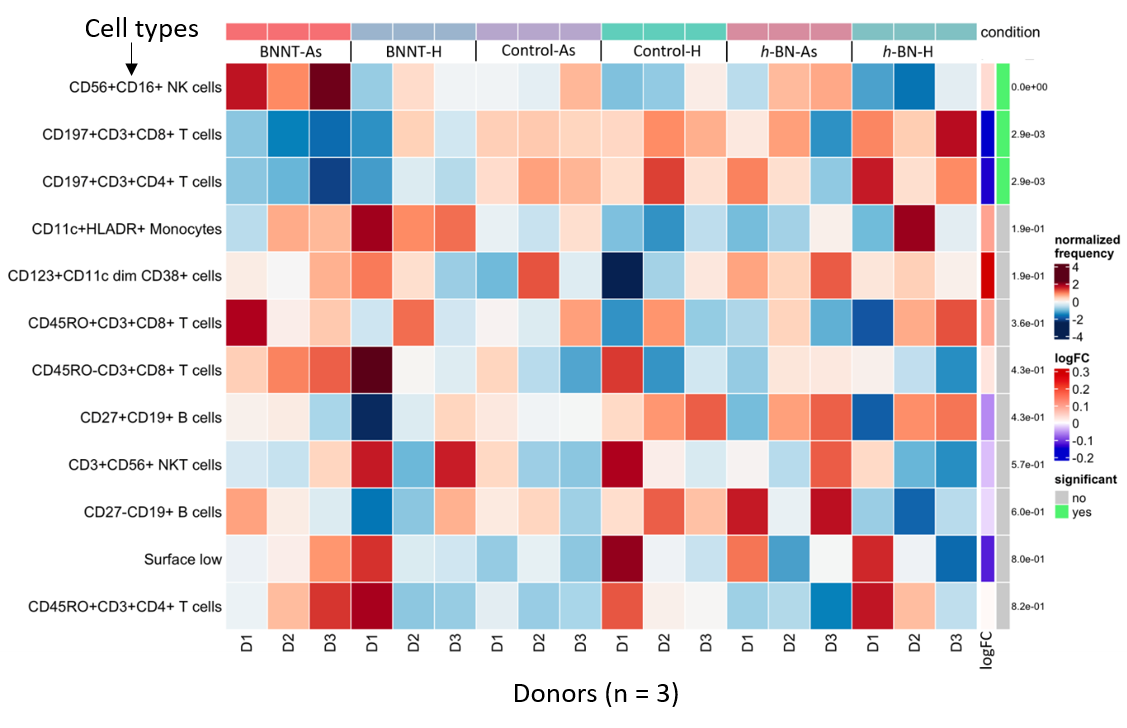


**Figure S8:** **Single-cell immune profiling of primary human PBMCs using CyTOF.** Heatmap showing expression profiles of different cell type markers in PBMCs determined using CyTOF after 24 h of exposure to conditioned media (CM) from *h*-BN or BNNT-treated healthy (H) and asthmatic (As) lung cell cultures.


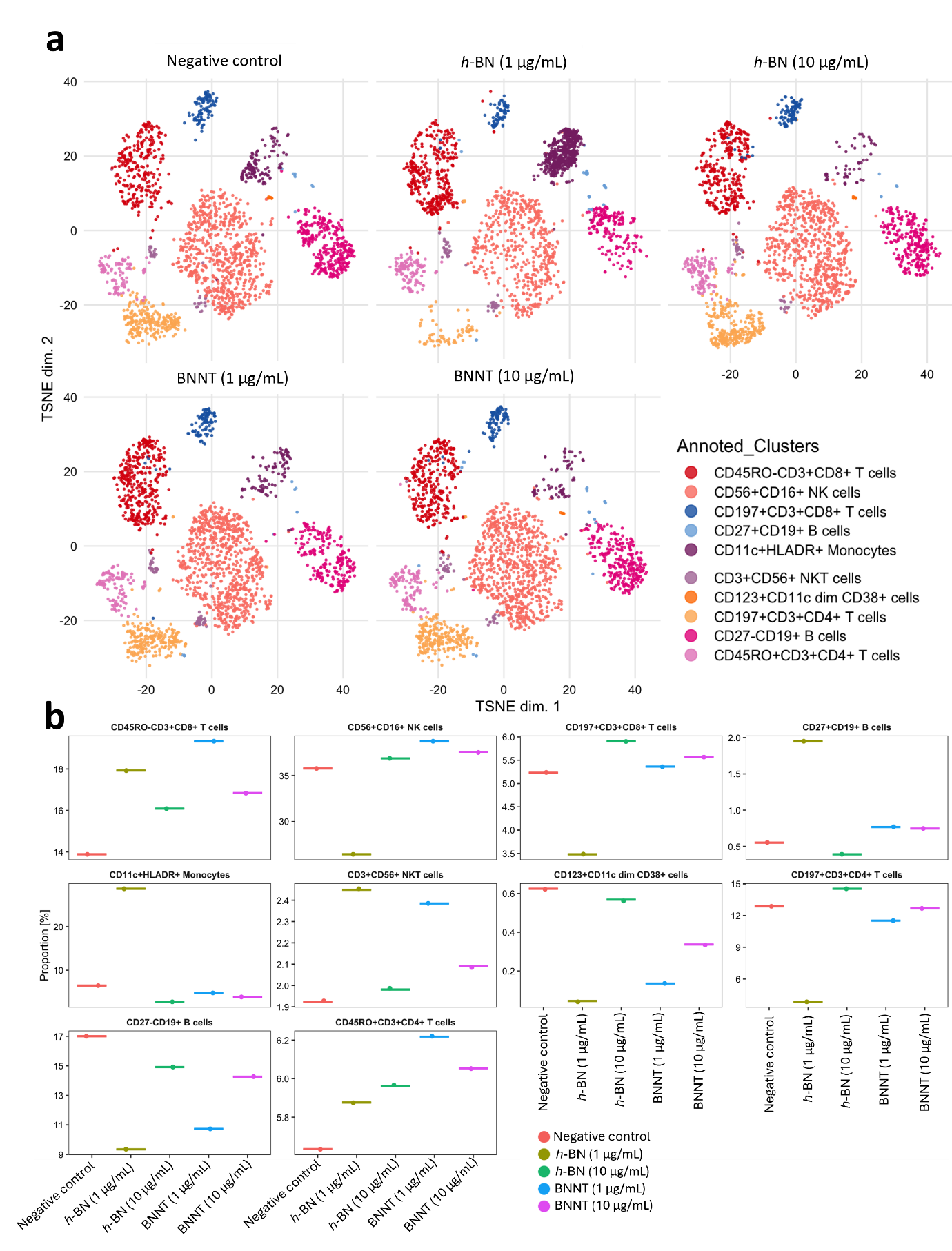


**Figure S9: Single-cell immune profiling of primary human PBMCs using CyToF after direct exposure (1 and 10 µg/mL for 24 h) to *h*-BN and BNNT**. (a) CyTOF results are represented in a T-distributed stochastic neighbor embedding (t-SNE) map with 10 clusters of cells identified in PBMCs, with notable differences observed in the abundance of CD45RO-CD3+CD8+ T cells and CD45RO+CD3+CD4+ T cells. (b) Bar plots show the relative abundance of these clusters in PBMCs after exposure to *h*-BN and BNNTs at the indicated doses. The data are presented based on results from a single donor (n = 1).


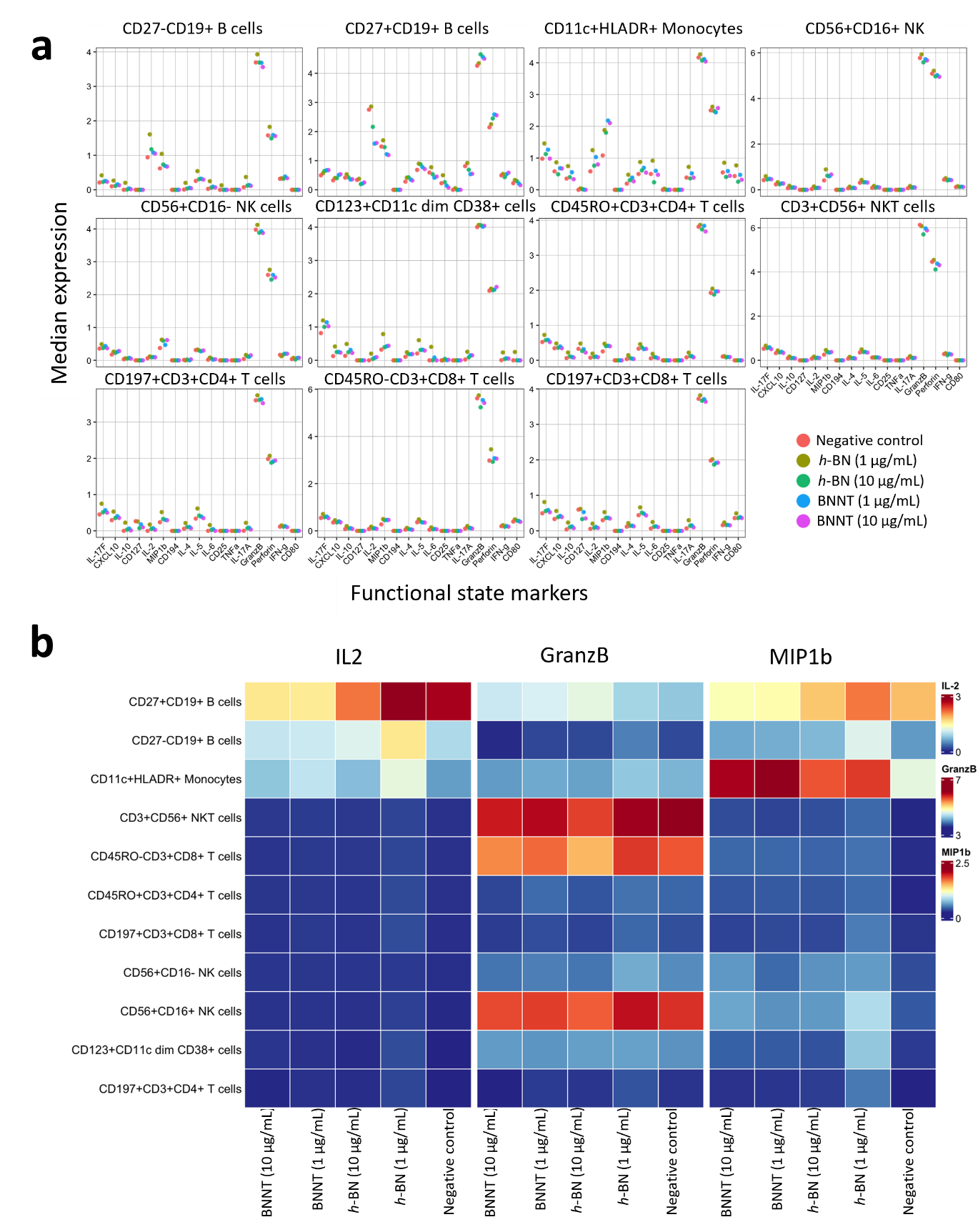


**Figure S10: Functional changes in primary human PBMCs at the single-cell level using CyTOF after direct exposure (1 and 10 µg/mL for 24 h) to *h*-BN and BNNT**. (a) Bar plots show the median expression of the indicated functional state markers at the single-cell level, annotated from PBMCs. (b) Heat map showing the affected functional markers in different cell types.


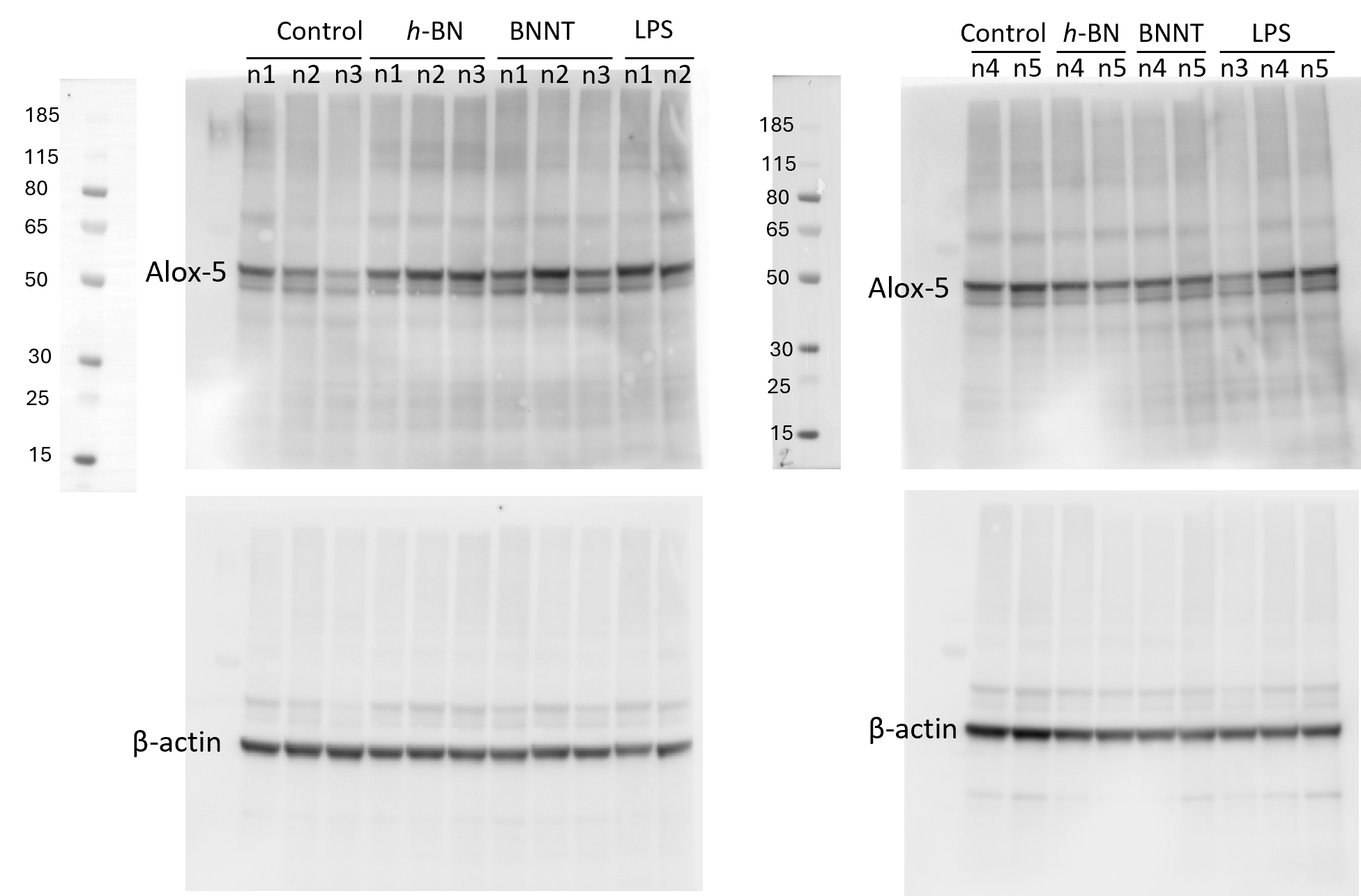


**Figure S11:** **Alox-5 protein expression level and corresponding beta-actin as loading control in mouse tissues.** The bands correspond to control, *h*-BN, BNNTs and LPS-exposed mice lung tissue (n = 5).

**Table S1:** The lipid classes affected other than the presented eicosanoid clusters after different exposures in healthy (H) and asthmatic (As) cell cultures with respect to controls.

| **Mass** | **group 1** | **group 2** | **mean diff.** | **p-adj.** | **main class** | **sub-class** |
| --- | --- | --- | --- | --- | --- | --- |
| 7.75_272.2584m/z | BNNT_H | C_H | -97521.0054 | 0.0039 | Steroid conjugates [ST05] | Glycine conjugates [ST0503] |
| 8.41_300.2896m/z | BNNT_H | C_H | -23611.3641 | 0.0269 | Fatty amides [FA08] | N-acyl ethanolamines (endocannabinoids) [FA0804] |
| 8.87_225.2213m/z | BNNT_As | C_As | 5811.0231 | 0.0061 | Fatty Acids and Conjugates [FA01] | Straight chain fatty acids [FA0101] |
| 9.08_318.2769n | BNNT_H | C_H | -75995.7315 | 0.0015 | Fatty Acids and Conjugates [FA01] | Dicarboxylic acids [FA0117] |
|  | C_H | hBN_H | 51119.9855 | 0.0281 |  |  |
| 9.37_350.3265m/z | BNNT_H | C_H | -49154.1974 | 0.0239 | Fatty amides [FA08] | N-acyl ethanolamines (endocannabinoids) [FA0804] |
| 9.27_394.3528m/z | BNNT_H | C_H | -38990.3552 | 0.0354 | Phosphosphingolipids [SP03] | Ceramide phosphocholines (sphingomyelins) [SP0301] |
| 9.30_670.4082n | BNNT_As | C_As | -60779.3622 | 0.0224 | n.a.* | n.a.* |
|  | C_As | hBN_As | 54854.9141 | 0.0422 |  |  |
| 9.46_690.4579m/z | C_As | hBN_As | 2234927.152 | 0.0137 | Ceramides [SP02] | Ceramide 1-phosphates [SP0205] |
| 8.99_362.3032n | BNNT_H | C_H | -21991.8545 | 0.0161 | Fatty Acids and Conjugates [FA01] | Carbocyclic fatty acids [FA0114] |
| 9.21_438.3791m/z | BNNT_H | C_H | -93468.8714 | 0.006 | Fatty esters [FA07] | Fatty acyl carnitines [FA0707] |
| 9.15_482.4054m/z | BNNT_H | C_H | -18549.7637 | 0.028 | Fatty esters [FA07] | Fatty acyl CoAs [FA0705] |
| 3.07_988.2626m/z | C_H | hBN_H | 381663.0579 | 0.0057 | Fatty Acids and Conjugates [FA01] | Hydroxy fatty acids [FA0105] |
| 8.13_254.1306n | BNNT_As | C_As | 5665.1861 | 0.0028 | Fatty esters [FA07] | Fatty acyl carnitines [FA0707] |
|  | C_As | hBN_As | -4995.3497 | 0.0075 |  |  |

**Lipid class or sub-class could not be assigned in LipidMaps.*

**Table S2:** The list of genes and their unique IDs used in the Prime-PCR assay for mRNA expression analysis in mouse (*Mus musculus*) lung tissue using RT-qPCR.

|  | **Gene Name** | **Gene Symbol** | **UniGene ID** | **Unique Assay Id** |
| --- | --- | --- | --- | --- |
| **Asthma biomarkers** | Arachidonate 12-lipoxygenase | Alox12 | Mm.12286 | qMmuCED0044129 |
|  | Mucin 5, subtype B, tracheobronchial | Muc5b | Mm.200752 | qMmuCID0022364 |
|  | Interleukin 13 | Il13 | Mm.1284 | qMmuCED0044968 |
|  | Phospholipase A2, group IVA (cytosolic, calcium-dependent) | Pla2g4a | Mm.4186 | qMmuCED0050610 |
|  | Arachidonate 5-lipoxygenase | Alox5 | Mm.41072 | qMmuCED0046218 |
|  | Phospholipase A2, group VI | Pla2g6 | Mm.155620 | qMmuCED0049226 |
|  | Interleukin 3 | Il3 | Mm.983 | qMmuCED0003477 |
|  | Prostaglandin-endoperoxide synthase 1 | Ptgs1 | Mm.275434 | qMmuCED0050070 |
|  | Chloride channel calcium activated 3 | Clca3 | Mm.33483 | qMmuCED0046956 |
|  | Interleukin 4 | Il4 | Mm.276360 | qMmuCED0044969 |
|  | Prostaglandin-endoperoxide synthase 2 | Ptgs2 | Mm.292547 | qMmuCED0003742 |
|  | Colony-stimulating factor 2 (granulocyte-macrophage) | Csf2 | Mm.4922 | qMmuCED0025728 |
|  | Transforming growth factor, beta 1 | Tgfb1 | Mm.248380 | qMmuCED0044726 |
|  | Cysteinyl leukotriene receptor 1 | Cysltr1 | Mm.287166 | qMmuCED0049695 |
|  | Leukotriene B4 receptor 1 | Ltb4r1 | Mm.20853 | qMmuCID0006297 |
|  | Forkhead box J1 | Foxj1 | Mm.378938 | qMmuCID0016975 |
|  | Mucin 5, subtypes A and C, tracheobronchial/gastric | Muc5ac | Mm.334332 | qMmuCED0039802 |
| **Phospholipid biosynthesis** | Choline phosphotransferase 1 | Chpt1 | Mm.288897 | qMmuCED0051525 |
|  | Diacylglycerol O-acyltransferase 1 | Dgat1 | Mm.22633 | qMmuCID0021210 |
|  | Myo-inositol 1-phosphate synthase A1 | Isyna1 | Mm.29357 | qMmuCED0003683 |
|  | Lysophosphatidylcholine acyltransferase 1 | Lpcat1 | Mm.284649 | qMmuCED0061152 |
|  | Phosphatidylethanolamine N-methyltransferase | Pemt | Mm.2731 | qMmuCID0023811 |
|  | Phosphatidylserine decarboxylase | Pisd | Mm.273765 | qMmuCED0026090 |
|  | Perilipin 2 | Plin2 | Mm.381 | qMmuCID0016776 |
|  | Phosphatidylserine synthase 1 | Ptdss1 | Mm.281464 | qMmuCID0017820 |
|  | Sphingomyelin synthase 1 | Sgms1 | Mm.329810 | qMmuCID0006068 |
|  | **Glyceraldehyde-3-phosphate dehydrogenase* | *Gapdh* | *Mm.304088* | *qMmuCED0027497* |

**housekeeping gene*

**Table S3:** Summary of biological responses measured in mouse lungs, 28 days after a single oropharyngeal aspiration of *h*-BN and BNNTs (data reproduced from the Luna et al. (2024)^16^.

| **Materials** | **Immune cells influx** | **Response**  **biomarkers** | **Histopathology** | **Fibrosis** |
| --- | --- | --- | --- | --- |
| **BNNT** | Eosinophils: +  Neutrophils: +  Multinucleated MΦ: +  Lymphocytes: + | IL-1α ↑  IFN-γ ↑  GM-CSF ↓  SAA3 ↑  ARG-1 ↑  OPN ↑ | Bronchial Thickness ↑  Pleural Thickness ↑  Granuloma ↑  DNA damage ↑ | Collagen Deposition ↑ |
| ***h*-BN** | NC | NC | NC | NC |

*(+): Present, ↓ Decreased ↑ Increased, NC: No Change; negative control used in this in vivo work: vehicle 0.5% BSA in water*

**Table S4:** Antibody panel used for PBMCs staining for CyTOF analysis.

| Use | Target | Metal | Clone | Catalog# | Company |
| --- | --- | --- | --- | --- | --- |
| Barcode | CD45 | 89Y | REA747 | 140-002-174 | Miltenyi |
| Barcode | MCB | 102Pd | n/a | 201060 | Standard Biotools |
| Barcode | MCB | 104Pd | n/a |  |  |
| Barcode | MCB | 105Pd | n/a |  |  |
| Barcode | MCB | 106Pd | n/a |  |  |
| Barcode | MCB | 108Pd | n/a |  |  |
| Barcode | MCB | 110Pd | n/a |  |  |
| surface | CD194 | 113In | REA279 | custom order | Miltenyi |
| Barcode | CD45 | 115In | REA747 | 140-002-174 | Miltenyi |
| surface | CD196 | 141Pr | REA190 | custom order | Miltenyi |
| IC | IL-4 | 142Nd | MP4-25D2 | 3142002B | Standard Biotools |
| IC | IL-5 | 143Nd | TRFK5 | 3143003C | Standard Biotools |
| surface | CD19 | 144Nd | REA675 | custom order | Miltenyi |
| surface | CD4 | 145Nd | REA623 | custom order | Miltenyi |
| surface | CD45RA | 146Nd | REA562 | custom order | Miltenyi |
| IC | IL-6 | 147Sm | REA1037 | custom order | Miltenyi |
| surface | CD16 | 148Nd | REA423 | custom order | Miltenyi |
| surface | CD25 | 149Sm | REA570 | custom order | Miltenyi |
| surface | CD123 | 150Nd | REA918 | custom order | Miltenyi |
| IC | IL-10 | 151Eu | REA842 | custom order | Miltenyi |
| IC | TNFa | 152Sm | Mab11 | 502941 | BioLegend |
| surface | CD127 | 153Eu | REA614 | custom order | Miltenyi |
| IC | IL-17A | 154Sm | REA1063 | custom order | Miltenyi |
| surface | CD27 | 155Gd | REA499 | custom order | Miltenyi |
| surface | HLA-DR | 156Gd | REA805 | custom order | Miltenyi |
| surface | TCRgd | 157Gd | REA591 | custom order | Miltenyi |
| IC | IL-2 | 158Gd | REA689 | custom order | Miltenyi |
| surface | CD197 | 159Tb | REA108 | custom order | Miltenyi |
| IC | MIP1b | 160Gd | REA511 | custom order | Miltenyi |
| surface | CD38 | 161Dy | REA572 | custom order | Miltenyi |
| surface | CD45 | 162Dy | REA747 | 140-002-174 | Miltenyi |
| surface | CD56 | 163Dy | NCAM16.2 | 3163007B | Standard Biotools |
| surface | CD161 | 164Dy | REA631 | custom order | Miltenyi |
| surface | CD45RO | 165Ho | REA611 | custom order | Miltenyi |
| IC | IL-17F | 166Er | REA1012 | custom order | Miltenyi |
| IC | CXCL10 | 167Er | REA334 | custom order | Miltenyi |
| surface | CD14 | 168Er | REA599 | custom order | Miltenyi |
| surface | Granzyme B | 169Tm | REA226 | custom order | Miltenyi |
| surface | CD3 | 170Er | REA613 | custom order | Miltenyi |
| surface | Perforin | 171Yb | dg9 | 308102 | BioLegend |
| surface | IgD | 172Yb | IA6-2 | 348235 | BioLegend |
| IC | IFNg | 173Yb | B27 | 506521 | BioLegend |
| surface | CD11c | 174Yb | REA618 | custom order | Miltenyi |
| surface | CD8a | 175Lu | REA734 | custom order | Miltenyi |
| surface | CD80 | 176Yb | REA661 | custom order | Miltenyi |
| nucleus | DNA | 191Ir | n/a | 201192B | Standard Biotools |
| nucleus | DNA | 193Ir | n/a | 201192B | Standard Biotools |
| DCE | CisPt | 198Pt | n/a | 201198 | Standard Biotools |
| Barcode | CD45 | 209Bi | REA747 | 140-002-174 | Miltenyi |
