# Supplementary material for: Boron Nitride Nanomaterials Trigger Immunomodulatory Effects in Human Broncho‐Epithelial Cells by Modulating Eicosanoid Lipid Signaling
Source: Adv Sci (Weinh). 2025 Dec 1;13(9):e16401. doi: 10.1002/advs.202516401 (PMC12904077; doi:10.1002/advs.202516401)
Supplement: Supplementary file 2 — Supporting Information [file ADVS-13-e16401-s002.pptx]

## Slide 1
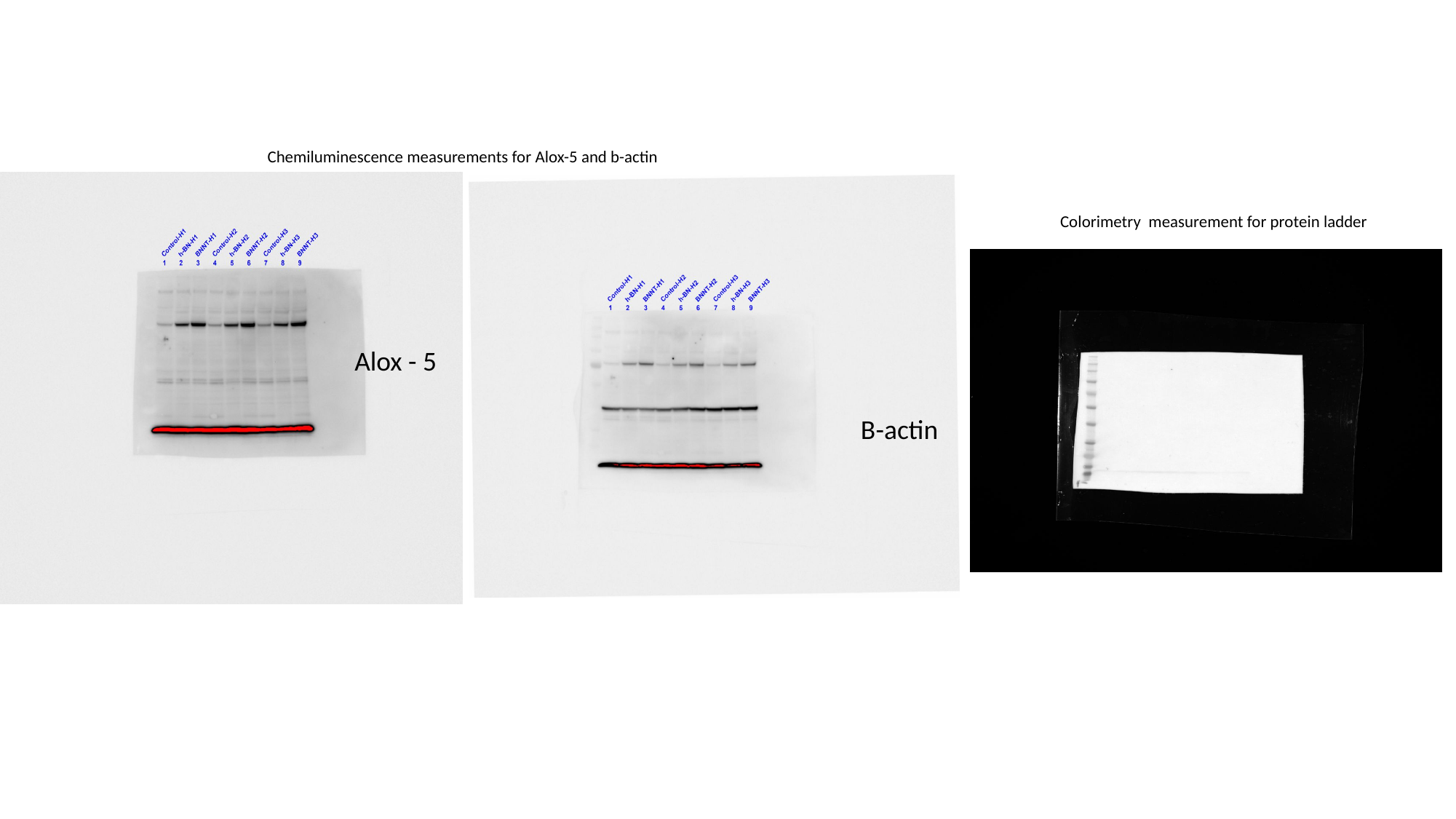

Chemiluminescence measurements for Alox-5 and b-actin
Colorimetry measurement for protein ladder
Alox - 5
Β-actin
